# On the Role of Linker Fluorination in the Adsorption‐Induced Structural Response of Ce^IV^‐Based Metal‐Organic Frameworks

**DOI:** 10.1002/chem.70830

**Published:** 2026-03-01

**Authors:** Francesca Nerli, Virginia Guiotto, Francesca Nardelli, Andrea Giovanelli, Linda Bizzarro, Federico Zizzi, Matteo Signorile, Marco Geppi, Lucia Calucci, Valentina Crocellà, Marco Lessi, Marco Taddei

**Affiliations:** ^1^ Dipartimento Di Chimica e Chimica Industriale Unità Di Ricerca INSTM, Università di Pisa Pisa Italy; ^2^ Dipartimento Di Chimica Centro NIS, Unità di Ricerca INSTM, Università di Torino Torino Italy; ^3^ Istituto Di Chimica Dei Composti Organometallici (ICCOM) Consiglio Nazionale delle Ricerche − CNR Pisa Italy; ^4^ Centro Per L'integrazione Della Strumentazione Scientifica Dell'università Di Pisa (CISUP) Università Di Pisa Pisa Italy

**Keywords:** advanced characterization, CO_2_ adsorption, flexibility, perfluorinated MOFs, s‐shaped isotherms

## Abstract

Understanding fundamental aspects of the adsorption behavior of flexible metal‐organic frameworks (MOFs) is key to developing improved sorbents for gas separations. The recently reported F4_MIL‐140A(Ce) displays cooperative CO_2_ and H_2_O adsorption driven by concerted rotation of the aromatic rings of the tetrafluoroterephthalate linkers, giving rise to a step‐shaped isotherm. Here, we shed light on the key role played by the degree of fluorination of the linker in such a mechanism by synthesizing novel F*x*_MIL‐140A(Ce) (*x* = 2 or 3) analogs and characterizing them by gas sorption analysis, in situ powder x‐ray diffraction, solid‐state nuclear magnetic resonance spectroscopy, in situ infrared spectroscopy, and adsorption microcalorimetry. We found that the cooperative CO_2_ adsorption mechanism is switched off in F*x*_MIL‐140A(Ce), leading to Langmuir‐type CO_2_ isotherms. Different from F4_MIL‐140A(Ce), CO_2_ adsorption triggers no structural response in F*x*_MIL‐140A(Ce), due to the reduced steric hindrance of less fluorinated linkers that make the Ce^IV^ open metal sites accessible already at low pressure, with no need for concerted aromatic ring rotation. In contrast, the adsorption of water induces similar cooperative structural rearrangements in F*x*_MIL‐140A(Ce) and the parent F4_MIL‐140A(Ce), suggesting that auxiliary adsorbate‐linker interactions play a role in inducing cooperative adsorption.

## Graphical abstract

The effect of the degree of fluorination of the organic linker on the adsorption behavior of MIL‐140A(Ce) is systematically investigated with a combination of experimental techniques. Cooperative adsorption is switched off in less fluorinated frameworks, as a result of reduced steric hindrance around the adsorption sites.

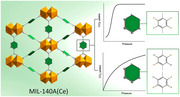

## 1 Introduction

Metal‐organic frameworks (MOFs) are a class of crystalline porous inorganic‐organic materials whose structure can be controlled at the atomic level and can respond dynamically and reversibly to external stimuli. This combination makes them promising materials across a wide range of fields, such as gas separation, sensing, and catalysis [1, 2, 3, 4, 5]. MOFs exhibiting structural rearrangements induced by gas adsorption have gained significant interest, as these rearrangements can enhance the gas separation performance by improving working capacity and selectivity, enabling more energy‐efficient processes [6, 7, 8]. Understanding the fundamental principles behind adsorption mechanisms in MOFs is essential to enable the targeted design of materials with tailored properties for specific applications.

A key aspect of investigating adsorption behavior is the description of the underlying structural dynamics, which may occur either gradually and continuously or through discrete ‘on‐off’ mechanisms. Structural transitions are often responsible for distinctive features in the adsorption isotherms, such as inflections, steps, or hysteresis, which reflect the framework's response to the adsorption of a given gas at a specific threshold pressure [9, 10]. A classification of possible flexible modes has been proposed, encompassing: (a) breathing; (b) swelling; (c) linker rotation/swing; and (d) subnetwork displacement [3, 11, 12, 13, 14, 15, 16, 17, 18, 19, 20]. Specifically, linker rotation represents a mode of flexibility, where organic linkers undergo cooperative rotations, facilitating pore window expansion and optimizing sorbent‐sorbate interactions [17, 21, 22]. This is what has been observed, for instance, for SIFSIX‐3 {with chemical formula [M(pyz)_2_SiF_6_], where M = Fe or Ni; pyz = pyrazine} pillared square grid frameworks, where a disorder‐to‐order transition is driven by the cooperative rotations of the pyrazine rings induced by sorbate–sorbent interactions [17].

**FIGURE 1:**
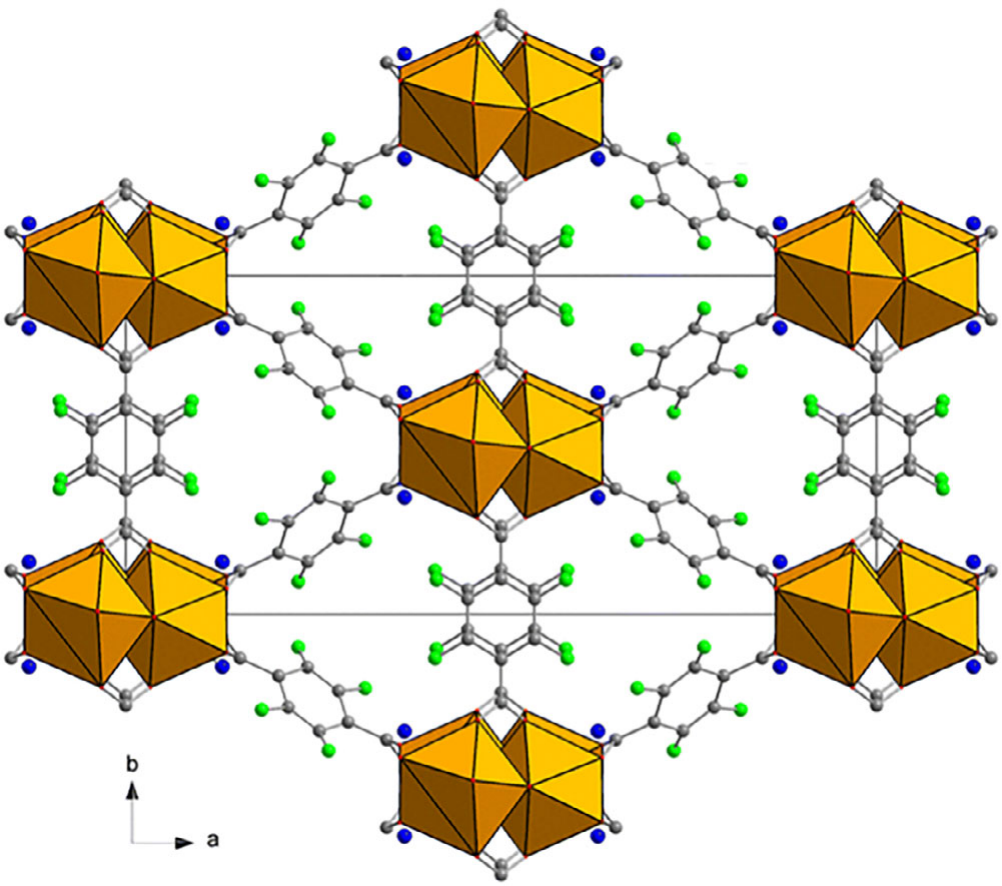
Crystal structure of as‐synthesized F4_MIL‐140A(Ce). Color code: Ce, yellow; O, red; C, grey; F, green; water, blue.

Ce^IV^‐based MOFs have been extensively developed, and a wide range of topologies have been reported. These developments have primarily been pushed by the interest in taking advantage of the redox properties of Ce^IV^, especially for catalytic applications [23, 24]. Recently, some of us have reported fundamental and application‐driven investigations on an ultramicroporous MOF, named F4_MIL‐140A(Ce), that displays a cooperative CO_2_ adsorption mechanism [25, 26, 27]. As‐synthesized F4_MIL‐140A(Ce) is made up of Ce^IV^ as the metal ion and tetrafluoroterephthalic acid (F4‐H_2_BDC) as the organic linker, with one water molecule coordinated to each Ce atom (Figure 1), rendering the formula [CeO(F4‐BDC)(H_2_O)].

The nonhysteretic, step‐shaped CO_2_ adsorption isotherm of evacuated F4_MIL‐140A(Ce) (Figure S1) leads to saturation under conditions of temperature and pressure suitable for real‐world application in biogas upgrading, as well as post‐combustion CO_2_ capture scenarios and acetylene purification. This adsorption‐induced responsiveness is attributed to a cooperative phenomenon associated to specific interactions of the framework with CO_2_, leading to exceptional CO_2_/N_2_ and CO_2_/CH_4_ selectivity and, in reverse, CO_2_/C_2_H_2_ selectivity [25, 28]. Insights from in situ crystallographic and spectroscopic techniques, and advanced sorption analysis indicated that the molecular origin of such cooperative adsorption mechanism is a CO_2_‐induced concerted ring rotation which enables CO_2_ to reach Lewis acidic open Ce^IV^ sites exposed within a tight pocket (Figure S1) [25]. A similar behavior was also observed in response to the adsorption of water, which is a stronger Lewis base that may be engaged in hydrogen bond‐like interactions with the fluorine atoms of the linkers, leading to an overall stronger interaction with the framework compared to CO_2_ [25].

Given the peculiarity of the adsorption‐induced responsiveness of F4_MIL‐140A(Ce), we herein present a fundamental study aimed at achieving a deeper understanding of the structural features responsible for such behavior. To this end, we set out to investigate the effect of the stepwise substitution of hydrogen for fluorine in the linker on the adsorption mechanism of the MIL‐140A(Ce) framework based on partially fluorinated terephthalic acids (F*x*‐H_2_BDC, where *x* = 1,2,3; Scheme S1). The substitution of hydrogen for fluorine in the linker will likely result in a lower tendency of the aromatic ring to adopt an out‐of‐plane orientation with respect to the carboxylate groups, due to the lower electrostatic repulsion between the negatively polarized O atoms of the carboxylate groups and the H atoms with respect to the F atoms, as previously observed in the literature [29]. The activation barrier for the rotation of the aromatic ring is also expected to increase at lower fluorine content, as a result of the increased conjugation between the carboxylate groups and the aromatic ring [30]. Less fluorinated linkers are more basic and might increase the stability of the Ce^IV^ − linker adduct, thus reducing the Lewis acidity of the open Ce^IV^ sites, as observed in UiO‐66(Ce) [29] and Ti_8_Ce_2_ clusters [31]. In terms of accessibility of adsorptives to such sites, the reduced length of the C─H bond compared to the C─F bond (1.1 Å vs. 1.35 Å, respectively) and the smaller covalent radius of H than F (0.31 Å vs. 0.57 Å) might lead to a reduced steric hindrance around the adsorption site. Finally, with less fluorinated linkers, the system might lack auxiliary adsorbate‐framework interactions involving F atoms that could be responsible for cooperative ring rotation: in the case of CO_2_, such interactions involve the C atom and are likely to be weak, whereas, in the case of water, stronger hydrogen bond‐like interactions are expected.

To gain an understanding of the contribution of these different factors to the cooperative adsorption behavior, we combined a range of characterization techniques, including gas sorption analysis, in situ powder x‐ray diffraction (PXRD), multinuclear solid‐state magnetic resonance (SSNMR) spectroscopy, in situ infrared (IR) spectroscopy, and CO_2_ adsorption microcalorimetry.

## 2 Results and Discussion

### 2.1 MOFs Synthesis and Preliminary Characterization

A novel synthetic strategy based on a methanol/water (MeOH/H_2_O 80:20 vol/vol) mixed solvent at 60°C was developed to obtain F*x*_MIL‐140A(Ce) materials using a range of linkers synthesized starting from commercial precursors (Schemes S1, S2, Figures S2–S14, and Tables S1, S2). In this synthetic strategy, MeOH is a polar solvent capable of dissolving both the cerium precursor and the organic linkers; however, its high solubilizing power hampers MOF precipitation and, when used alone, does not allow the formation of μ_3_‐O moieties of the characteristic one‐dimensional inorganic building units of MIL‐140A(Ce). Therefore, water was introduced as a cosolvent to modulate linker solubility and crystallization kinetics. Following a screening of reaction conditions, the 80:20 MeOH/H_2_O ratio was chosen as the optimal compromise between sample crystallinity and yield (Figure S12 and Table S1). The reaction temperature was fixed at 60°C for 1 h, as increasing the temperature to 70°C led to decreasing yields, likely because of competing Ce^IV^ reduction (Table S1).

**FIGURE 2:**
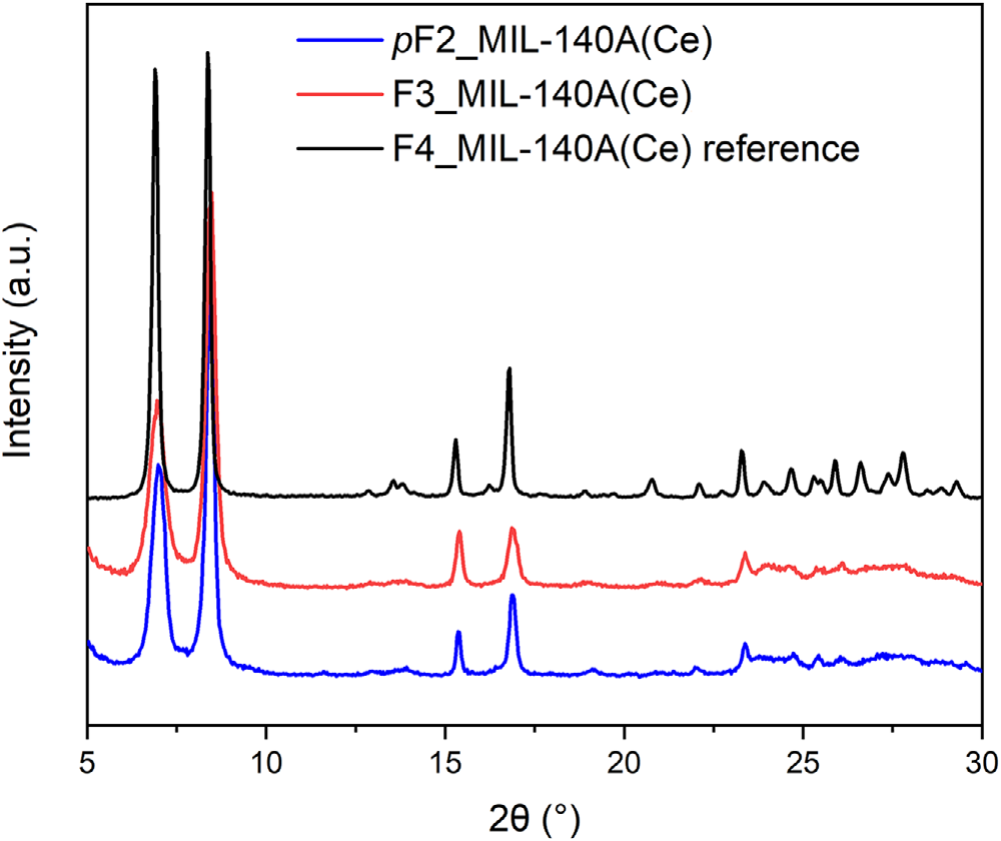
PXRD patterns of F4_MIL‐140A(Ce) reference (black), F3_MIL‐140A(Ce) (red), and *p*F2_MIL‐140A(Ce) (blue) MOFs.

The adopted synthetic strategy enabled the isolation of pure MIL‐140A(Ce) phases using F3‐H_2_BDC and *p*F2‐H_2_BDC (Figure 2), whereas attempts with F1‐H_2_BDC, *m*F2‐H_2_BDC, and *o*F2‐H_2_BDC were unsuccessful (see Table S2 and Figure S14). For a more accurate comparison and to ensure that the observed adsorption behavior originates from the intrinsic structural features of the MOFs rather than the synthetic method, the same mixed MeOH/H_2_O synthesis procedure was also used with the F4‐H_2_BDC linker, obtaining the corresponding perfluorinated framework. The synthesis conditions and the characterization of the resulting F4_MIL‐140A(Ce)_MeOH/H_2_O sample are shown in Table S3 and Figures S15–S17.

Attenuated total reflectance IR (ATR‐IR) spectra of F3_MIL‐140A(Ce) and *p*F2_MIL‐140A(Ce) resemble that of F4_MIL‐140A(Ce) reference prepared with the water‐based protocol, displaying the characteristic doublet of sharp bands between 4000–3500 cm^−1^, which indicates the presence of water coordinated to Ce (Figure S18) [25]. Additional bands in the spectra of F3_MIL‐140A(Ce) and *p*F2_MIL‐140A(Ce) arise below 1500 cm^−1^ due to the lower symmetry of the linkers compared to F4‐H_2_BDC. Thermogravimetric analysis (TGA) suggests that the coordinated water, accounting for about 4% of the mass of the MOFs, is lost around 100°C for F3_MIL‐140A(Ce) and around 80°C for *p*F2_MIL‐140A(Ce) (Figures S19–S21 and Table S4). Ill‐defined morphology with submicrometric particle size was observed for both F3_MIL‐140A(Ce) and *p*F2_MIL‐140A(Ce) by scanning electron microscopy (SEM) analysis (Figures S22, S23). ^1^H, ^19^F, and ^13^C SSNMR magic angle spinning (MAS) spectra of F3_MIL‐140A(Ce) and *p*F2_MIL‐140A(Ce) displayed the features expected for the linkers in the MIL‐140A(Ce) architecture (Figures S24, S25), while quantitative ^1^H liquidstate NMR analysis on digested F3_MIL‐140A(Ce) and *p*F2_MIL‐140A(Ce) indicated that the amount of linker in the framework of the activated MOFs is in agreement with the formula [CeO(F*x*‐BDC)] (Figures S26, S27 and Table S5).

The textural properties of F4_MIL‐140A(Ce)_MeOH/H_2_O, F3_MIL‐140A(Ce) and *p*F2_MIL‐140A(Ce) were investigated using Ar at −186°C. The three isotherms are very similar and display a composite Type Ia + Type IVa profile [32], indicating the existence of an intrinsically microporous framework along with some mesoporosity, presumably arising from interparticle voids due to the small crystallite size (Figures S17, S22, S23, and S28). According to the Rouquerol consistency criteria, the specific surface areas (SSAs) of F4_MIL‐140A(Ce)_MeOH/H_2_O, F3_MIL‐140A(Ce), and *p*F2_MIL‐140A(Ce) were determined to be 220, 207, and 213 m^2^ g^−1^, respectively, revealing no significant differences in porosity among the samples (Figures S29–S31). These values are in line with those of the F4_MIL‐140A(Ce) reference (214 m^2^ g^−1^) [25].

### 2.2 Framework Response to Adsorption

**FIGURE 3:**
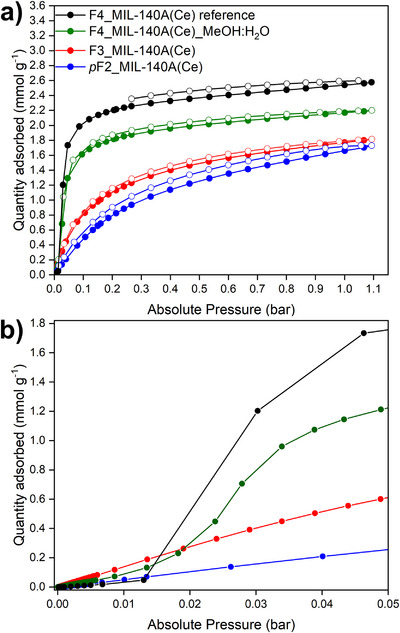
Full CO_2_ adsorption (full circles) and desorption (empty circles) isotherms collected at 0°C for F4_MIL‐140A(Ce) reference (black), F4_MIL‐140A(Ce)_MeOH/H_2_O (green), F3_MIL‐140A(Ce) (red), and *p*F2_MIL‐140A(Ce) (blue) (a). Zoom‐in of the isotherms in the low‐pressure range (b). Lines are a guide to the eye, not a fitting.

To establish the relationship between the degree of fluorination of the linker and the CO_2_ adsorption behavior of F3_MIL‐140A(Ce) and *p*F2_MIL‐140A(Ce), CO_2_ adsorption isotherms were measured at 0°C up to 1.1 bar (Figure 3). The CO_2_ isotherms of F3_MIL‐140A(Ce) and *p*F2_MIL‐140A(Ce) are clearly not step‐shaped, and their CO_2_ uptake is lower than that of F4_MIL‐140A(Ce) reference in the whole pressure range, except for pressures below 0.015 bar (Table S6). F4_MIL‐140A(Ce)_MeOH/H_2_O exhibits a step‐shaped CO_2_ adsorption isotherm, although with a lower saturation capacity compared to the F4_MIL‐140A(Ce) reference (Figure 3 and Table S6). This difference in adsorption properties between F4_MIL‐140A(Ce)_MeOH/H_2_O and the reference material can likely be ascribed to variations in morphology and crystallite size, as revealed by the PXRD pattern (Figure S15) and SEM images (Figure S17). However, since the step‐shaped profile is retained for F4_MIL‐140A(Ce)_MeOH/H_2_O, we can conclude that neither the synthetic method nor the reduced crystallite size significantly affects the cooperative CO_2_ adsorption mechanism in F4_MIL‐140A(Ce). Therefore, all comparisons from this point on are made between F*x*_MIL‐140A(Ce) and F4_MIL‐140A(Ce) reference.

Given the absence of a step in the CO_2_ isotherms of F3_MIL‐140A(Ce) and *p*F2_MIL‐140A(Ce), PXRD and SSNMR spectroscopy were employed to probe possible structural changes of these MOFs upon CO_2_ adsorption.

Since, as highlighted by Cavallo et al., the adsorption/desorption of water induces a similar concerted structural rearrangement [25], we first investigated the structural response to the dehydration process (Figures S32–S37). The variable temperature PXRD (VT‐PXRD) patterns of as‐synthesized F4_MIL‐140A(Ce) reference exhibit a shift of the 200 and 110 reflections (located at 6.9 and 8.2°2θ, respectively) to higher angles between 160°C and 200°C, indicating a phase transition associated with the removal of coordinated water (Figure S32). Similarly, F3_MIL‐140A(Ce) and *p*F2_MIL‐140A(Ce) display a shift of the same reflections toward higher angles, thereby revealing a structural rearrangement upon water removal (Figures S34–S37). Notably, these latter materials undergo such a structural change at slightly lower temperatures with respect to the perfluorinated MOF, specifically between 120°C and 160°C, highlighting weaker interactions with water molecules, in line with the weaker Lewis acidic character of their Ce^IV^ sites revealed by in situ IR spectroscopy (vide infra).

**FIGURE 4:**
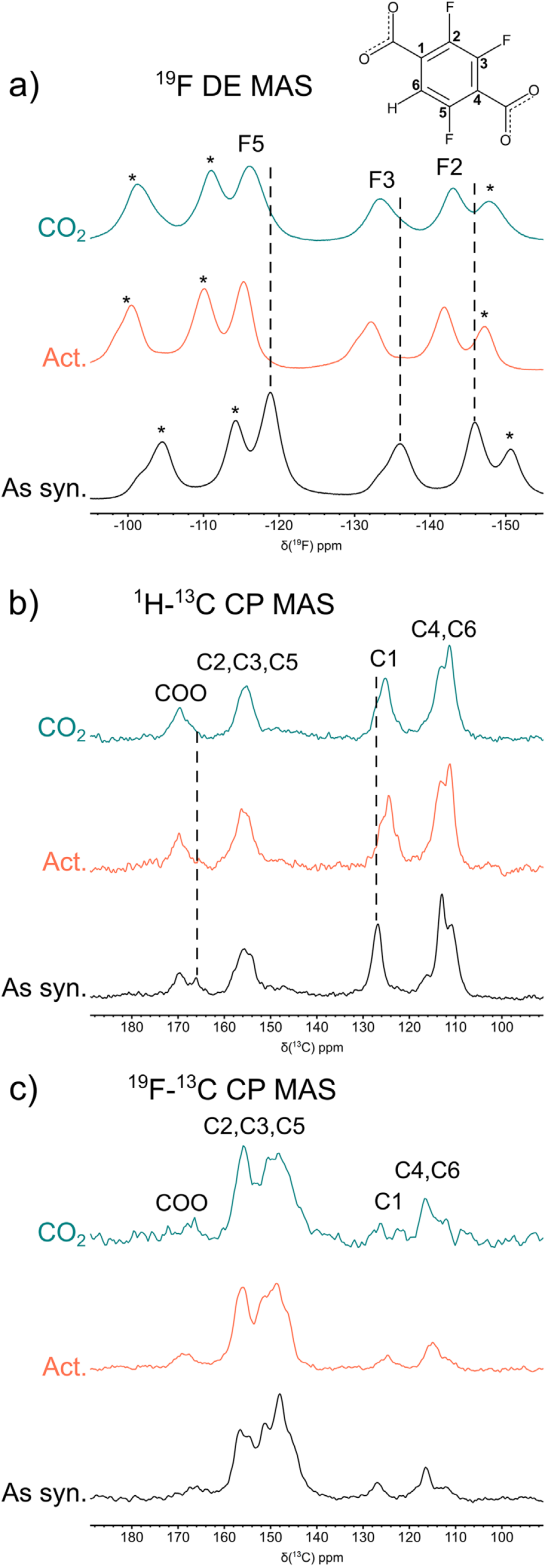
^19^F direct excitation (DE) MAS spectra (a), ^1^H‐^13^C CP MAS spectra (b), and ^19^F‐^13^C CP MAS spectra (c) of as‐synthesized (black), activated (orange), and CO_2_‐loaded (green) F3_MIL‐140A(Ce). The spectra were recorded at 25°C using a spinning frequency of 15 kHz. In ^19^F DE MAS spectra, asterisks indicate spinning sidebands.

Structural changes were also detected at the atomic level by ^19^F and ^13^C SSNMR experiments on as‐synthesized and activated F3_MIL‐140A(Ce) (Figure 4). Upon dehydration, all fluorine signals shift toward higher frequencies, indicating a significant modification of the chemical environment induced by water removal (Figure 4a). Changes are also observed in the ^1^H‐^13^C cross‐polarization magic angle spinning (CP MAS) spectra (Figure 4b) and ^19^F‐^13^C CP MAS spectra (Figure 4c), particularly for the signal of quaternary carbon C1, which shifts from 126.8 to 124.5 ppm, and for carboxylic carbons, for which only one resonance is detected at 169.9 ppm for the activated MOF, whereas two signals are observed at 169.9 and 166.2 ppm for the as‐synthesized sample. These observations suggest that the dehydration process is accompanied by structural changes, which likely involve concerted ring rotation, in agreement with VT‐PXRD findings.

**FIGURE 5:**
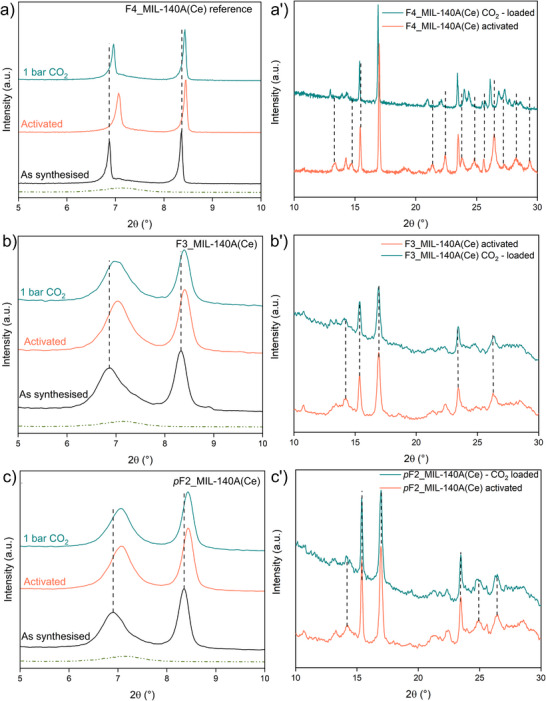
Low‐angle (a–c) and high‐angle (a′–c′) regions of PXRD patterns of F4_MIL‐140A(Ce) reference (a, a′), F3_MIL‐140A(Ce) (b, b′), and *p*F2_MIL‐140A(Ce) (c, c′). As‐synthesized samples at room temperature (black), activated samples at room temperature (orange), and after dosing 1 bar of CO_2_ inside the chamber at room temperature (green). In a−c panels, the green dot‐dashed curve represents the XRD pattern of the Kapton windows of the environmental chamber.

The structural response to CO_2_ adsorption was first investigated by in situ PXRD (Figure 5). Once 1 bar of CO_2_ is dosed on the activated F4_MIL‐140A(Ce) reference (Figure 5a), a shift of several reflections is observed, consistent with the findings previously reported by Cavallo et al. [25], confirming the occurrence of a CO_2_‐induced structural transition, leading to a phase different from both the as‐synthesized and the activated phases. In contrast, no shift is observed upon CO_2_ dosage in either activated F3_MIL‐140A(Ce) or *p*F2_MIL‐140A(Ce) (Figures 5b,c). ^19^F and ^13^C SSNMR experiments on the CO_2_‐loaded sample confirmed this behavior for F3_MIL‐140A(Ce) (Figure 4). Indeed, no significant differences were observed between the spectra of activated and CO_2_‐loaded F3_MIL‐140A(Ce), again indicating that no structural modifications occur upon CO_2_ loading. A similar conclusion can be drawn for *p*F2_MIL‐140A(Ce), based on the results of gas sorption analysis and in situ PXRD.

These results provide compelling evidence that CO_2_ is unable to trigger a concerted ring rotation in the less fluorinated frameworks, precluding the cooperative adsorption mechanism observed in the perfluorinated material. On the other hand, the observation that H_2_O desorption triggers concerted aromatic ring rotation in both F3_MIL‐140A(Ce) and *p*F2_MIL‐140A(Ce) indicates that the framework retains intrinsic structural flexibility, specifically the ability of the phenyl rings to twist, even after partial removal of fluorine atoms. The cooperative ring rotation observed in the presence of H_2_O is likely driven by hydrogen‐bond‐like interactions between water molecules and the fluorine atoms of the linker. Therefore, the absence of concerted aromatic ring rotation upon CO_2_ adsorption in F3_MIL‐140A(Ce) and *p*F2_MIL‐140A(Ce) should not be ascribed to a loss of the capability of flexibility of the framework itself, but rather to the lack of sufficiently specific interactions between CO_2_ molecules and the less fluorinated framework, which are necessary to activate the concerted linker rotation.

### 2.3 Characterization of the Adsorption Sites

**FIGURE 6:**
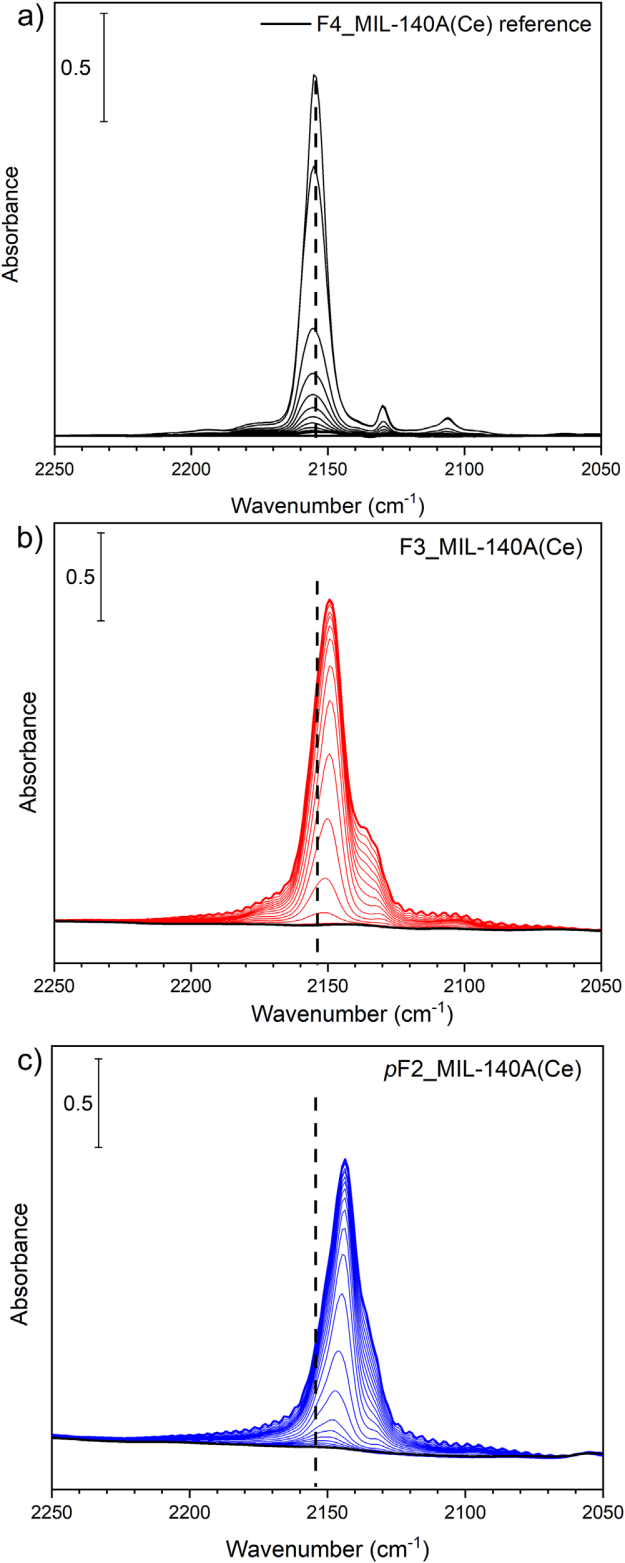
IR spectra of activated F4_MIL‐140A(Ce) reference (a), F3_MIL‐140A(Ce) (b), and *p*F2_MIL‐140A(Ce) (c) in the CO stretching range after adsorption of 60 mbar of CO at −196°C (thick line). All panels also report the subsequent outgassing sequences (thin lines) until complete outgassing was achieved (black spectrum). The dashed black line indicates the position of the Ce^IV^‐CO band in the spectrum of F4_MIL‐140A(Ce) reference.

To probe the accessibility and strength of the Ce^IV^ open metal sites, we conducted an in situ IR analysis using CO as a probe molecule, at a nominal temperature of −196°C, on activated F4_MIL‐140A(Ce) reference, F3_MIL‐140A(Ce), and *p*F2_MIL‐140A(Ce) samples. This approach, as demonstrated in the study by Cavallo et al. [25], established CO as a representative probe for the accessibility of Ce^IV^ adsorption sites in F4_MIL‐140A(Ce) reference. The IR spectra shown in Figure 6 reveal that all MOFs have accessible Ce^IV^ sites that are available for interaction with CO. Indeed, in the CO vibrational region, intense bands with apparent maxima at 2153, 2149 and 2143 cm^−1^ appear upon interaction with 60 mbar of CO for F4_MIL‐140A(Ce) reference, F3_MIL‐140A(Ce), and *p*F2_MIL‐140A(Ce), respectively. These signals can be ascribed to CO molecules interacting with Ce^IV^ sites [25, 33]. As the fluorination degree of the linker decreases, the apparent maximum of the bands associated to CO‐Ce^IV^ interactions shifts toward lower frequencies, indicating a weaker acidic nature of the Ce^IV^ sites. This effect likely arises from the higher basicity of the less fluorinated linkers, which, due to their reduced electron‐withdrawing character, donate more electron density to Ce^IV^, thereby decreasing its Lewis acidity. Moreover, the corresponding bands display a noticeable broadening, testifying to the existence of multiple components in the spectra of F3_MIL‐140A(Ce) and *p*F2_MIL‐140A(Ce). The lower symmetry of the tri‐ and di‐fluorinated linkers compared to the perfluorinated analog induces a less ordered arrangement within the framework, leading to the formation of multiple Ce^IV^ species with slightly different local environments and, consequently, acidic strength. This heterogeneity of the Ce^IV^ sites causes a broadening of the corresponding IR bands, as confirmed by the qualitative band fitting performed on the spectra of the CO‐loaded F*x*_MIL‐140A(Ce) samples (see Figures S38–S40).

The observed decrease in the CO stretching frequency proves that Ce^IV^ sites become progressively less acidic as the fluorination degree of the linker decreases, while their accessibility to CO (and potentially to smaller molecules like CO_2_ and H_2_O) is preserved. Notably, this correlates well with the strength of the Ce^IV^‐H_2_O interaction, as indicated by VT‐PXRD measurements showing a higher water desorption temperature for F4_MIL‐140A(Ce).

**FIGURE 7:**
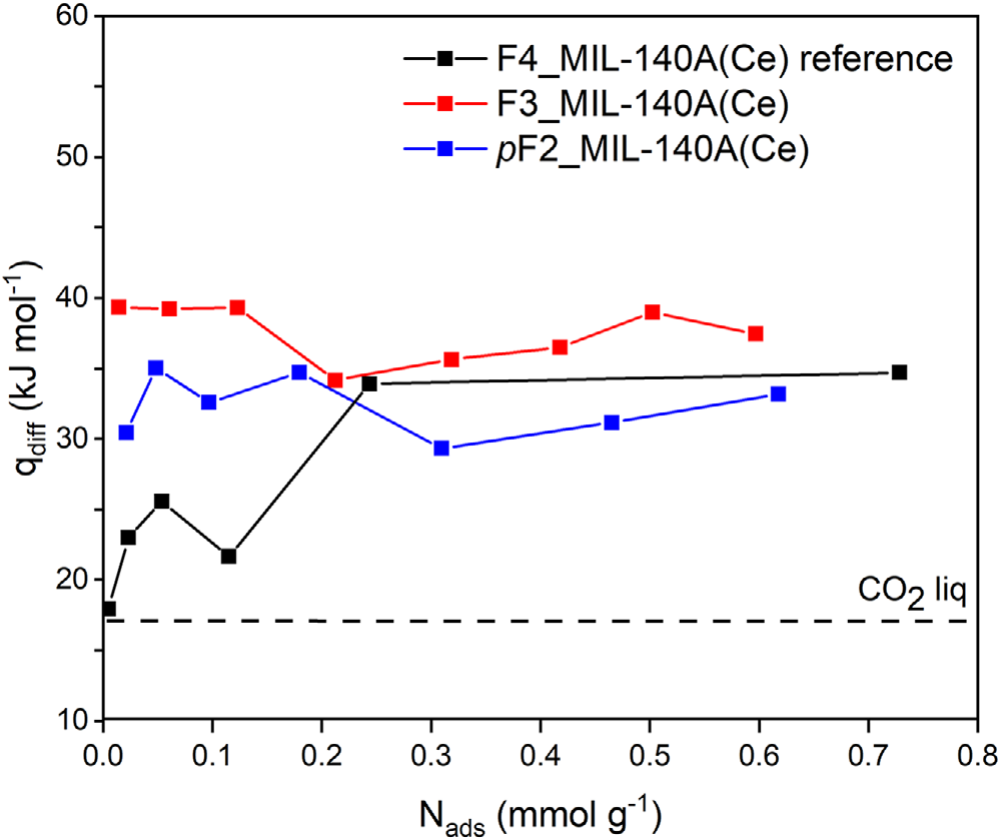
Differential heat of adsorption (q_diff_) curves of CO_2_ at 30°C of F3_MIL‐140A(Ce) (red) and *p*F2_MIL‐140A(Ce) (blue) compared to that of F4_MIL‐140A(Ce) reference (black). The dashed horizontal line indicates the standard molar heat of CO_2_ liquefaction at 30°C (17 kJ mol^−1^).

The energetics of CO_2_ adsorption in F3_MIL‐140A(Ce) and *p*F2_MIL‐140A(Ce) were investigated by performing adsorption microcalorimetry at 30°C with CO_2_ (Figures 7 and S41). The differential molar heat of adsorption (q_diff_) curves of F3_MIL‐140A(Ce) and *p*F2_MIL‐140A(Ce) are reported in Figure 7 and compared with that of the reference F4_MIL‐140A(Ce). The peculiar trend of q_diff_ of F4_MIL‐140A(Ce) was previously explained by Cavallo et al. [25] and is briefly recalled here. Before the step in the isotherm (i.e., below 0.20 mmol g^−1^), q_diff_ is close to the molar heat of liquefaction of CO_2_ (∼17 kJ mol^−1^), suggesting very weak physisorption within the channel‐like pores. Once concerted ring rotation has taken place (i.e., above 0.20 mmol g^−1^), the open Ce^IV^ sites are made accessible for interaction with CO_2_, and q_diff_ reaches a steady value of about 35 kJ mol^−1^ [25]. On the other hand, the values of q_diff_ of F3_MIL‐140A(Ce) and *p*F2_MIL‐140A(Ce) are approximately constant at 35–40 and 30–35 kJ mol^−1^, respectively, across the whole range of coverage investigated. These q_diff_ values reveal that CO_2_ interacts with exposed Ce^IV^ sites in both F3_MIL‐140A(Ce) and *p*F2_MIL‐140A(Ce) already at low coverage, suggesting that the Ce^IV^ sites are immediately accessible to the molecule, likely thanks to a reduced steric hindrance.

**FIGURE 8:**
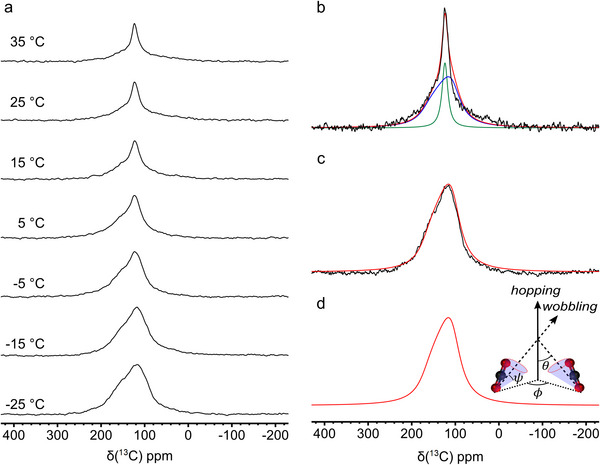
Static ^13^C NMR spectra of ^13^CO_2_ in F3_MIL140A(Ce) at the indicated temperatures (a); experimental (black) and simulated (full spectrum in red; isotropic and anisotropic sub‐spectra in green and blue, respectively) spectra at 25°C (b) and –25°C (c); simulated spectrum at –25°C considering translational hopping and local wobbling motions, as sketched in the inset, with *θ* = 81°, *φ* = 96°, and *ψ* = 40° (see Section S9 for a detailed explanation of the motional model).

To investigate the local environment and dynamics of CO_2_ in the partially fluorinated framework, we recorded static ^13^C SSNMR spectra on F3_MIL‐140A(Ce) loaded with 1 bar of ^13^CO_2_ as a function of temperature (Figure 8). An accurate analysis of the line shape of the ^13^CO_2_ signal in these spectra, reported in Section S9 of the SI, indicates that at low temperature (−25°C) CO_2_ molecules assume a unique preferential orientation in the framework and likely undergo translational hopping between Ce^IV^ sites, superimposed to local wobbling. This behavior is similar to that observed for F4_MIL‐140A(Ce) [34], although the amplitude of the local reorientation is reduced, suggesting a lower steric hindrance of the less fluorinated linkers on CO_2_ dynamics.

The anisotropic line shape does not change with temperature, indicating fast (i.e., motional rate ≥ 10^6^ s^−1^) translational hopping of CO_2_, at variance with F4_MIL‐140A(Ce) [34], for which this process becomes fast only above 20°C, suggesting a lower energy barrier for this motion in F3_MIL‐140A(Ce). At −15°C, a broad isotropic component (width at half height = 2 kHz) appears, which grows at the expense of the anisotropic one when increasing the temperature, indicating the presence of two or more local microenvironments for CO_2_ within the F3_MIL‐140A(Ce) framework. Given the Langmuir shape of the adsorption isotherm of F3_MIL‐140A(Ce), one can expect that with increasing temperature, the coverage of the Ce^IV^ adsorption sites decreases. The broadness of the isotropic line suggests that the desorbed CO_2_ likely migrates to the mesopores, rather than into the headspace of the rotor. From the trend of the ratio between the populations of CO_2_ in the two environments as a function of inverse temperature (Figure S42), an enthalpy of about 28 kJ mol^−1^ was estimated for the desorption of CO_2_ from Ce^IV^ adsorption sites at 1 bar pressure.

## 3 Conclusions

In this work, we elucidated how the degree of fluorination of the terephthalate linker influences the CO_2_‐induced structural responsiveness of Ce^IV^‐based MIL‐140A frameworks. Using a mixed methanol/water solvent, we successfully synthesized new MIL‐140A materials with F3‐H_2_BDC and *p*F2‐H_2_BDC linkers, whose textural properties closely match those of F4_MIL‐140A(Ce). However, these new MOFs do not display the step‐shaped CO_2_ adsorption isotherm observed for the perfluorinated analog, associated with a cooperative adsorption mechanism driven by a concerted rotation of the aromatic rings. In situ PXRD and SSNMR analyses confirmed the absence of rearrangements upon CO_2_ adsorption in less fluorinated frameworks, whereas adsorption/desorption of water was found to induce concerted ring rotation, as already observed for F4_MIL‐140A(Ce). Given that the H_2_O molecule is smaller than CO_2_, and that the removal of fluorine atoms reduces the steric hindrance of the linker, we infer that, in the case of water, the concerted ring rotation persists in F3_MIL‐140A(Ce) and *p*F2_MIL‐140A(Ce) due to specific interactions with fluorine atoms on the linker. The presence of open Ce^IV^ sites in F3_MIL‐140A(Ce) and *p*F2_MIL‐140A(Ce) was confirmed by in situ IR spectroscopy using CO as a probe, which also revealed a progressive decrease in Lewis acidity with decreasing fluorination. Adsorption microcalorimetry further showed that CO_2_ interacts with open Ce^IV^ sites even at low coverage, suggesting that the reduced steric hindrance around the Ce^IV^ sites allows direct CO_2_ access without requiring concerted ring rotation. SSNMR confirmed the presence of CO_2_ molecules interacting with Ce^IV^ sites and assuming a preferential orientation within the framework, as in the reference F4_MIL‐140A(Ce). The dynamics of adsorbed CO_2_ in F3_MIL‐140A(Ce), investigated by SSNMR, were found to be analogous to those previously observed in F4_MIL‐140A(Ce), although the adsorbate appears less sterically constrained in F3_MIL‐140A(Ce), most probably due to weaker interactions.

Overall, these findings highlight the pivotal role of the linker in enabling the cooperative CO_2_ adsorption mechanism observed in F4_MIL‐140A(Ce), where the presence of four fluorine atoms on the aromatic ring appears essential for triggering concerted rotation thanks to the right combination of steric hindrance, flexibility, and specific interactions with CO_2_.

We are currently exploring mixed‐linker systems by combining F4‐H_2_BDC with less fluorinated linkers, as well as investigating the replacement of Ce^IV^ with Zr^IV^, to gain a deeper understanding of the fundamental factors governing this unusual adsorption behavior.

## Author Contributions

Francesca Nerli, Virginia Guiotto, Andrea Giovanelli, Linda Bizzarro, Federico Zizzi, Marco Lessi, and Marco Taddei contributed to the synthesis. Francesca Nerli, Virginia Guiotto, Matteo Signorile, and Marco Taddei performed PXRD data collection and analysis. Francesca Nerli, Andrea Giovanelli, Linda Bizzarro, Federico Zizzi, Marco Lessi, and Marco Taddei performed liquid NMR data collection and analysis. Francesca Nerli, Andrea Giovanelli, Linda Bizzarro, and Marco Taddei performed ATR‐IR data collection and analysis. Francesca Nardelli, Andrea Giovanelli, Marco Geppi, and Lucia Calucci performed SSNMR data collection and analysis. Francesca Nerli, Virginia Guiotto, and Valentina Crocellà performed gas sorption measurements and data analysis. Francesca Nerli, Virginia Guiotto, and Valentina Crocellà carried out in situ IR spectroscopy and data analysis. Francesca Nerli, Virginia Guiotto, and Valentina Crocellà performed adsorption microcalorimetry and data analysis. Francesca Nerli wrote the first draft of the manuscript with contributions from Virginia Guiotto, Lucia Calucci, Valentina Crocellà, and Marco Taddei. Marco Taddei conceived the study and supervised the project. All authors discussed the results and commented on the manuscript.

## Conflicts of Interest

The authors declare no competing financial interest.

## Supporting information




**Supporting File 1**: Detailed synthetic procedures, PXRD patterns, adsorption isotherms, liquid state NMR spectra, SSNMR spectra, SEM images, and IR spectra. The authors have cited additional references within the Supporting Information.
