# Supplementary material for: On the Role of Linker Fluorination in the Adsorption‐Induced Structural Response of CeIV‐Based Metal‐Organic Frameworks
Source: Chemistry. 2026 Mar 1;32(19):e70830. doi: 10.1002/chem.70830 (PMC13206591; doi:10.1002/chem.70830)
Supplement: Supplementary file 1 — Supporting File 1: Detailed synthetic procedures, PXRD patterns, adsorption isotherms, liquid state NMR spectra, SSNMR spectra, SEM images, and IR spectra. The authors have cited additional references within the Supporting Information. [file CHEM-32-e70830-s001.docx]

**On the role of linker fluorination in the adsorption-induced structural response of Ce^IV^-based Metal-Organic Frameworks**

Francesca Nerli,^a^ Virginia Guiotto,^b^ Francesca Nardelli,^a,c^ Andrea Giovanelli,^a^ Linda Bizzarro,^a^ Federico Zizzi,^a^ Matteo Signorile,^b^ Marco Geppi,^a,c,d^ Lucia Calucci,^c,d,*^ Valentina Crocellà,^b,*^ Marco Lessi,^a,d^ Marco Taddei^a,d,*^

^a^ Dipartimento di Chimica e Chimica Industriale, Unità di Ricerca INSTM, Università di Pisa, Via G. Moruzzi 13, I-56124 Pisa, Italy

^b^ Dipartimento di Chimica, Centro NIS, Unità di ricerca INSTM, Università di Torino, Via G. Quarello 15, I-10135 and Via P. Giuria 7, I-10125 Torino, Italy

^c^ Istituto di Chimica dei Composti OrganoMetallici (ICCOM), Consiglio Nazionale delle Ricerche − CNR, via G. Moruzzi 1, I-56124 Pisa, Italy

^d^ Centro per l'Integrazione della Strumentazione Scientifica dell'Università di Pisa (CISUP), Università di Pisa, I-56126, Pisa, Italy

Email: [lucia.calucci@pi.iccom.cnr.it](mailto:lucia.calucci@pi.iccom.cnr.it), [valentina.crocella@unito.it](mailto:valentina.crocella@unito.it), [marco.taddei@unipi.it](mailto:marco.taddei@unipi.it)

**Supporting Information**

**TABLE OF CONTENTS**

**S1. Materials and methods**
**S2. F4_MIL-140A(Ce) reference**
**S3. Synthesis of linkers**
**S4. MOFs synthetic screening**
**S5. Preliminary characterisation of F*x*_MIL-140A(Ce)**
**S6. Variable temperature powder X-ray diffraction (VT-PXRD)**
**S7. *In situ* IR spectroscopy**
**S8. CO_2_ adsorption microcalorimetry
S9. Dynamics of CO_2_ in F3_MIL-140A(Ce) by SSNMR**
**S10. References**

**S1. Materials and methods**

*Chemicals*
Cerium ammonium nitrate (Ce(NH_4_)_2_(NO_3_)_6_, CAN), sodium sulfate (Na_2_SO_4_), celite, the heptane solution of lithium diisopropylamide (LDA), KOH, NaOH, HCl, HNO_3_, methanol (MeOH), acetone, toluene, deuterium oxide (D_2_O), deuterated dimethylsulfoxide (DMSO-d_6_), and carbon-13 enriched CO_2_ (^13^CO_2_, 99% carbon-13) were purchased from Sigma Aldrich. Potassium permanganate (KMnO_4_) was purchased from Carlo Erba. The linker 2,3,5,6-tetrafluorobenzene-1,4-dicarboxylic acid (F4-H₂BDC), organic precursors and standards 2,3,5-trifluorobenzoic acid, 3-fluoro-4-methylbenzoic acid, 2,5-difluoro-4-methylbenzoic acid, 2,3-difluoro-4-methylbenzoic acid, 3,5-difluorobenzoic acid, 2,6-difluorobenzoic acid, 2,3-difluorobenzoic acid, 2,5-difluoro benzoic acid and 2-fluorobenzoic acid were purchased from Fluorochem. Anhydrous tetrahydrofuran (THF) and diethyl ether (Et_2_O) were obtained from a solvent anhydrification system Mbraun SDS and were kept over molecular sieves 4A.

Unless otherwise specified, commercial reagents and solvents, including anhydrous solvents, were used as received, without further purification.

*Powder X-ray Diffraction (PXRD)*
PXRD patterns were collected using a step size of 0.025 °2θ and a scanning rate of 10 °2θ min^−1^ with a Rigaku MiniFlex 600-C diffractometer working in Bragg-Brentano geometry and equipped with a D/teX detector, using Cu K*α* radiation (1.54056 Å). The X-ray tube was operated at a voltage of 40 kV and a current of 15 mA. Variable temperature PXRD patterns were collected using an Anton Paar BTS-500 chamber. Samples were heated at 40 °C, 80 °C, 120 °C, 160 °C and 200 °C with a ramp rate of 5 °C min^−1^ and held at each temperature for 10 minutes to allow for equilibration prior to pattern collection. A pattern was also collected after the heating ramp, when the temperature of the chamber had returned to 40 °C.

PXRD patterns under CO_2_ atmosphere were collected in Bragg-Brentano geometry on a Panalytical Empyrean diffractometer, equipped with a Cu Kα source and a PIXcel3D areal detector. Temperature/atmosphere control was achieved with an Anton-Parr HTK 1200N environmental chamber with a motorized z-stage, allowing for real-time correction of the sample vertical position to minimize z displacement due to the thermal expansion of the sample holder. The sample was heated under vacuum (residual pressure < 10^-2^ mbar) up to 120 °C with a rate of 5 °C/min, then was kept in isothermal conditions for 1 h. Afterwhile, the sample was cooled back to 30 °C and ca. 1 bar of CO_2_ was admitted to the chamber. Finally, the sample was outgassed for 1 h at room temperature. XRD patterns were collected in the 3-30° range with a 0.013° step at relevant points of the thermal protocol.

*Attenuated total reflectance infrared spectroscopy (ATR-IR)*
ATR-IR spectra were recorded with an Agilent Cary 630 FTIR spectrometer equipped with a ZnSe crystal. Each spectrum was recorded at 25 °C within the 4000-648 cm^-1^ spectral range, with a resolution of 4 cm^-1^ and accumulating 16 scans.

*Solid State NMR (SSNMR) spectroscopy*SSNMR spectra were recorded on a Bruker Avance Neo 500 spectrometer working at Larmor frequencies of 500.13, 470.59, and 125.77 MHz for ^1^H, ^19^F, and ^13^C nuclei, respectively, equipped with a 4 mm double-resonance cross polarization – magic angle spinning (CP-MAS) probe. ^1^H and ^19^F spectra were acquired under MAS using direct excitation (DE) experiments with 90° pulse duration of 4.3 and 3.5 μs, respectively; 16 scans were accumulated with recycle delays of 1-2 and 2-10 s for ^1^H and ^19^F spectra, respectively. ^13^C spectra were recorded using either ^1^H-^13^C CP-MAS or ^19^F-^13^C CP-MAS experiments under high power decoupling from ^1^H or ^19^F, respectively. Contact time values ranging from 0.5 to 8 ms were used, while the recycle delay was 1-2 s and 1600-2400 scans were accumulated. All spectra were acquired at room temperature at a spinning frequency of 15 kHz using air as spinning gas. The chemical shift scale was referenced to the signal of adamantane resonating at 38.48 ppm for ^13^C and calculated from the same value for all the other nuclei using the unified scale recommended by IUPAC.^1^

^13^C DE spectra under static conditions were recorded on ^13^CO_2_-loaded F3_MIL-140A(Ce) using a recycle delay of 4 s and accumulating 2000 scans.

The activated and ^13^CO_2_-loaded samples for SSNMR measurements were prepared using a home-made cell provided with a mechanical lever operated from outside enabling the capping of the rotor without disturbing the cell atmosphere. For activated samples, MOF powder, packed into the NMR rotor (4 mm external diameter), was evacuated inside the cell by heating 6-8 h under vacuum (0.1 mbar) at the temperature of 140 °C and then the rotor was closed. For the preparation of the ^13^CO_2_-loaded sample, the cell containing the activated sample was loaded with ^13^CO_2_ at 1 bar pressure at RT and the rotor was capped after equilibration under the gas atmosphere.

The ^13^C static spectra of ^13^CO_2_-loaded F3_MIL-140A(Ce) were deconvoluted using the solid line shape analysis tool “SOLA” of the Bruker software *TopSpin*. The EXPRESS (Exchange Program for Relaxing Spin Systems) software^2^ was used to simulate the anisotropic component the spectra. The ^13^C NMR CSA parameters of rigid CO_2_^3,4^ were used as input parameters in the software. Powder averaging was performed over 8096 powder increments using the ZCW powder averaging procedure. The spectral width was set to 200 kHz, and 1024 data points were used in the calculated free induction decay.

*Liquid State NMR spectroscopy*

Liquid state NMR experiments were performed at 25 °C on an NMR JEOL YH spectrometer operating at the ^1^H and ^19^F Larmor frequencies of 399.78 and 376.17 MHz, respectively, and on an NMR JEOL CZR spectrometer operating at the ^1^H and ^19^F Larmor frequencies of 500.159 and 470.620 MHz, respectively. Chemical shifts, expressed in ppm from tetramethylsilane, were referenced to residual solvent peaks. Quantitative ^1^H NMR spectra of digested MOFs were acquired with a recycle delay of 4 s and 4 scans, while ^19^F NMR spectra were recorded with a recycle delay of 25 s and 16 scans. The sample treatment prior to MOFs digestion is described in section S5.

*Scanning Electron Microscopy (SEM)*Field Emission SEM (FESEM) images of F4_MIL-140A(Ce) reference were acquired using a TESCAN S9000G FE-SEM 3010 microscope (30 kV), equipped with a Schottky type FEG source. Prior to analysis the samples were metallised with a chromium layer. FESEM images of F4_MIL-140A(Ce)_MeOH:H_2_O, F3_MIL-140A(Ce), and *p*F2_MIL-140A(Ce) were acquired with a JEOL 7800F FEG SEM instrument with an acceleration voltage of 10 kV. The powders were deposited on carbon sticky tape on an aluminum support and sputtered with platinum with a Leica EM ACE600 sputterer, using an ultimate vacuum ≤2 × 10^−6^ mbar.

*Thermogravimetric analysis (TGA)*TGA was performed in air with a TA Instrument Thermo balance model Q5000IR using 2 - 4 mg of sample, with a heating rate of 5 °C min^-1^ up to 600 °C.

Gas sorption analysis
N_2_ adsorption isotherms (at –196 °C) and Ar adsorption/desorption isotherms (at –186 °C) were collected using a Micromeritics 3Flex adsorption analyser. All samples were previously activated by heating about 40 mg of powder at 80 °C under dynamic vacuum overnight. The Specific Surface Area (SSA) of each sample was evaluated starting from the experimental Ar isotherm by fitting the Brunauer-Emmett-Teller (BET) equation in the 0.005 – 0.1 p/p_0_ range, according to Rouquerol consistency criteria.^5^ CO_2_ and CH_4_ adsorption/desorption isotherms were collected at 0 °C using a Micromeritics ASAP2020 adsorption analyser. To keep isothermal conditions for each analysis, the sample was inserted in a home-made patented glass coating cell in which a coolant or heating fluid, connected to a thermostatic bath (JULABO F25), can recirculate.^6^ For each analysis, about 70 mg of sample was weighed and activated at 80 °C under dynamic vacuum overnight.

*In situ Infrared (IR) spectroscopy*
*In situ* IR spectra were recorded within the 5000-500 cm^-1^ spectral range using a Bruker Vertex 70 spectrophotometer, equipped with a MCT (mercury cadmium tellurium) cryogenic detector. The resolution of the spectra was 2.0 cm^-1^ and an average of 32 scans was used to increase the signal to noise ratio. Before the analysis, the sample, in form of self-supported pellet mechanically protected by a gold envelope, was inserted in a special home-made quartz cell with KBr windows. The cell was connected to a conventional high-vacuum glass line, equipped with mechanical and turbo molecular pumps (residual pressure p < 10^−4^ mbar). The samples were activated in vacuum at 80 °C for 12 h. To follow the interaction of carbon monoxide with the sample at cryogenic temperature, a specific home-designed IR cell was used, filled with liquid nitrogen to decrease the sample temperature to a nominal value of −196 °C. The spectra obtained at about 2 mbar CO pressure (where the rotovibrational profile of gaseous CO was absent) were fitted with the program *OriginLab*. The positions of overlapped bands were determined from the minima in the second derivative of the spectrum. Band fitting of CO loaded F4_MIL-140A(Ce) reference, F3_MIL-140A(Ce), and *p*F2_MIL-140A(Ce) was performed with Voigt functions.

*Adsorption microcalorimetry*CO_2_ adsorption microcalorimetry was performed at 30 °C by means of a heat flow microcalorimeter (Calvet C80 by Setaram) connected to a high-vacuum (≈10^−4^ mbar) glass line equipped with a Varian Ceramicell 0–100 mbar gauge and a Leybold Ceramicell 0–1000 mbar gauge. The samples were activated under vacuum at 80 °C for 12 h before being placed into the calorimeter under isothermal conditions. The measurements were carried out following a well-established procedure.^7^ The CO_2_ adsorption was performed step by step, admitting incremental doses of CO_2_ into the microcalorimeter cell by employing a volumetric apparatus. For each gas dose, the pressure drop in the known volume was monitored alongside the heat released until thermal equilibrium was reached. This procedure allows, during the same experiment, the simultaneous determination of both the integral heat evolved (−Q_int_) and the adsorbed amount (N_ads_) for small increments of adsorptive pressure. The heat of adsorption values obtained for each small dose of gas admitted over the sample (−q_diff_) are reported as a function of coverage to obtain the (differential) enthalpy changes related to the proceeding adsorption processes. The differential heat plot was obtained by taking the middle point of the partial molar heat (ΔQ_int_/ΔN_ads_, kJ mol^−1^) vs. N_ads_ histogram relative to the individual incremental dose.

**S2. F4_MIL-140A(Ce) reference**


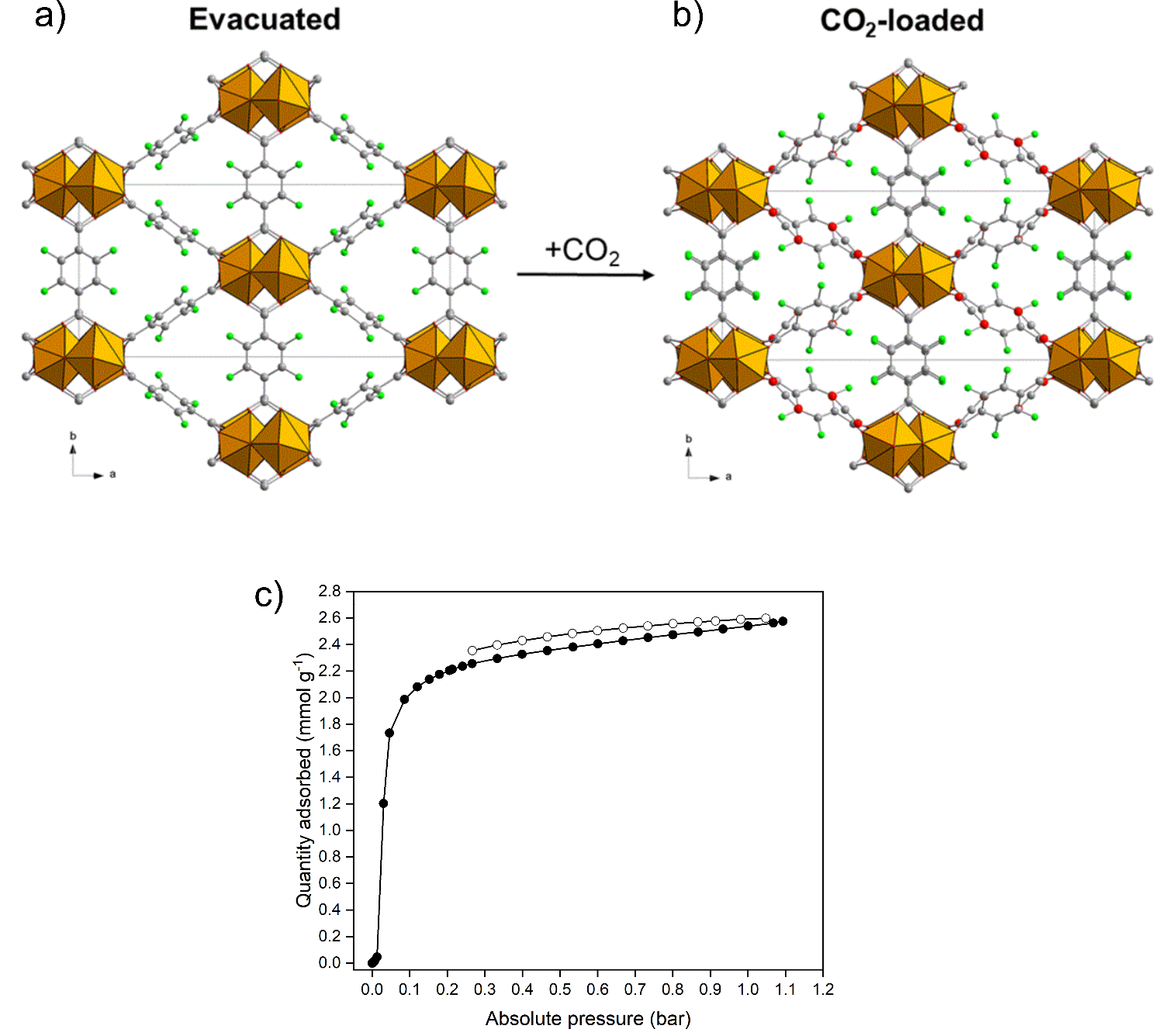


**Figure S1.** Crystal structures, viewed along the *c* axis, of evacuated (a) and CO_2_-loaded (b) F4_MIL-140A(Ce). CO_2_ adsorption (full circles) and desorption (empty circles) isotherms collected at 0 °C (c). By comparison of evacuated and CO_2_ loaded structures, a cooperative ring rotation can be observed upon CO_2_ adsorption.^8^

**S3. Synthesis of linkers**


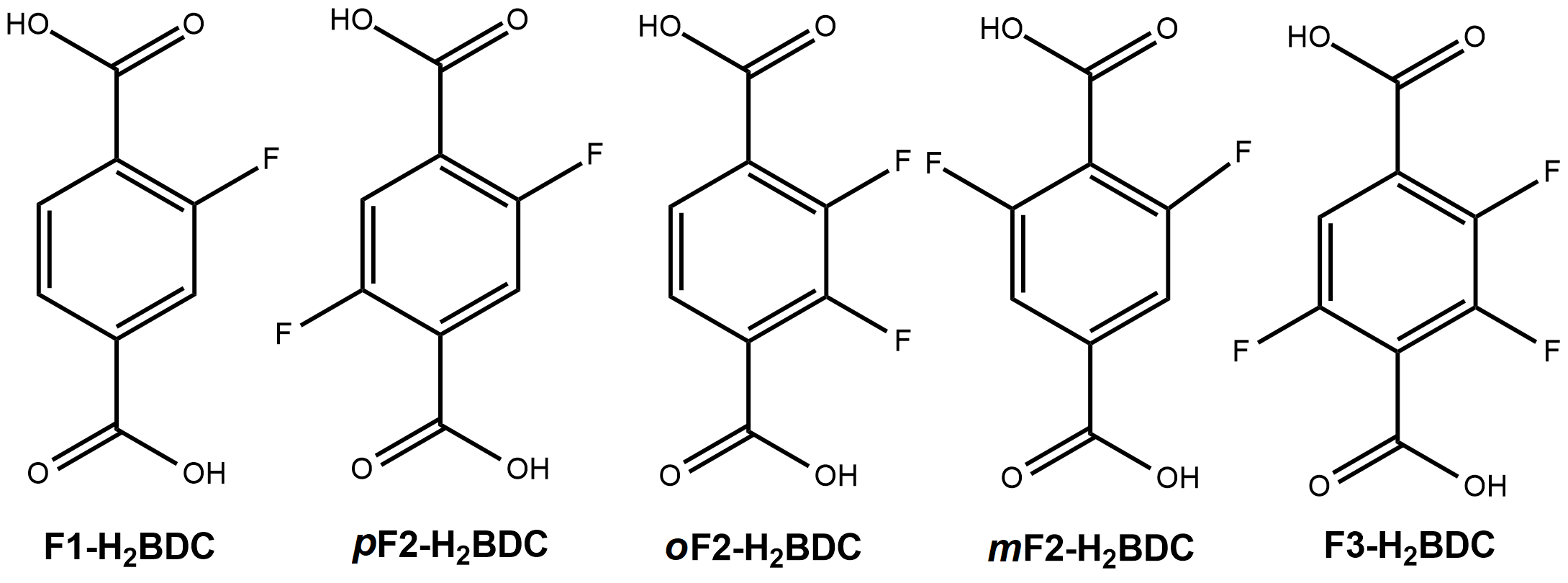


**Scheme S1**. Fluorinated terephthalic acids F*x*-H_2_BDC employed as organic linkers for the synthesis of novel MIL-140A(Ce) MOFs. *x* indicates the fluorination degree of the ring, while the *o,* *p*, and *m* prefixes identify the three different isomers of the difluorinated linkers.

Each organic linker was purposely synthesised according to the methods described in Scheme S2. A modification of an *ortho*-lithiation procedure reported in the literature^9^ allowed 2,3,5-trifluoroterephthalic acid (F3-H_2_BDC) and 2,6-difluoroterephthalic acid (*m*F2-H_2_BDC) to be successfully prepared starting from the respective commercial fluorinated benzoic acids. In contrast, an oxidation procedure^10^ was used to obtain 2-fluoroterephthalic acid (F1-H_2_BDC), 2,3-difluoro terephthalic acid (*o*F2-H_2_BDC), and 2,5-difluoro terephthalic acid (*p*F2-H_2_BDC).

**
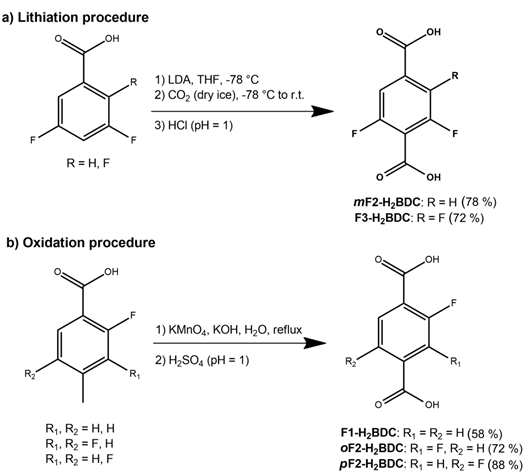
**

**Scheme S2.** Lithiation (a) and oxidation (b) procedures adopted for the synthesis of F*x*-H_2_BDC linkers.

*Synthesis of 2,3,5-trifluoro terephthalic acid, F3-H_2_BDC*^9^


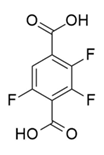


According to the literature,^9^ 1 equivalent of 2,3,5-trifluorobenzoic acid (22.7 mmol, 4 g) was dissolved in 80 mL of anhydrous THF. The solution was stirred and cooled down to −78 °C under argon atmosphere, and 2 equivalents of 1.8 M heptane solution of LDA (45.4 mmol, 25.2 mL) was added dropwise. The solution turned into a red dispersion.

To analyse the reaction, 1 mL of the mixture was taken and quenched with a diethyl ether solution of I_2_ followed by treatment with HCl 10% w/w and Na_2_S_2_O_3_.The organic phase was then analysed by UPLC-MS to determine the presence of unreacted precursor. An additional equivalent of LDA (22.7 mmol, 12.6 mL) was added to the mixture, which was left to react for 1 hour.

An excess amount of dry ice was then added under an argon flow, allowing the reaction to reach room temperature overnight. The reaction was then quenched with HCl 10% w/w until the pH reached 1, resulting in a biphasic system. The organic phase was extracted with diethyl ether (3 x 25 mL), washed with water (2 x 15 mL) and brine (1 x 20 mL), and then dried over Na_2_SO_4_, filtered, and the solvent was removed under reduced pressure.

The obtained reddish solid was treated with refluxing toluene for two hours and then filtered, yielding 3.6 g of F3-H_2_BDC (yield: 72%).

**^1^H NMR (400 MHz, DMSO-d6)** δ 14.25 (s, 2H), 7.85-7.34 (m, 1H) ppm.

**^19^F NMR (376 MHz, DMSO-d6)** δ -116.53 (dd, J = 15.4 Hz, 9.4 Hz, 1F), -134.25 (d, J = 22.0 Hz, 1F), -139.73 (t, J = 19.2 Hz, 1F) ppm.

*Synthesis of 2,6-difluoro terephthalic acid, mF2-H_2_BDC*^9^


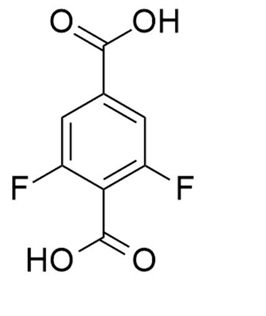


The same synthetic procedure described for F3-H_2_BDC was used to synthesise *m*F2-H_2_BDC, starting from 1 equivalent of 3,5-difluoro benzoic acid (18.8 mmol, 3 g), 66 mL of THF and 3 equivalents of 2 M heptane solution of LDA (56 mmol, 28 mL).
A white solid was isolated (3 g), yield: 78%.

**^1^H NMR (400 MHz, DMSO-d6)**: δ 14.08 (s, 2H), 7.65 (d, J = 8.4 Hz, 2H) ppm.

**^19^F NMR (376 MHz, DMSO-d6):** δ -111.05 (d, J = 8.5 Hz, 2F) ppm.

*Synthesis of 2,3-difluoro terephthalic acid, oF2-H_2_BDC*^10^


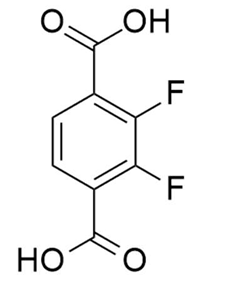


Following a synthetic strategy similar to that reported in literature,^10^ 1 equivalent of 2,3-difluoro-4-methylbenzoic acid (17.2 mmol, 3 g) was dissolved in 101 mL of deionized H_2_O by adding a stoichiometric amount of KOH under magnetic stirring. The solution was then refluxed and 3 equivalents of KMnO_4_ (51.6 mmol, 8.1 g) was added. The reaction mixture was allowed to react for 4 hours. The resulting brown suspension was then cooled down to r.t., filtered over celite, and the cake was washed two times with 20 mL of hot water. The basic solution was then acidified with H_2_SO_4_ to reach pH = 1, leading to the formation of a white solid precipitate, which was recovered through a sintered glass filter, washed with water, and dried under vacuum. Yield: 2.5 g (72%).

**^1^H NMR (400 MHz, DMSO-d6)**: δ 13.88 (s, 2H), 7.72 (s, 2H) ppm.

**^19^F NMR (376 MHz, DMSO-d6)**: δ - 135.78 (s, 2F) ppm.

*Synthesis of 2,5-difluoro terephthalic acid, pF2-H_2_BDC*^10^


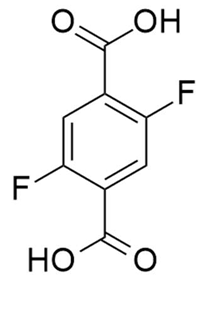


The same oxidation procedure described for *o*F2-H_2_BDC was adopted for the synthesis of *p*F2-H_2_BDC, starting from 1 equivalent of 2,5-difluoro-4-methylbenzoic acid (18 mmol, 3.1 g), a slight excess of KOH (20.8 mmol, 1.17 g) and 3 equivalents of KMnO_4_ (54 mmol, 8.5 g) dissolved in 105 mL of deionized H_2_O.

3.2 g of a white solid was isolated, yield: 88%. Due to insolubility in organic solvents, NMR spectra were recorded in a 0.3 M NaOH solution in D_2_O.

**^1^H NMR (400 MHz, D_2_O)**: δ 7.18 (t, 2H, J = 7.8 Hz) ppm.

**^19^F NMR (376 MHz, D_2_O):** δ -121.52 (t, 2F, J = 7.32 Hz) ppm.

*Synthesis of 2-fluoro terephthalic acid, F1-H_2_BDC*^10^


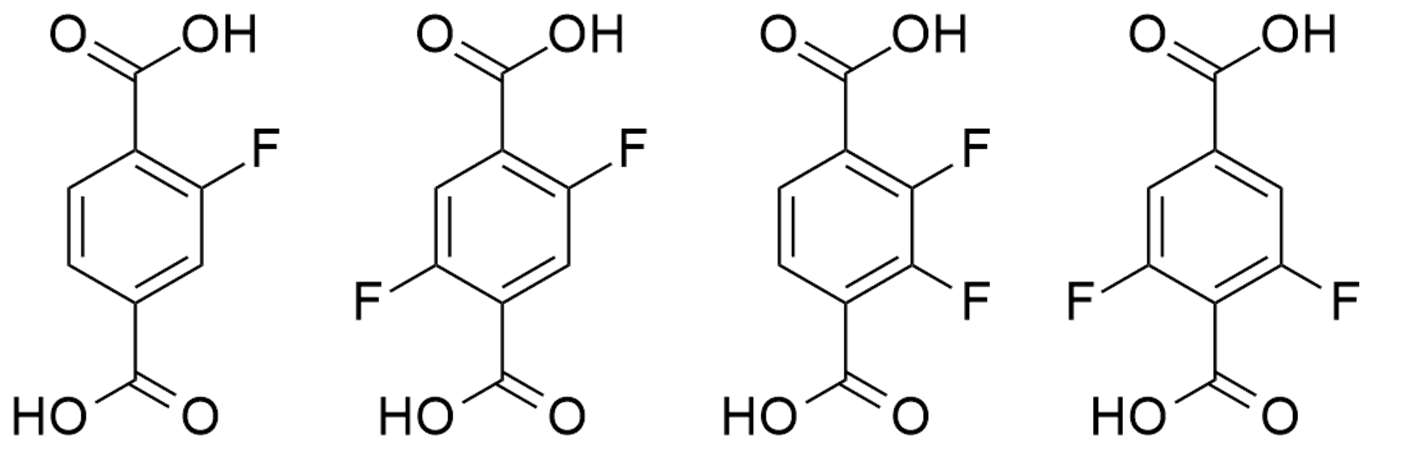


The same oxidation synthesis described for *o*F2-H_2_BDC and *p*F2-H_2_BDC was used to prepare F1-H_2_BDC, starting from 1 equivalent of 2-fluoro-4-methyl benzoic acid (19 mmol, 3 g), 3 equivalents of KMnO_4_ (58 mmol, 9.2 g), 112 mL of H_2_O and KOH (20 mmol, 1.1 g).
2.15 g of a white solid was obtained, yield: 58%.

**^1^H NMR (400 MHz, DMSO-d6):** δ 13.55 (s, 2H), 7.96 (t, J = 7.94 Hz, 1H), 7.83 (m, 1H), 7.73 (dd, J = 11.0 Hz, 1.6 Hz, 1H) ppm.

**^19^F NMR (376 MHz, DMSO-d6)**: δ -110.05 (m, 1F) ppm.


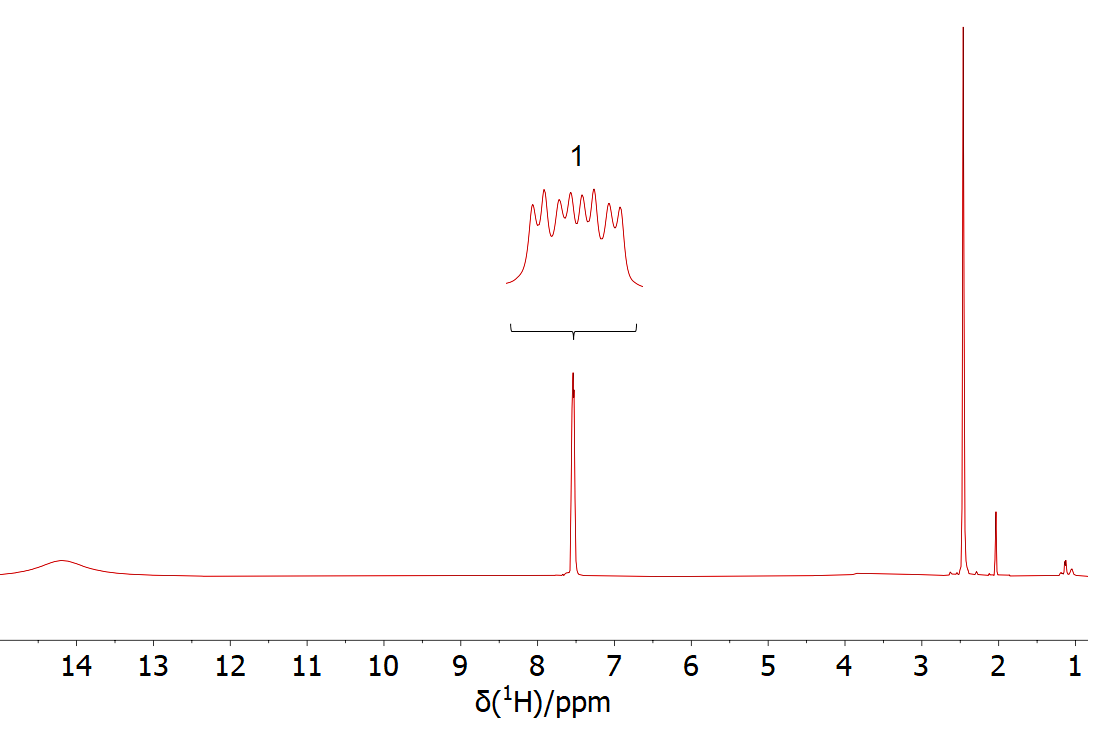


**Figure** **S2.** ^1^H NMR spectrum of F3-H_2_BDC in DMSO-d6. The signal at 2.50 ppm corresponds to DMSO residual peak, while that at 2.07 ppm is due to an acetone impurity.


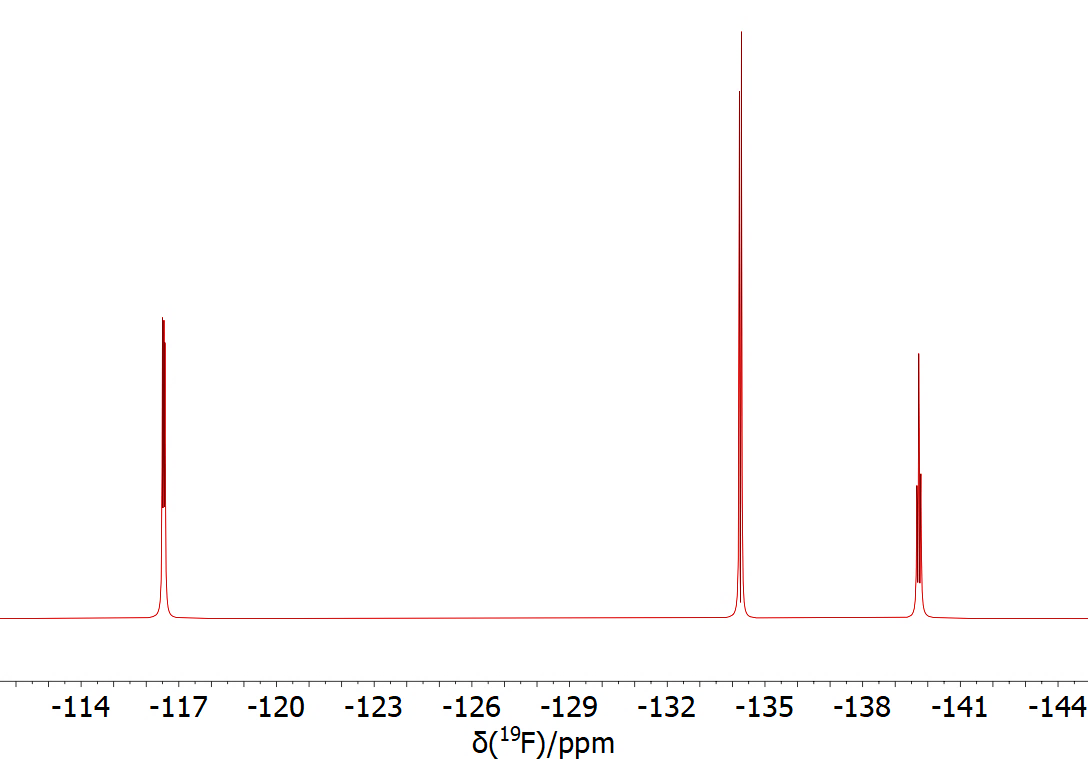


**Figure S3.** ^19^F NMR spectrum of F3-H_2_BDC in DMSO-d6.


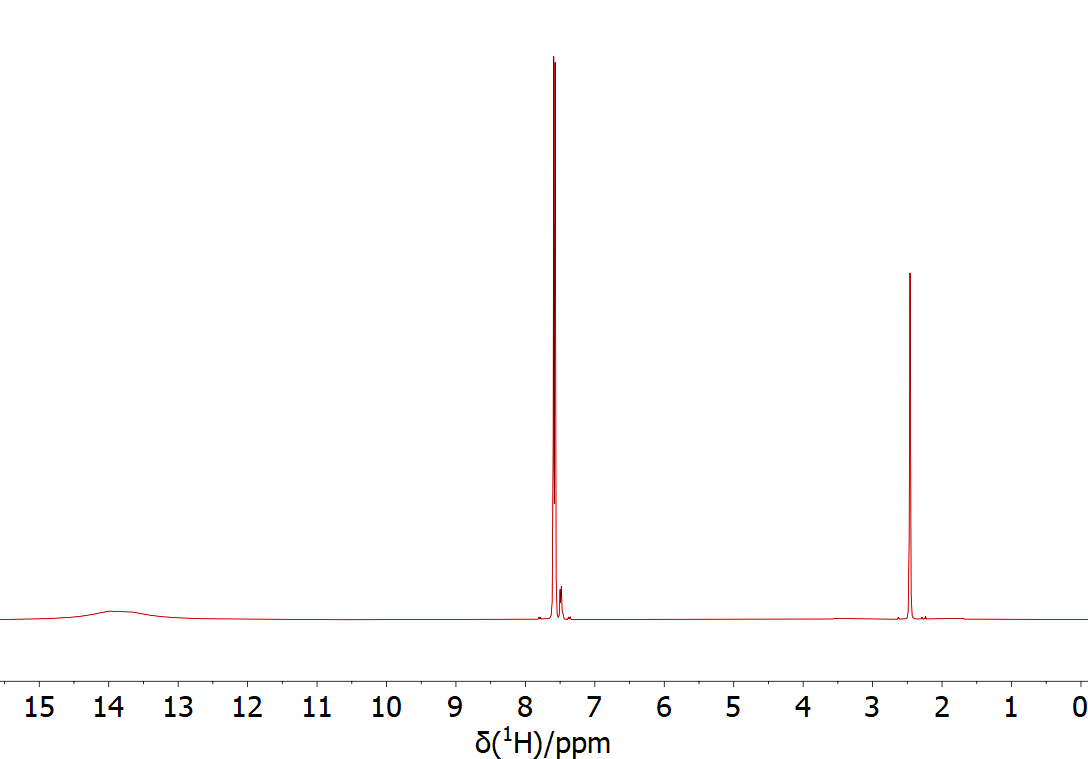


**Figure S4.** ^1^H NMR spectrum of *m*F2-H_2_BDC in DMSO-d6. The signal at 2.50 ppm corresponds to DMSO residual peak.


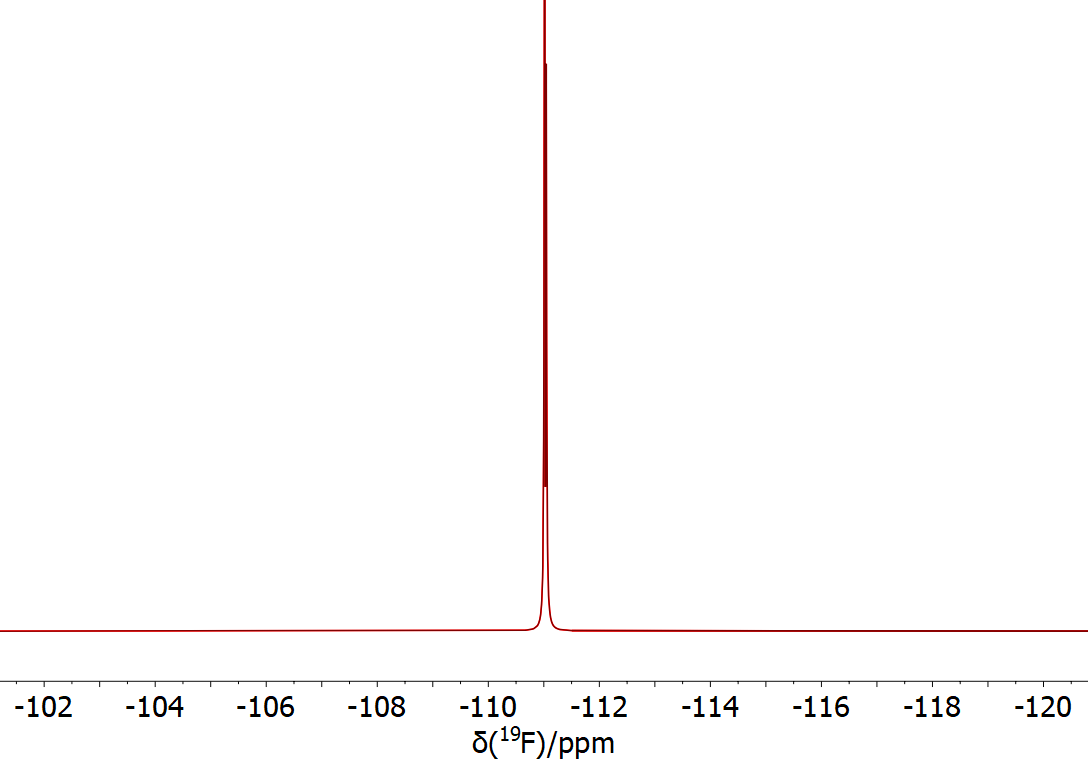


**Figure S5.** ^19^F NMR spectrum of *m*F2-H_2_BDC in DMSO-d6.


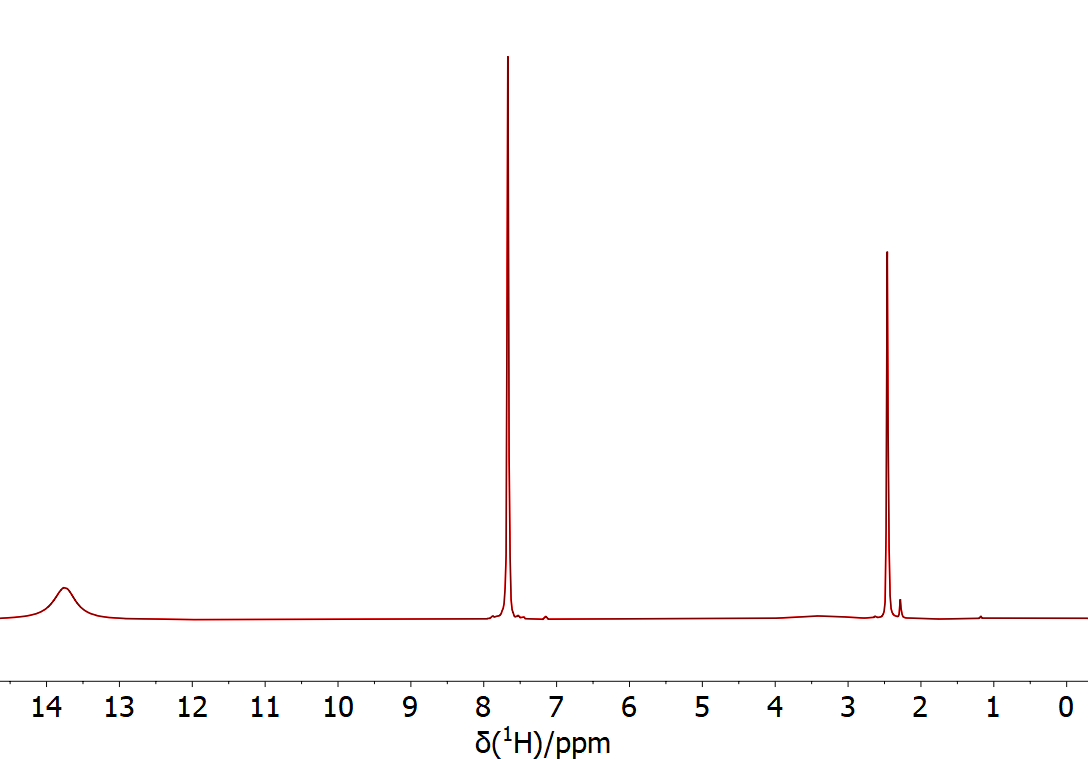


**Figure S6.** ^1^H NMR spectrum of *o*F2-H_2_BDC in DMSO-d6. The signal at 2.50 ppm corresponds to DMSO residual peak.


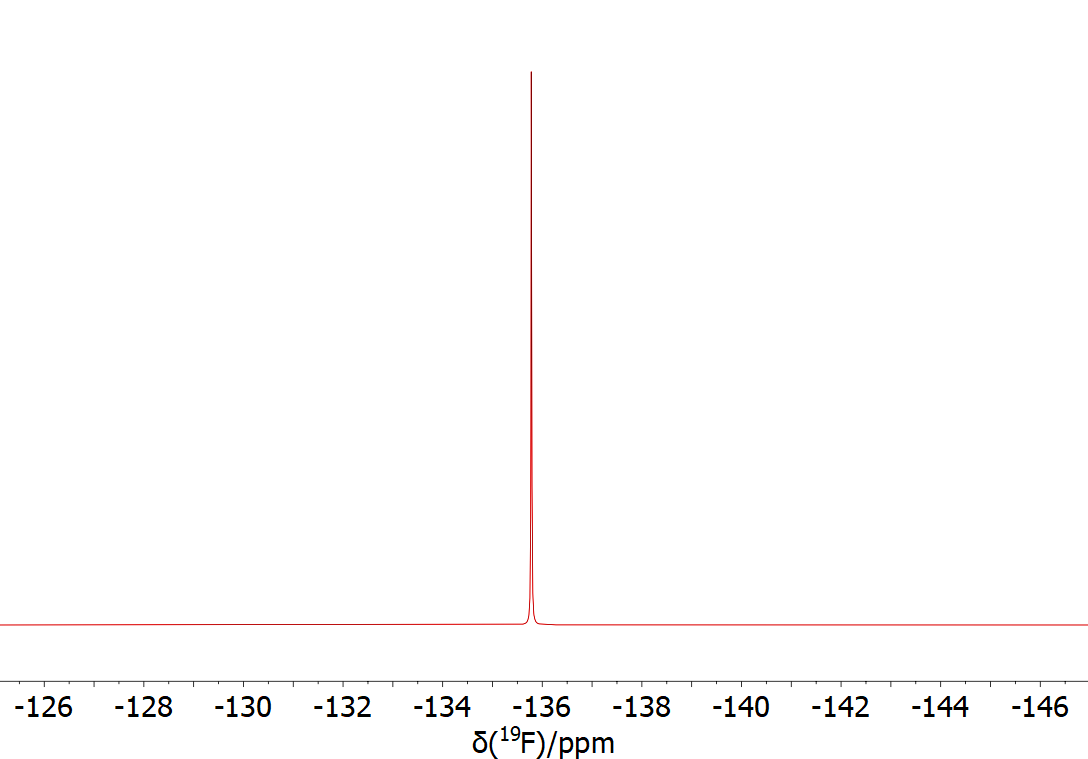


**Figure S7.** ^19^F NMR spectrum of *o*F2-H_2_BDC in DMSO-d6.


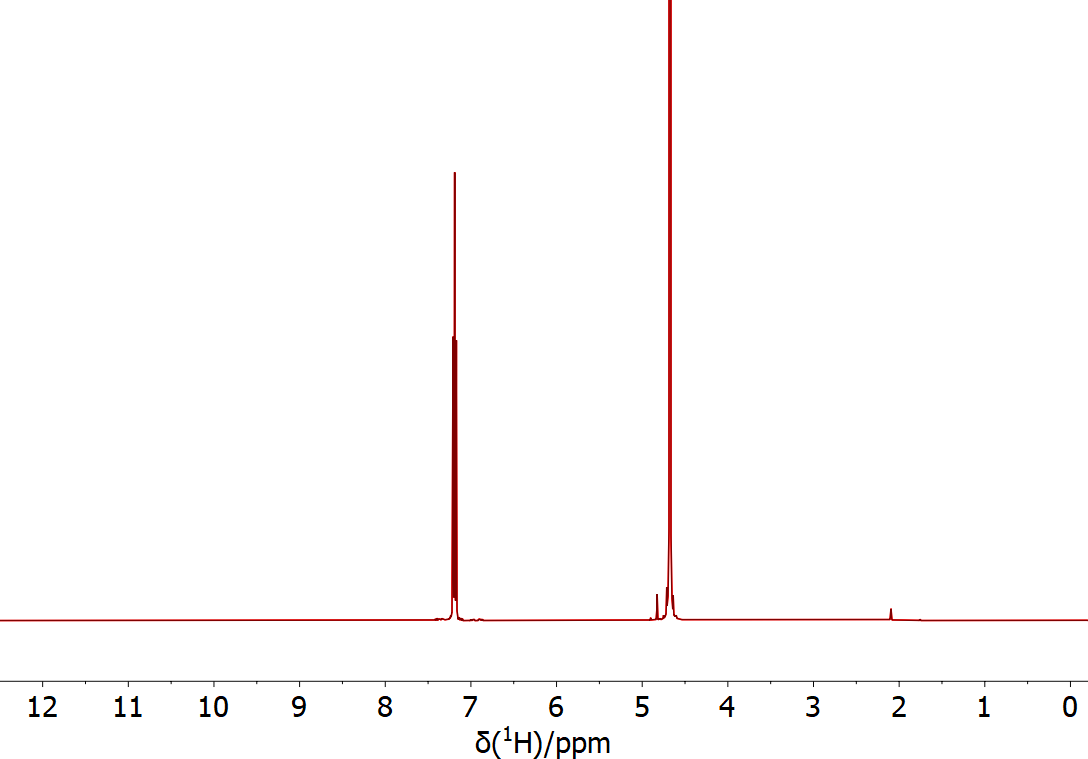


**Figure S8.** ^1^H NMR spectrum of doubly deprotonated *p*F2-H_2_BDC in NaOH 0.3 M in D_2_O. The signal at 4.79 ppm corresponds to H_2_O residual peak.


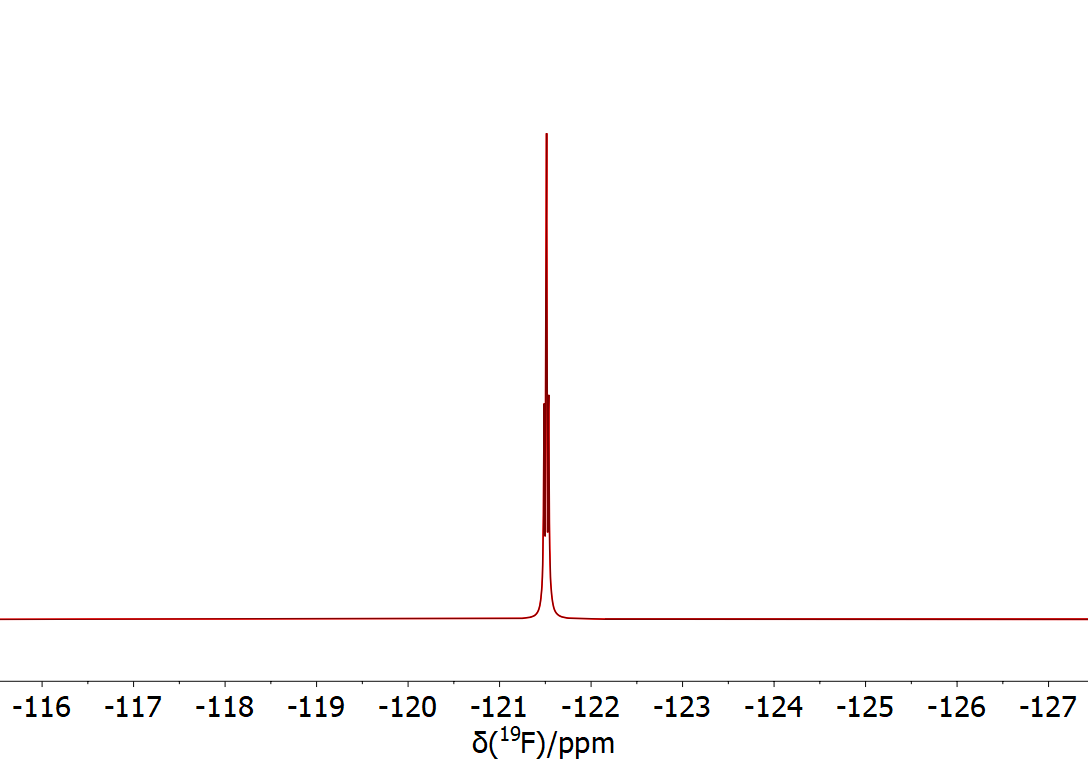


**Figure S9.** ^19^F NMR spectrum of doubly deprotonated *p*F2-H_2_BDC in NaOH 0.3 M in D_2_O.


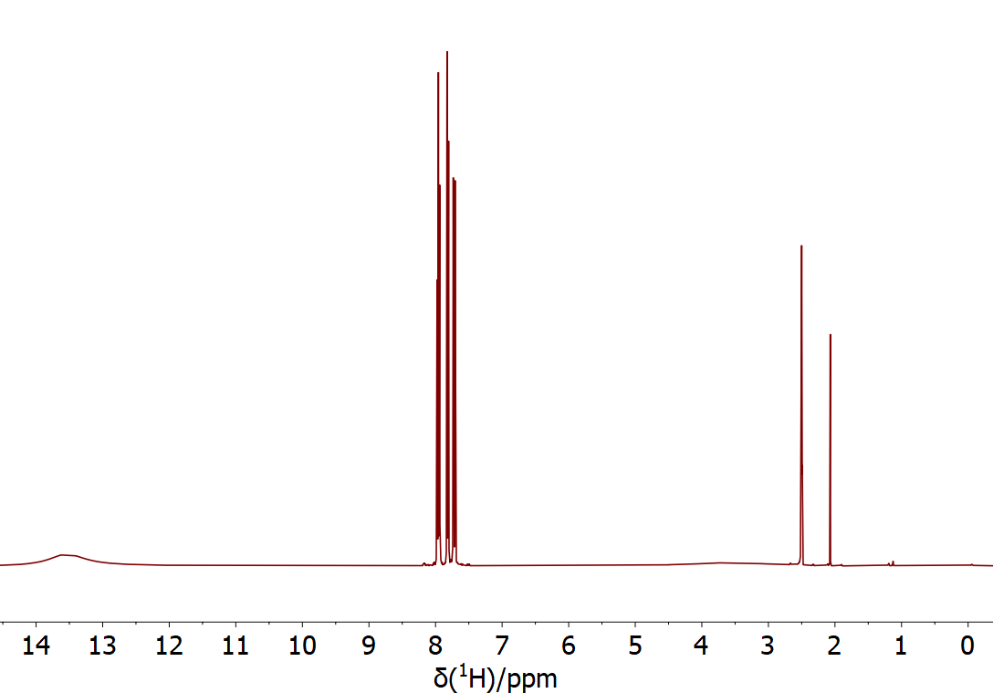


**Figure S10.** ^1^H NMR spectrum of F1-H_2_BDC in DMSO-d6. The signals at 2.50 ppm and 2.07 ppm correspond to DMSO residual peak and acetone impurity, respectively.


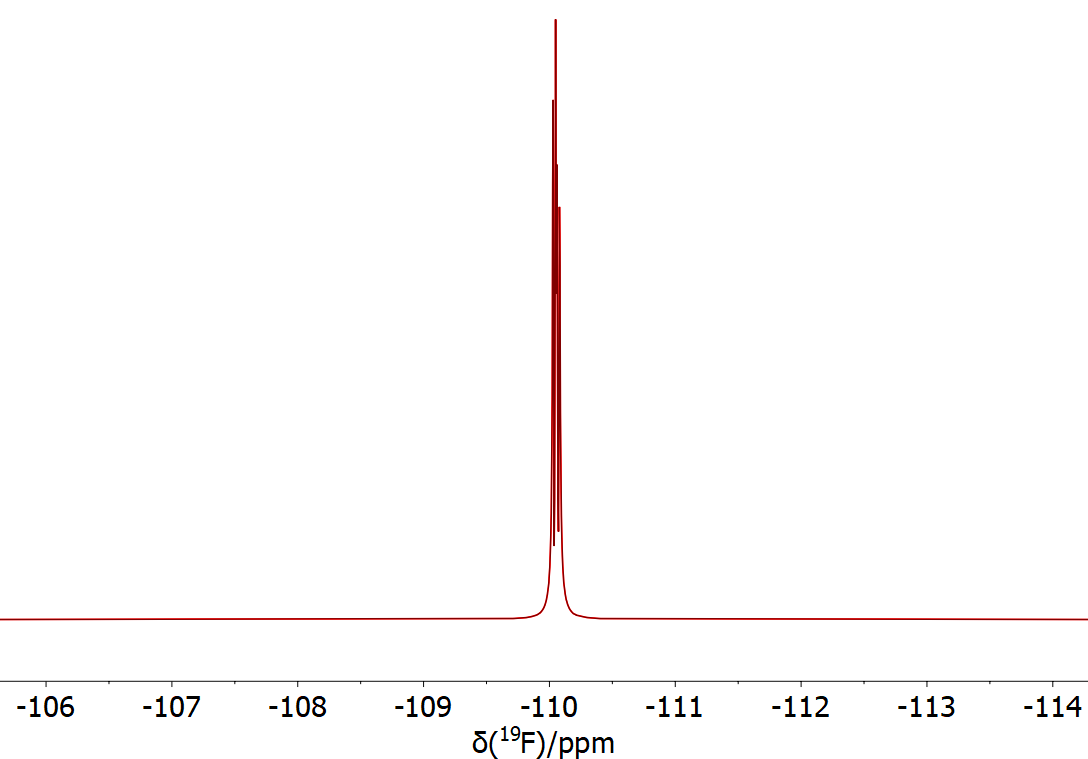


**Figure S11.** ^19^F NMR spectrum of F1-H_2_BDC in DMSO-d6.

**S4. MOFs synthetic screening**

Initial attempts to obtain MIL-140A analogues using F*x*-H_2_BDC linkers were made by adapting the optimised synthesis conditions reported for both F4_MIL-140A(Ce) (water-based synthesis)^11^ and MIL-140(Ce) frameworks based on non-fluorinated linkers (solvothermal acetonitrile-based synthesis).^12^ Both these methods proved unsuitable for the attainment of phase pure and/or porous products.

A novel synthetic strategy based on a MeOH:H_2_O (80:20 vol:vol) mixed solvent was therefore developed. The synthetic conditions (MeOH:H_2_O ratio, reaction time and temperature) were systematically investigated using F3-H_2_BDC as linker and the obtained materials were studied by PXRD analysis to determine the efficiency of the protocol in terms of phase identity, phase purity and crystallite size (as indicated by the broadening of the diffraction profile). By varying the MeOH content from 60% to 90% in volume and working at 60 °C, pure MIL-140A phases were obtained (**Figure S12**). By increasing the MeOH:H_2_O ratio, crystallinity increased while the yield decreased after one hour of reaction (**Table S1** and **Figure S13**). By increasing the reaction temperature the yield decreased, probably because the reduction of Ce^IV^ to Ce^III^ became competitive.

**Table S1.** Syntheses conducted in MeOH:H_2_O mixtures using F3-H_2_BDC as a test bed.

| **CAN**  **(mmol)** | **F3-H_2_BDC**  **(mmol)** | **MeOH:H_2_O ratio (vol:vol)** | **Final Volume**  **(mL)** | **Temperature**  **(°C)** | **Phase** | **Reaction time** | **Yield** |
| --- | --- | --- | --- | --- | --- | --- | --- |
| 0.375 | 0.375 | 90:10 | 7.5 | 60 | MIL-140A | 1 h | 13% |
| 0.375 | 0.375 | 80:20 | 7.5 | 60 | MIL-140A | 1 h | 40% |
| 0.375 | 0.375 | 70:30 | 7.5 | 60 | MIL-140A | 1 h | 48% |
| 0.375 | 0.375 | 60:40 | 7.5 | 60 | MIL-140A | 1 h | 35% |
| 0.375 | 0.375 | 80:20 | 7.5 | 70 | MIL-140A | 45 min | 14% |
| 0.375 | 0.375 | 80:20 | 7.5 | 70 | MIL-140A | 25 min | 25% |





**Figure S12.** PXRD patterns of F3_MIL-140A(Ce) MOFs obtained at at 60 °C with 1 h reaction time and different MeOH:H_2_O ratios.


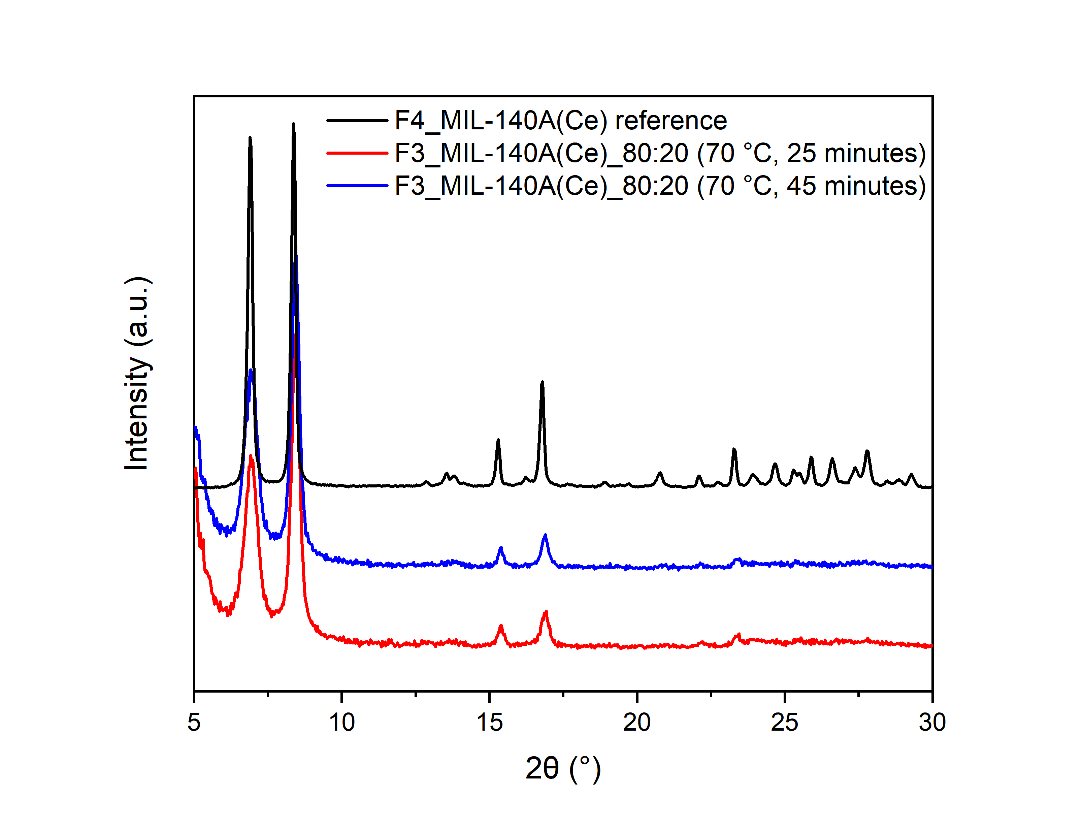


**Figure S13.** PXRD patterns of F3_MIL-140A(Ce) MOFs obtained at 70 °C with different reaction times at 80:20 MeOH:H_2_O ratio.

The obtained results highlight that a good compromise in terms of yield and crystallinity is achieved when the reaction is conducted with the MeOH:H_2_O 80:20 vol:vol mixture at 60 °C for one hour. These synthetic conditions were transferred to the preparation of MOFs with *p*F2-H_2_BDC, *o*F2-H_2_BDC, *m*F2-H_2_BDC, and F1-H_2_BDC linkers. A pure MIL-140A phase was obtained only with *p*F2-H_2_BDC (**Figure 2**), whereas UiO-66 phases were found with F1-H_2_BDC and *o*F2-H_2_BDC (**Figure S14**), and a negligible amount of a poorly crystalline solid was synthesised with *m*F2-H_2_BDC (**Table S2**).

**Table S2.** Summarised synthetic conditions of F*x*_MIL-140A(Ce). In all cases the MeOH:H_2_O ratio is 80:20 vol:vol.

| **Linker** | **CAN**  **(mmol)** | **Linker**  **(mmol)** | **Final Volume**  **(mL)** | **Temperature**  **(°C)** | **Phase** | **Reaction time** | **Yield** |
| --- | --- | --- | --- | --- | --- | --- | --- |
| F1-H_2_BDC | 0.75 | 0.75 | 15 | 60 | UiO-66 | 1 h | - |
| *p*F2-H_2_BDC | 0.75 | 0.75 | 15 | 60 | MIL-140A | 1 h | 55% |
| *o*F2-H_2_BDC | 0.75 | 0.75 | 15 | 60 | UiO-66 | 1 h | - |
| *m*F2-H_2_BDC | 0.75 | 0.75 | 15 | 60 | Undefined | 1 h | - |
| F3-H_2_BDC | 0.75 | 0.75 | 15 | 60 | MIL-140A | 1 h | 40% |


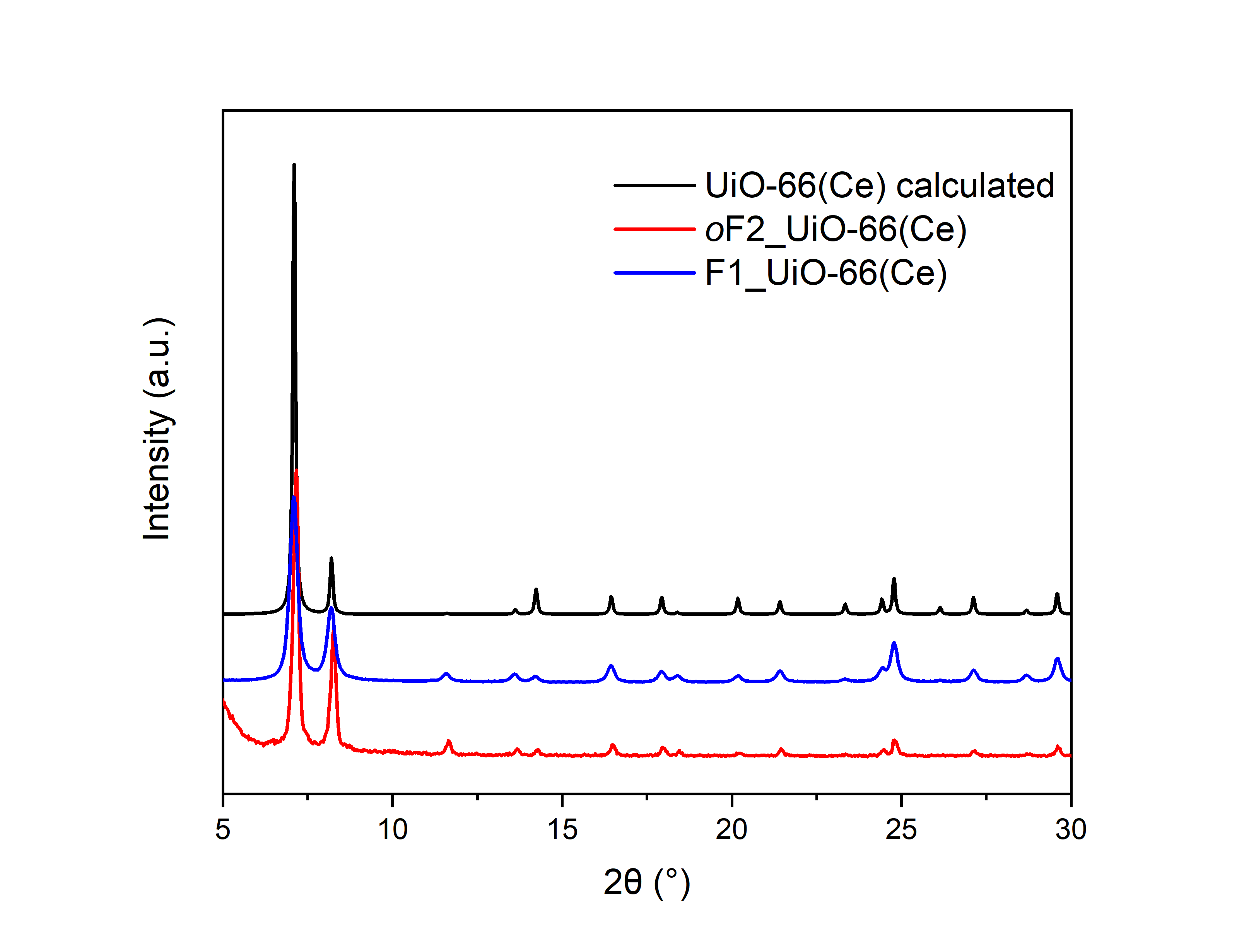


**Figure S14.** PXRD patterns of *o*F2_UiO-66(Ce) and F1_UiO-66(Ce) obtained with the MeOH:H_2_O based synthesis at 60 °C.

The same synthetic method employed for F3_MIL-140A(Ce) and *p*F2_MIL-140A(Ce) was applied to prepare F4_MIL-140A(Ce)_MeOH:H_2_O, which was used to assess the impact of the synthetic conditions on the adsorption properties of the perfluorinated parent material.

**Table S3.** Synthesis conditions of F4_MIL-140A(Ce)_MeOH:H_2_O in MeOH:H_2_O 80:20 vol:vol.

| **Linker** | **CAN**  **(mmol)** | **Linker**  **(mmol)** | **Final Volume**  **(mL)** | **Temperature**  **(°C)** | **Phase** | **Reaction time** | **Yield** |
| --- | --- | --- | --- | --- | --- | --- | --- |
| F4-H_2_BDC | 0.75 | 0.75 | 15 | 60 | MIL-140A | 1 h | 68 % |

Both the PXRD pattern (**Figure S15**) and ATR-IR spectrum (**Figure S16**) of F4_MIL-140A(Ce)_MeOH:H₂O align well with those of the F4_MIL-140A(Ce) reference. On the other hand, F4_MIL-140A(Ce)_MeOH:H₂O differs from the reference in the morphology and size of the crystallites as it exhibits ill-defined morphology with particles significantly smaller (**Figure S17**).


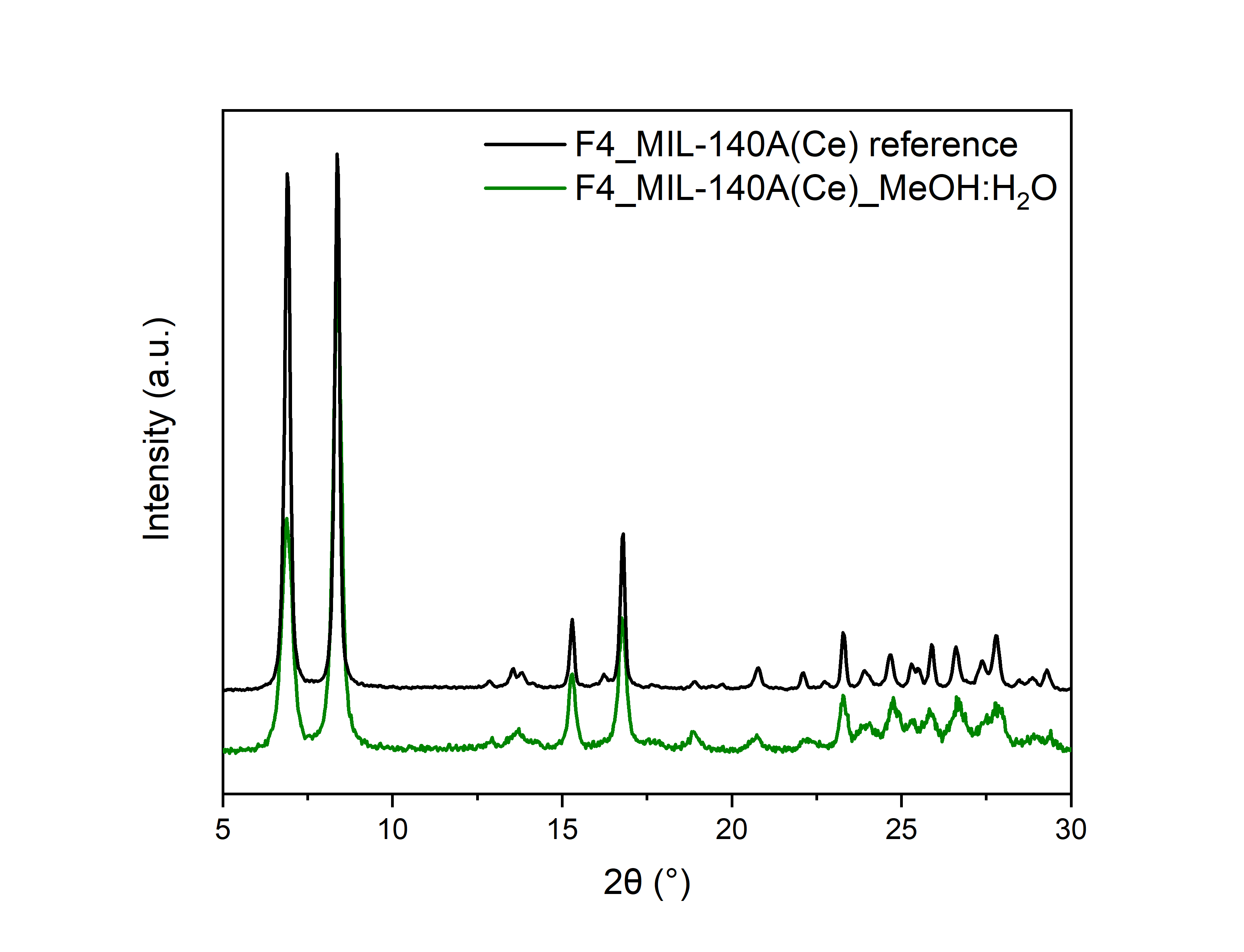


**Figure S15.** PXRD patterns of F4_MIL-140A(Ce) reference (black) and F4_MIL-140A(Ce)_MeOH:H_2_O (green).


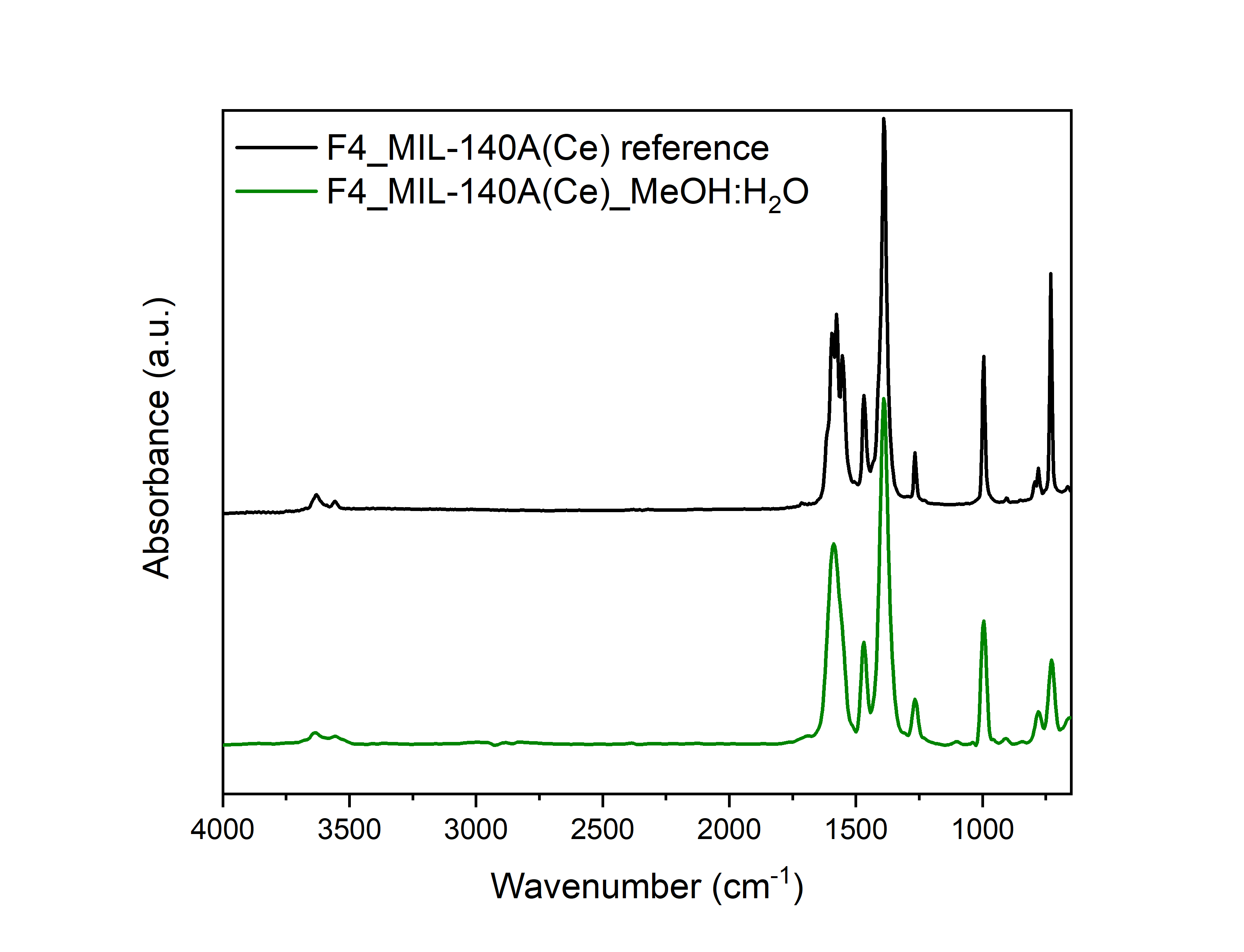


**Figure S16.** ATR-IR spectra of F4_MIL-140A(Ce) reference (black) and F4_MIL-140A(Ce)_MeOH:H_2_O (green).


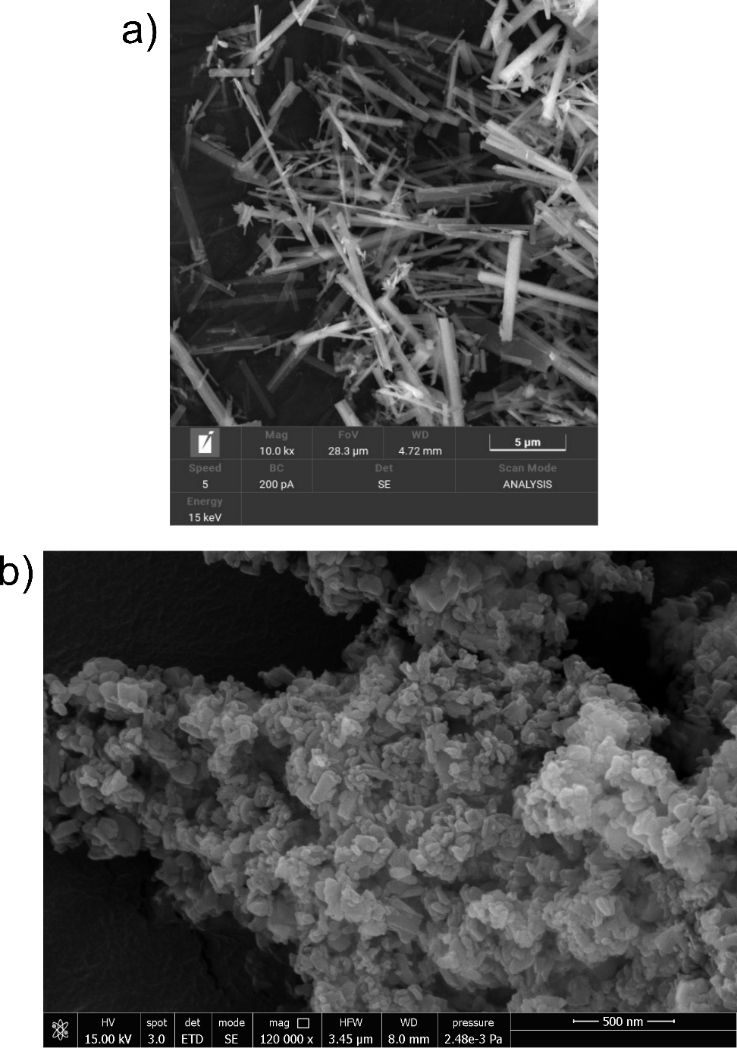


**Figure S17.** SEM images of a) F4_MIL-140A(Ce) reference and b) F4_MIL-140A(Ce)_MeOH:H_2_O.

**S5. Preliminary characterisation of F*x*_MIL-140A(Ce)**

*Attenuated total reflectance infrared (ATR-IR) spectroscopy*


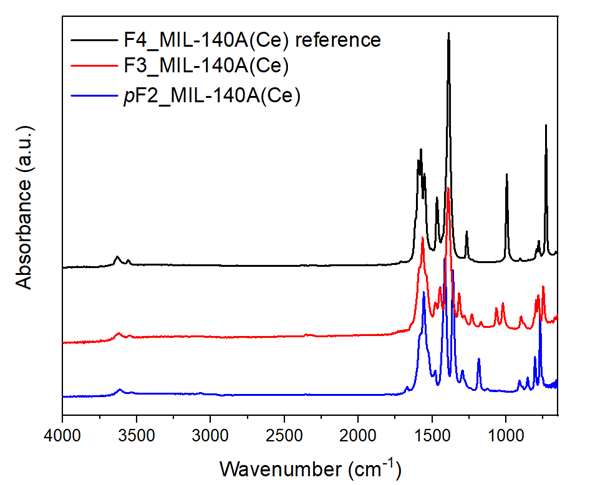


**Figure S18.** ATR-IR spectra of F4_MIL-140A(Ce) reference (black), F3_MIL-140A(Ce) (red) and *p*F2_MIL-140A(Ce) (blue).

*Thermogravimetric Analyses (TGA)*


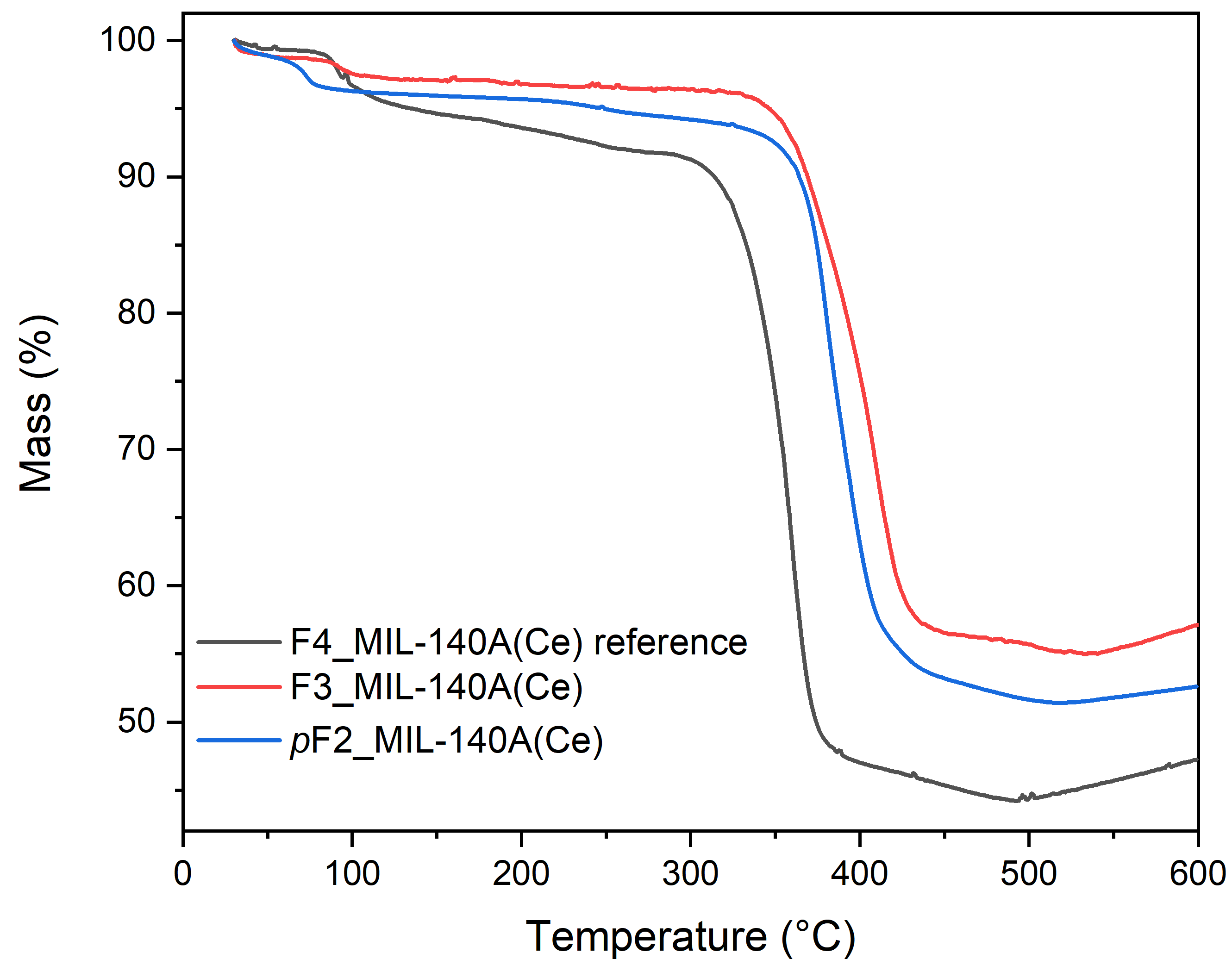


**Figure S19.** TG curves of F4_MIL-140A(Ce) reference (black), F3_MIL-140A(Ce) (red) and *p*F2_MIL-140A(Ce) (blue).

*p*F2_MIL-140A(Ce) displays a well-defined mass loss in the temperature range 30–100 °C, associated to the loss of adsorbed solvent molecules, aligning well with the trend observed in the reference. In the same temperature range, F3_MIL-140A(Ce) displays a less defined mass loss. Interestingly, the decomposition temperature at which the organic part of the framework of F4_MIL-140A(Ce) reference starts degrading (which is the temperature corresponding to the vertical mass loss step) is lower than that observed for the less fluorinated MOFs, suggesting an influence of the fluorination degree of the linker on the thermal stability of the framework. In all cases—and particularly for the F4_MIL-140A(Ce) reference—a slight increase in mass is observed at elevated temperatures (>500 °C). To further investigate this behaviour, variable-temperature PXRD (VT-PXRD) measurements were carried out on F4_MIL-140A(Ce). The data collected at 400 °C (**Figure S20**) clearly reveal the formation of CeF₃ as a thermal decomposition product, indicating that a reduction process occurs during heating. To gain a more comprehensive understanding of this transformation, additional samples were calcined in a furnace at 500 °C and subsequently analysed (**Figure S21**). In this case, CeO₂ was identified as the major product, accompanied by minor impurities of CeF₃. Although the operating conditions of the VT-PXRD experiments and the calcination process are not directly comparable to those used in the TGA, the combined results suggest the migration of F from the linker to the metal and the occurrence of redox processes during thermal treatment, the nature of which is difficult to interpret but likely accounts for the unusual mass trend observed at high temperatures in the TG curves shown in **Figure S19**.


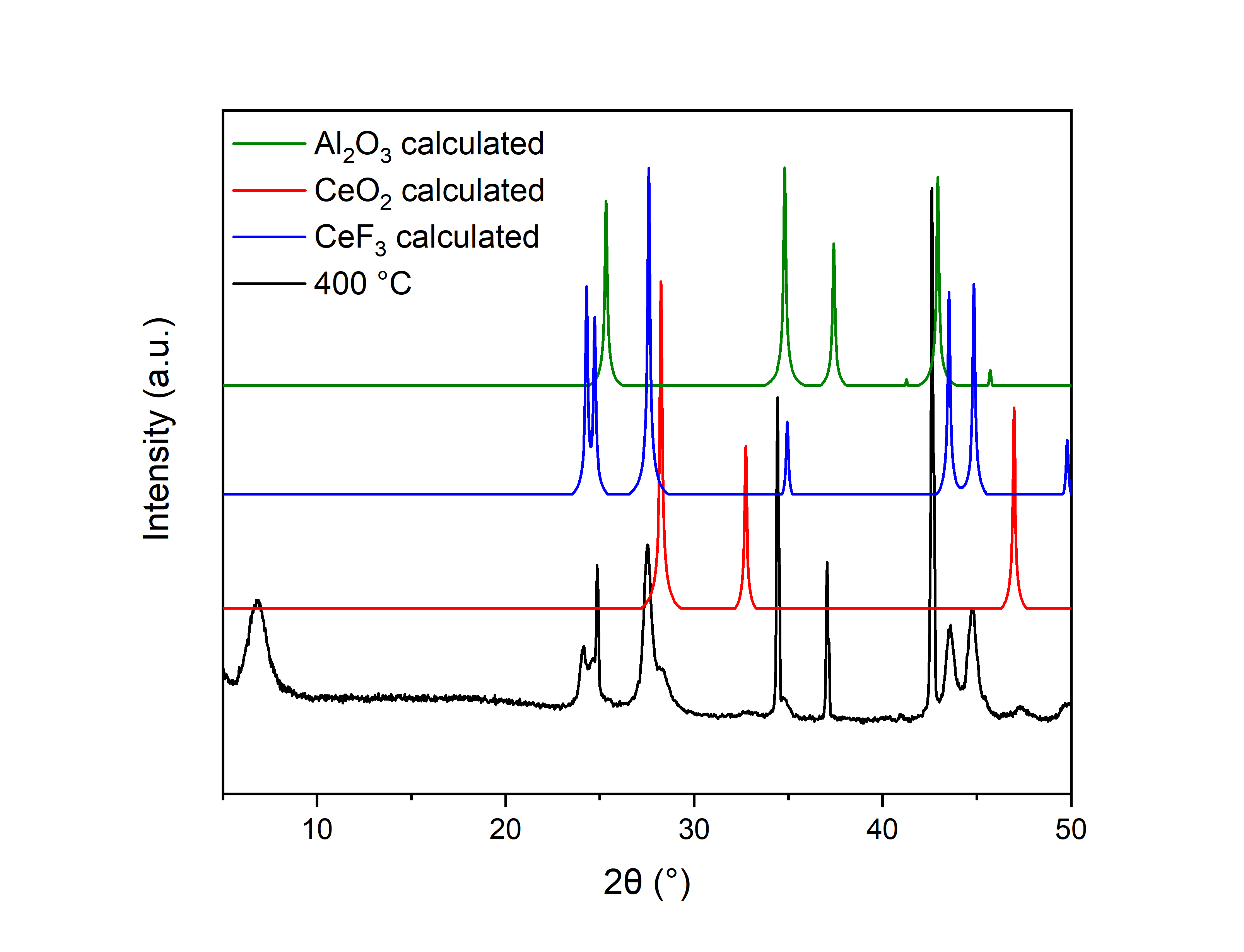


**Figure S20.** PXRD pattern of F4_MIL-140A(Ce) obtained by heating the sample at 400 °C in the BTS chamber (black) compared to the calculated patterns of CeO_2_ (red), CeF_3_ (blue) and Al_2_O_3_ (green). The presence of Al₂O₃ in the black pattern is due to shrinkage of the powder upon decomposition, which exposes the underlying Al₂O₃ from the sample holder.


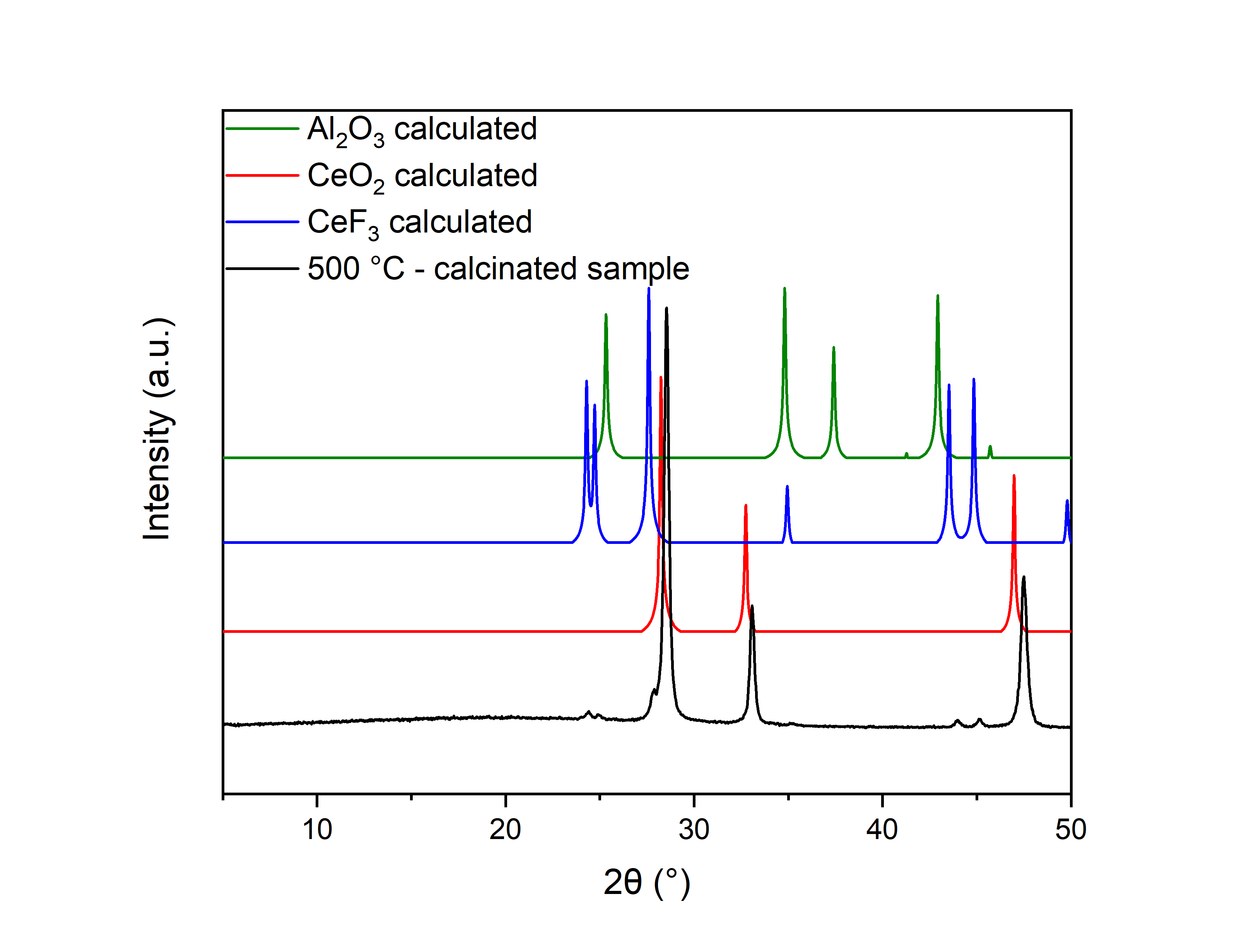


**Figure S21.** PXRD pattern of F4_MIL-140A(Ce) calcined at 500 °C in the oven (black) compared to the calculated patterns of CeO_2_ (red), CeF_3_ (blue) and Al_2_O_3_ (green).

**Table S4.** Theoretical and experimental mass loss of F*x*_MIL-140A(Ce) samples and F4_MIL-140A(Ce) reference.

| **Compound** | **Theoretical Formula^a^** | **Molecular weight**  **(g/mol)** | **Theoretical mass loss (%)^b^** | **Experimental mass loss (%)** |
| --- | --- | --- | --- | --- |
| F4_MIL-140A(Ce) reference | CeO(F4-BDC) | 391.87 | 56 | 50 |
| F3_MIL-140A(Ce) | CeO(F3-BDC) | 373.88 | 54 | 43 |
| *p*F2_MIL-140A(Ce) | CeO(F2-BDC) | 355.89 | 52 | 44 |

^a^ Of the activated MOFs

^b^ Assuming CeO_2_ as the final decomposition product at 600 °C

*Scanning Electron Microscopy (SEM)*


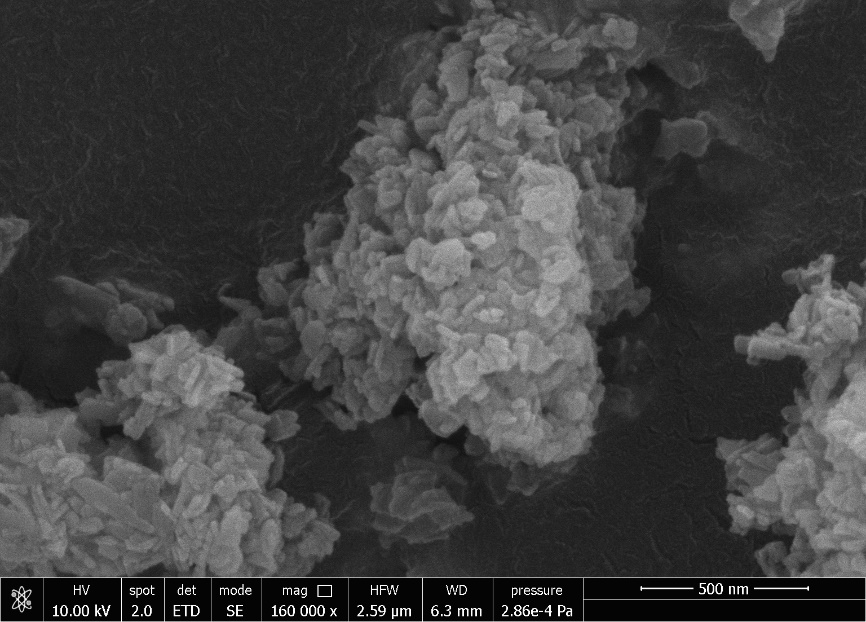


**Figure S22.** SEM image of F3_MIL-140A(Ce).


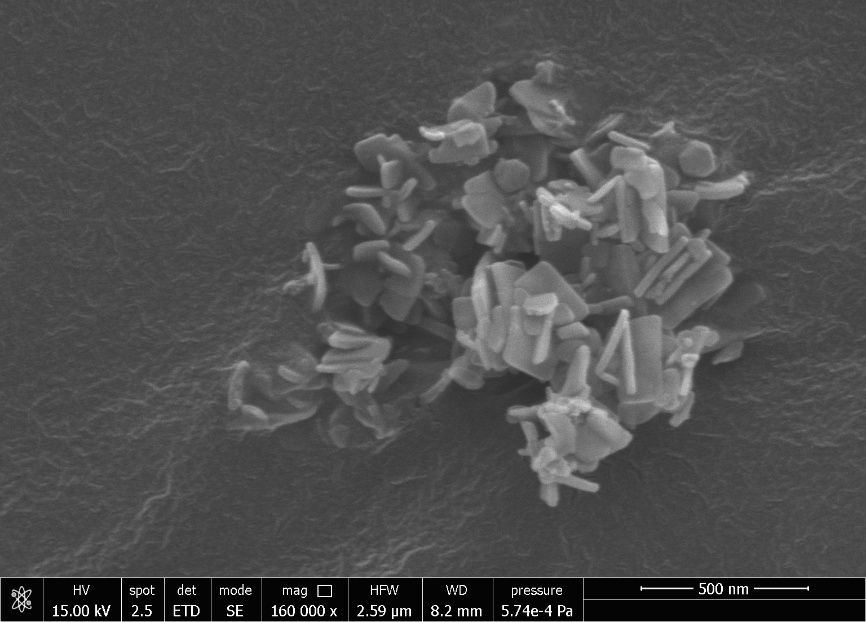


**Figure S23.** SEM image of *p*F2_MIL-140A(Ce).

*Solid state NMR spectroscopy*

In the aromatic spectral region of ^1^H DE MAS spectra (**Figure S24**), two partially superimposed signals are observed for both F3_MIL-140A(Ce) (7.8 and 6.9 ppm) and *p*F2_MIL-140A(Ce) (8.1 and 7.1 ppm). The spectra also show a very weak signal at about 14.5 ppm (carboxylic acid region), indicating the presence of a negligible amount of unreacted linker trapped in the MOF in both cases. Thus, we can state that the two signals in the aromatic region originate from protons belonging to the two crystallographically inequivalent linkers.^8^ ^19^F DE MAS spectra (**Figure S24**) show the isotropic signals of the fluorine atoms of F3_MIL-140A(Ce) at −115.2 ppm, −131.8 ppm, and −141.8 ppm, and that of *p*F2_MIL-140A(Ce) at −118.0 ppm. ^1^H^_13^C and ^19^F^_13^C CP MAS spectra of F3_MIL-140A(Ce) and *p*F2_MIL-140A(Ce) are shown and assigned to the linker carbons in **Figure S25**.


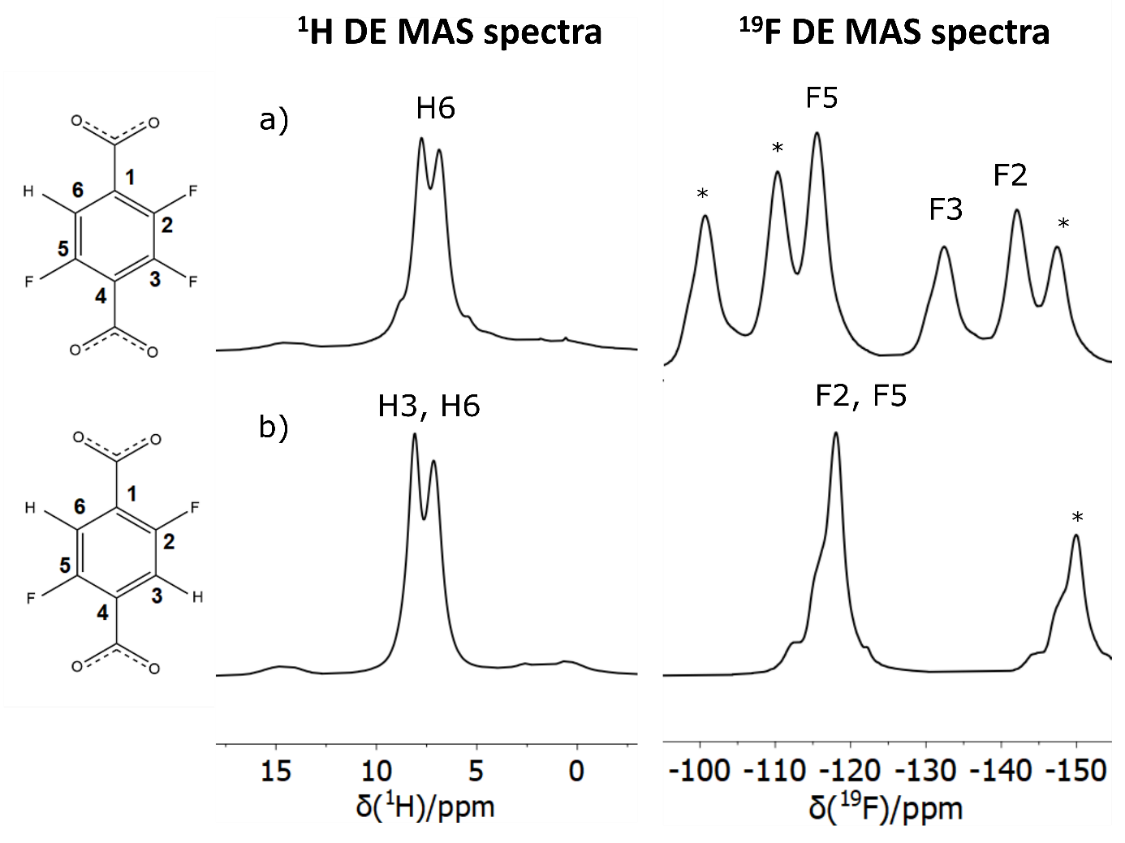


**Figure S24**. ^1^H DE MAS (left) and ^19^F DE MAS NMR spectra (right) of F3_MIL-140A(Ce) (a) and *p*F2_MIL-140A(Ce) (b). Spinning sidebands are marked with an asterisk.


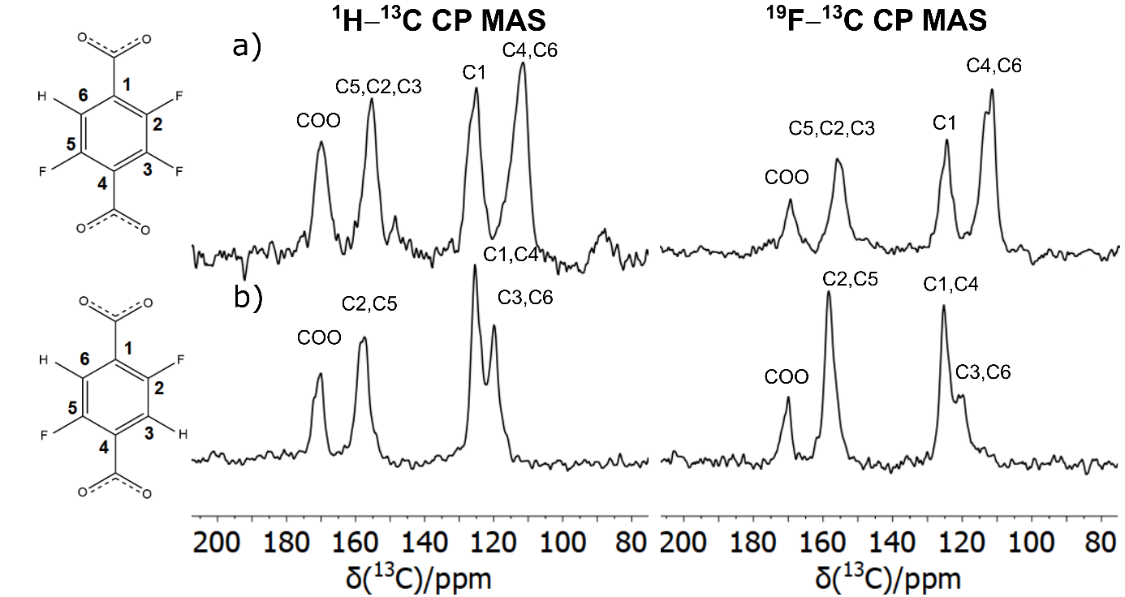


**Figure S25.** ^1^H-^13^C CP MAS and ^19^F-^13^C CP MAS spectra of a) F3_MIL-140A(Ce) and b) *p*F2_MIL-140A(Ce).

*Liquid-state NMR spectroscopy*

Prior to NMR analysis, 10-20 mg of each sample was activated in an oven at 120 °C for at least 2 hours and then hydrolysed in 1 mL of 1 M of NaOH solution in D_2_O overnight. Owing to solubility issues, the hydrolysis of as-*p*F2_MIL-140A(Ce) was carried out with 1 mL of 0.2 M NaOH solution in D_2_O. In all cases, 0.1 M 2,6-difluorobenzoic acid (DFBA) was used as the internal standard for absolute concentration determination.


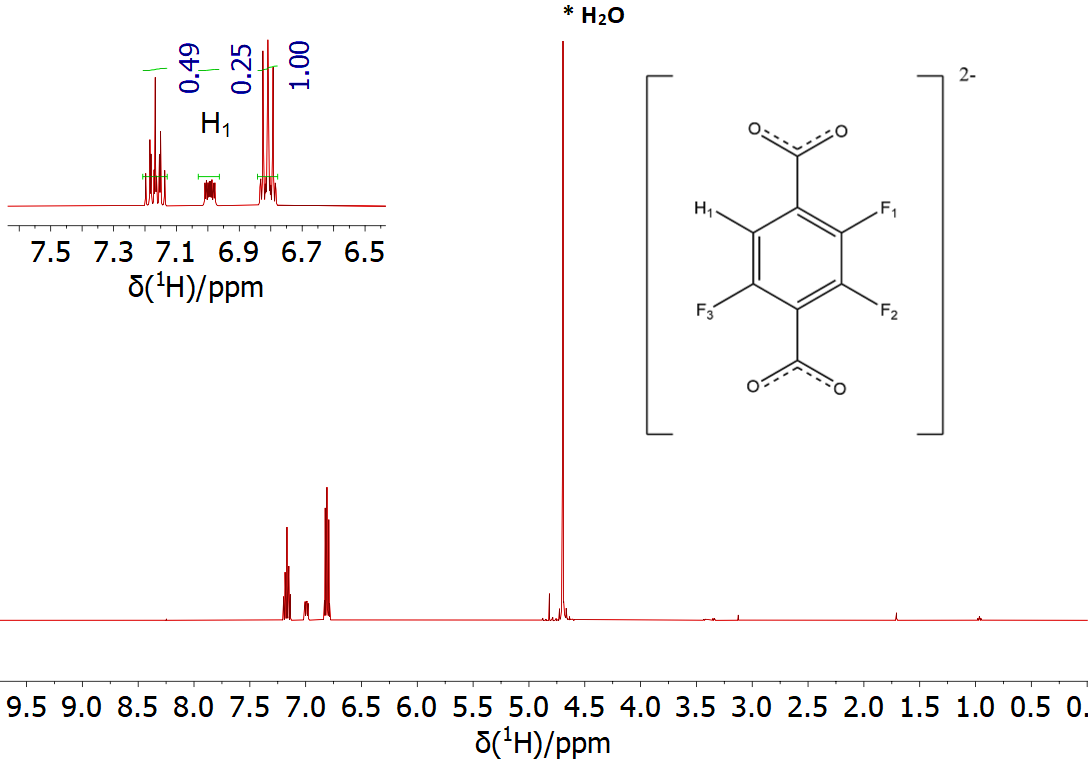


**Figure S26.** ^1^H NMR spectrum of digested F3_MIL-140A(Ce) (0.0194 g). DFBA exhibits two signals in the NMR spectrum: one at 7.2 ppm (corresponding to one proton) and another at 6.8 ppm (corresponding to two protons). The proton signal of the F3-BDC linker (labeled as H_1_) appears at 7.0 ppm. To determine the moles of deprotonated linker, the integral of H_1_ (0.24) was divided by the integral of the DFBA proton (0.5). This ratio was then multiplied by the concentration of the DFBA standard (0.1 M) and the volume of the solution (1 mL), resulting in a quantity of 4.8×10^-5^ mol of deprotonated linker. The wt% of the linker in the activated MOF was calculated by multiplying this amount (4.8×10^-5^ mol) by the molecular weight of the deprotonated linker (218 g mol^-1^), and then dividing by the weight of the activated MOF sample (0.0175 g). The wt% of linker was determined to be 58%.


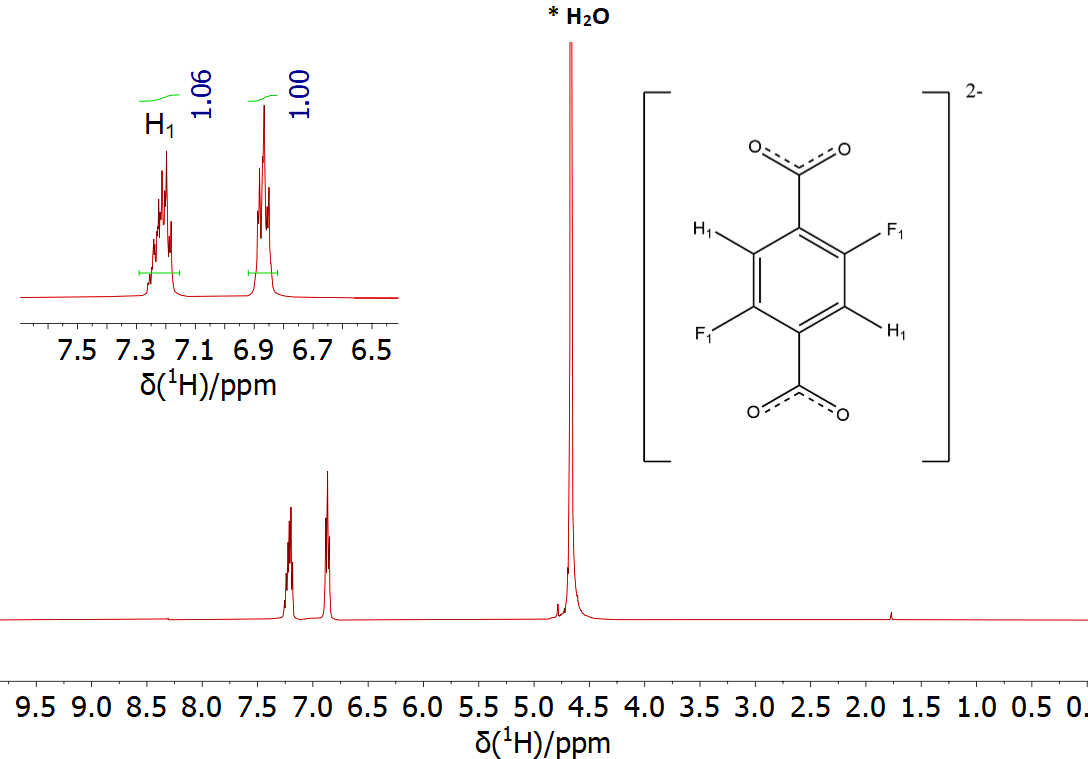


**Figure S27.** ^1^H NMR spectrum of digested *p*F2_MIL-140A(Ce) (0.0197 g). The wt% of linker was determined according to the calculation procedure described for F3_MIL-140A(Ce), resulting in 57%.

**Table S5.** Theoretical and experimental chemical formula of *p*F2_MIL-140A(Ce) and F3_MIL-140A(Ce) based on quantitative liquid-state NMR analysis.

| **MOF** | ***p*F2_MIL-140A(Ce)** | **F3_MIL-140A(Ce)** |
| --- | --- | --- |
| **Theoretical**  **formula^a^** | CeO(*p*F2-BDC) | CeO(F3-BDC) |
| **Theoretical**  **linker mass** | 56 % | 58 % |
| **Calculated**  **linker mass** | 57 % | 57 % |
| **Calculated**  **Formula** | CeO(*p*F2-BDC) | CeO(F3-BDC) |

^a^ Of the activated MOFs

*Adsorption properties*


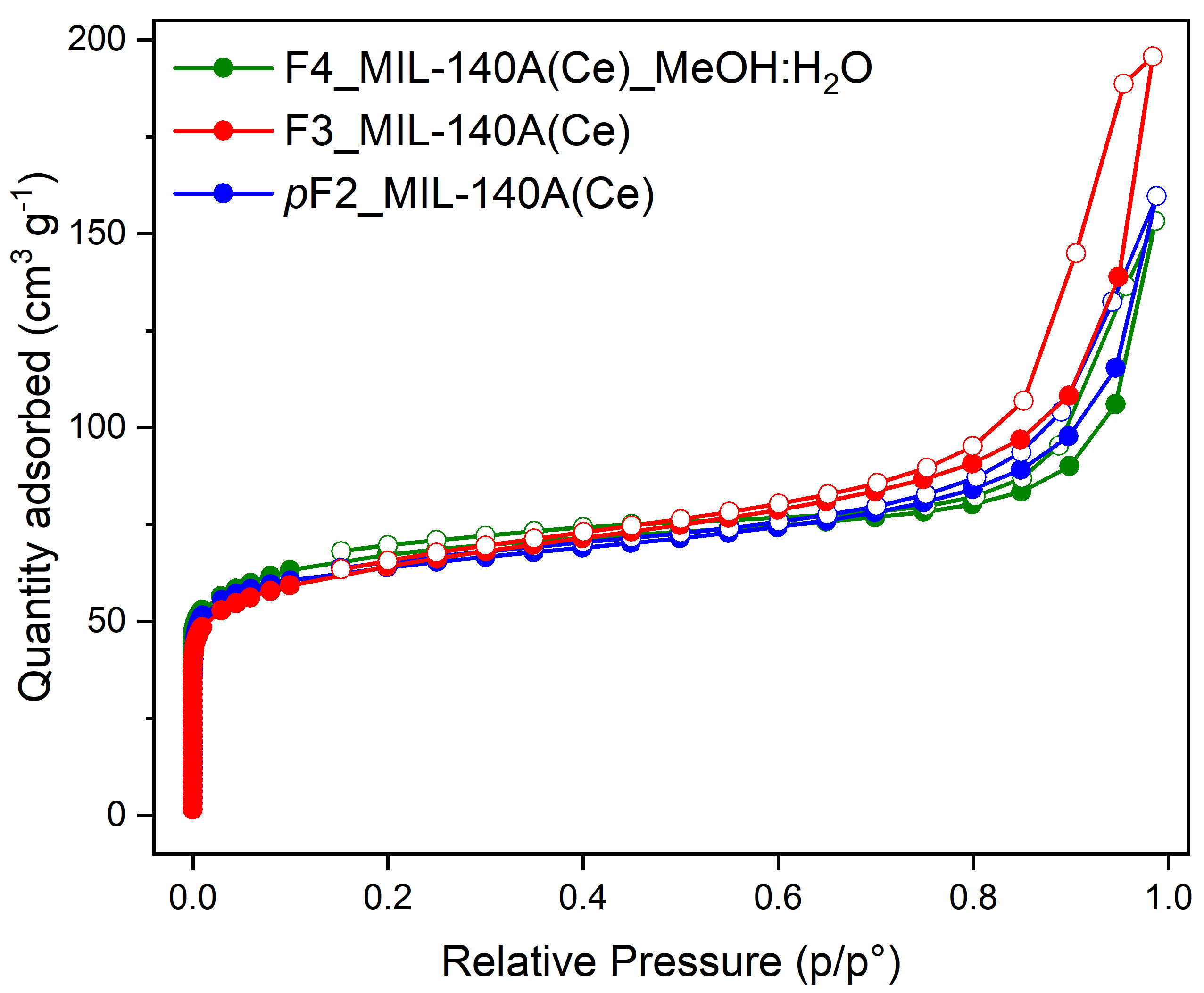


**Figure S28.** Ar adsorption/desorption isotherms of F*x*_MIL-140A(Ce) collected at –186 °C.


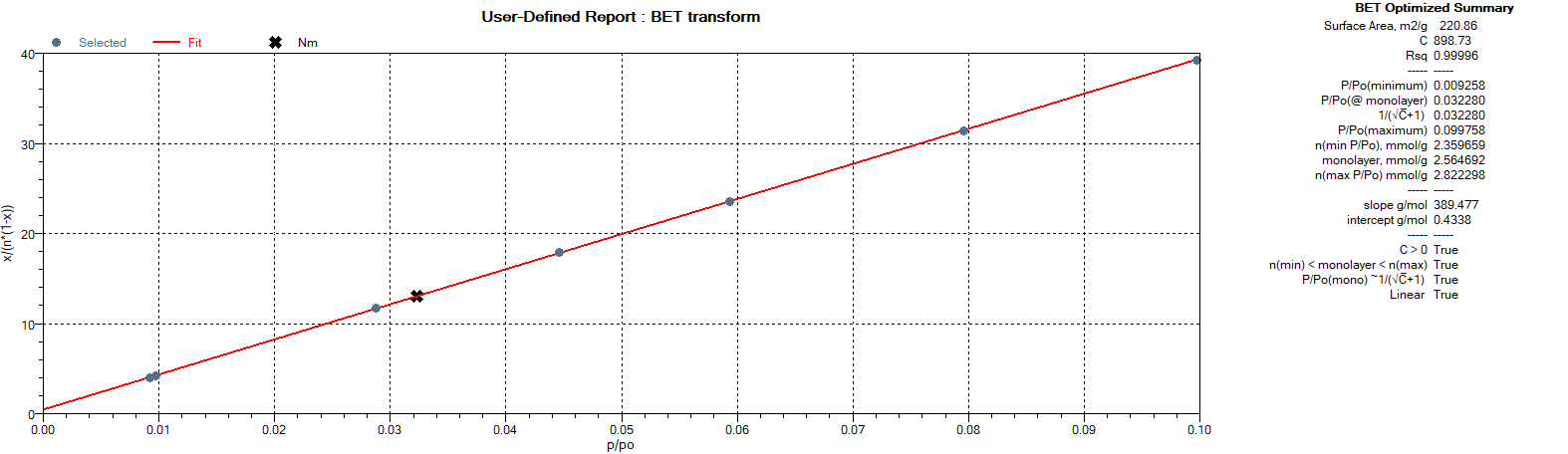


**Figure S29.** BET plot of F4_MIL-140A(Ce)_MeOH:H_2_O obtained by applying Rouquerol consistency criteria.


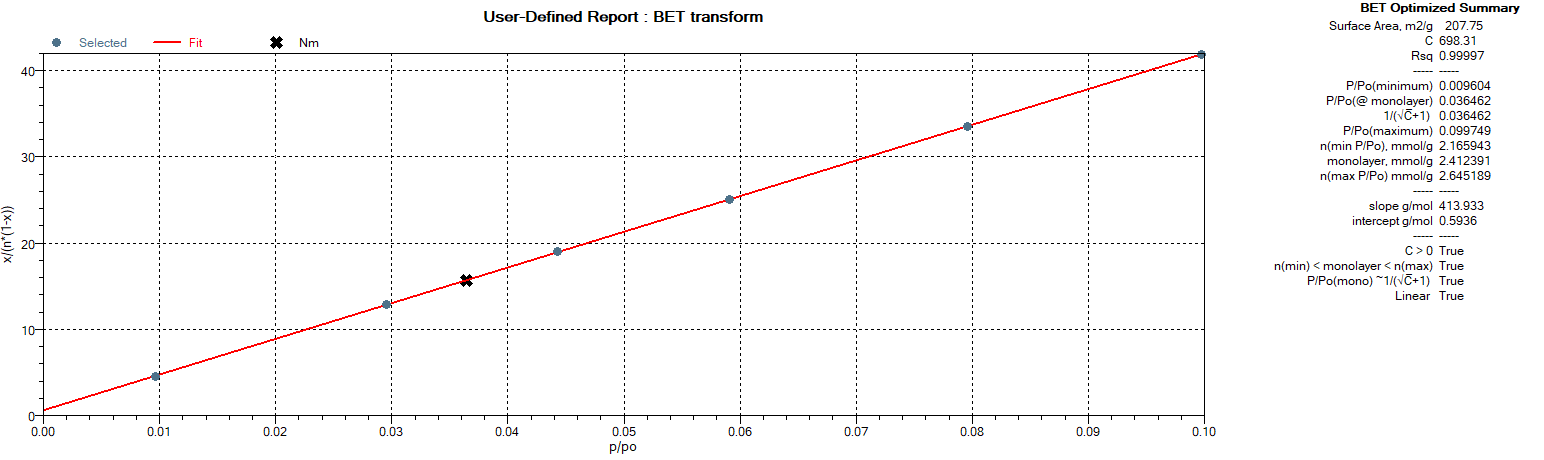


**Figure S30.** BET plot of F3_MIL-140A(Ce) obtained by applying Rouquerol consistency criteria.


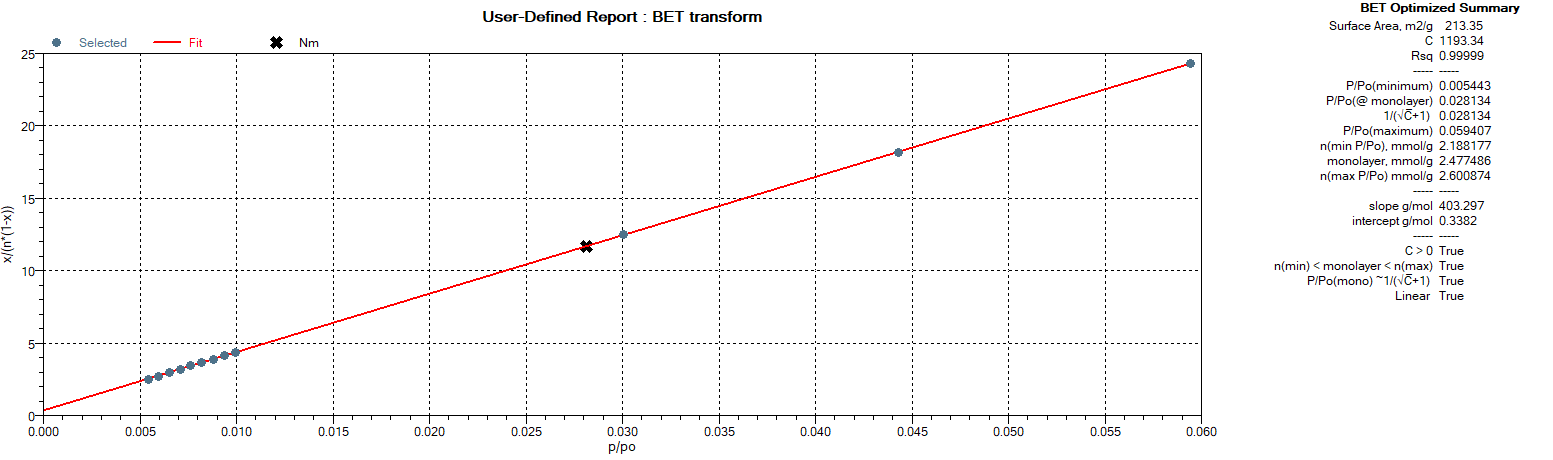


**Figure S31.** BET plot of *p*F2_MIL-140A(Ce) obtained by applying Rouquerol consistency criteria.

**Table S6.** Indication of the presence of a step-shaped profile in the CO_2_ adsorption isotherms and CO₂ uptake at 1.1 bar for F*x*_MIL-140A(Ce) materials.

| **MOF** | **Step-shaped Profile** | **Uptake at 1.1 bar**  **(mmol g^-1^)** |
| --- | --- | --- |
| F4_MIL-140A(Ce) reference | ✓ | 2.5 |
| F4_MIL-140A(Ce)_MeOH:H_2_O | ✓ | 2.2 |
| F3_MIL-140A(Ce) | X | 1.8 |
| *p*F2_MIL-140A(Ce) | X | 1.7 |

**S6. Variable temperature powder X-ray diffraction (VT-PXRD)**


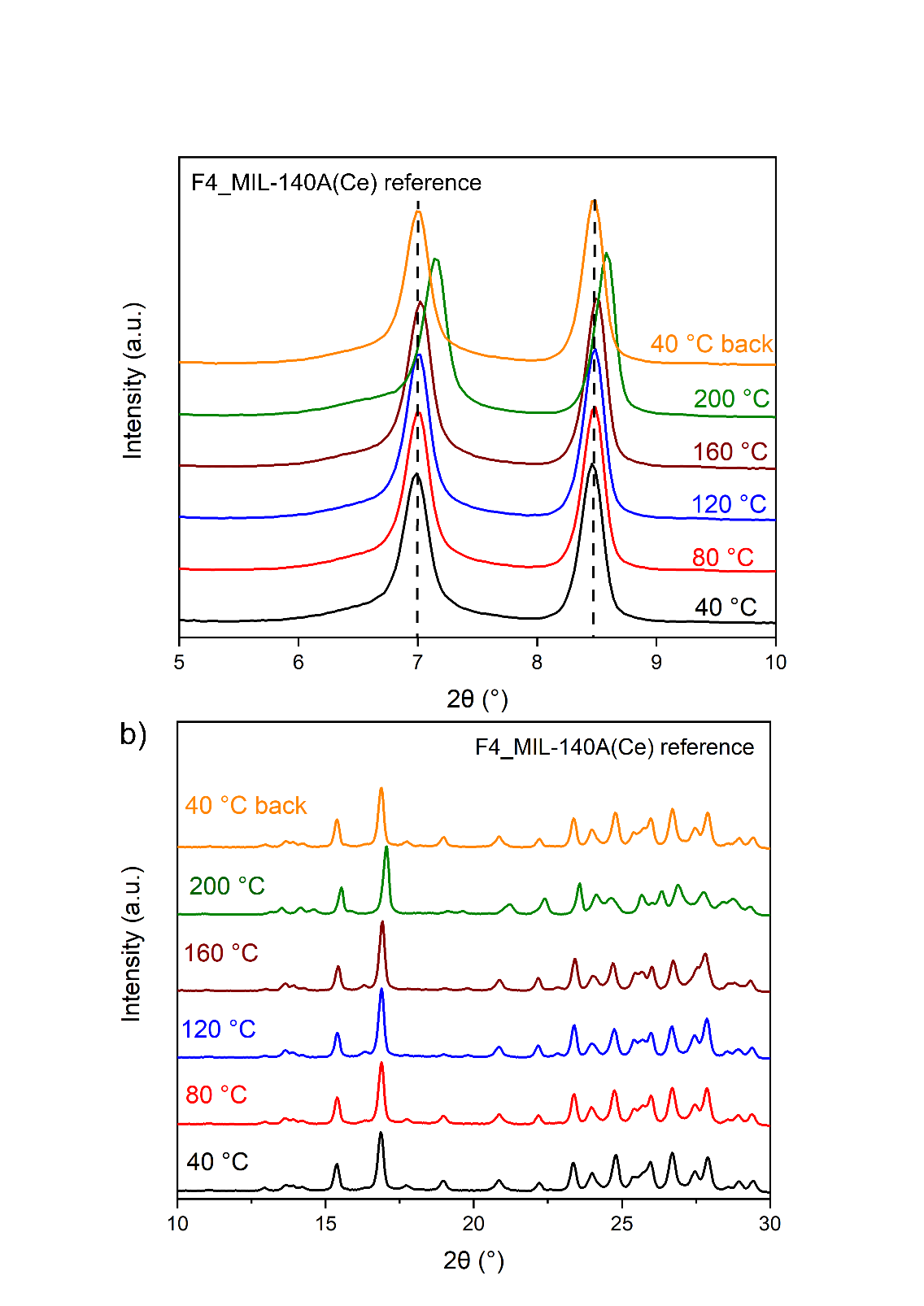


**Figure S32.** High angle region (a) and low angle region (b) of VT-PXRD patterns of F4_MIL-140A(Ce) reference.


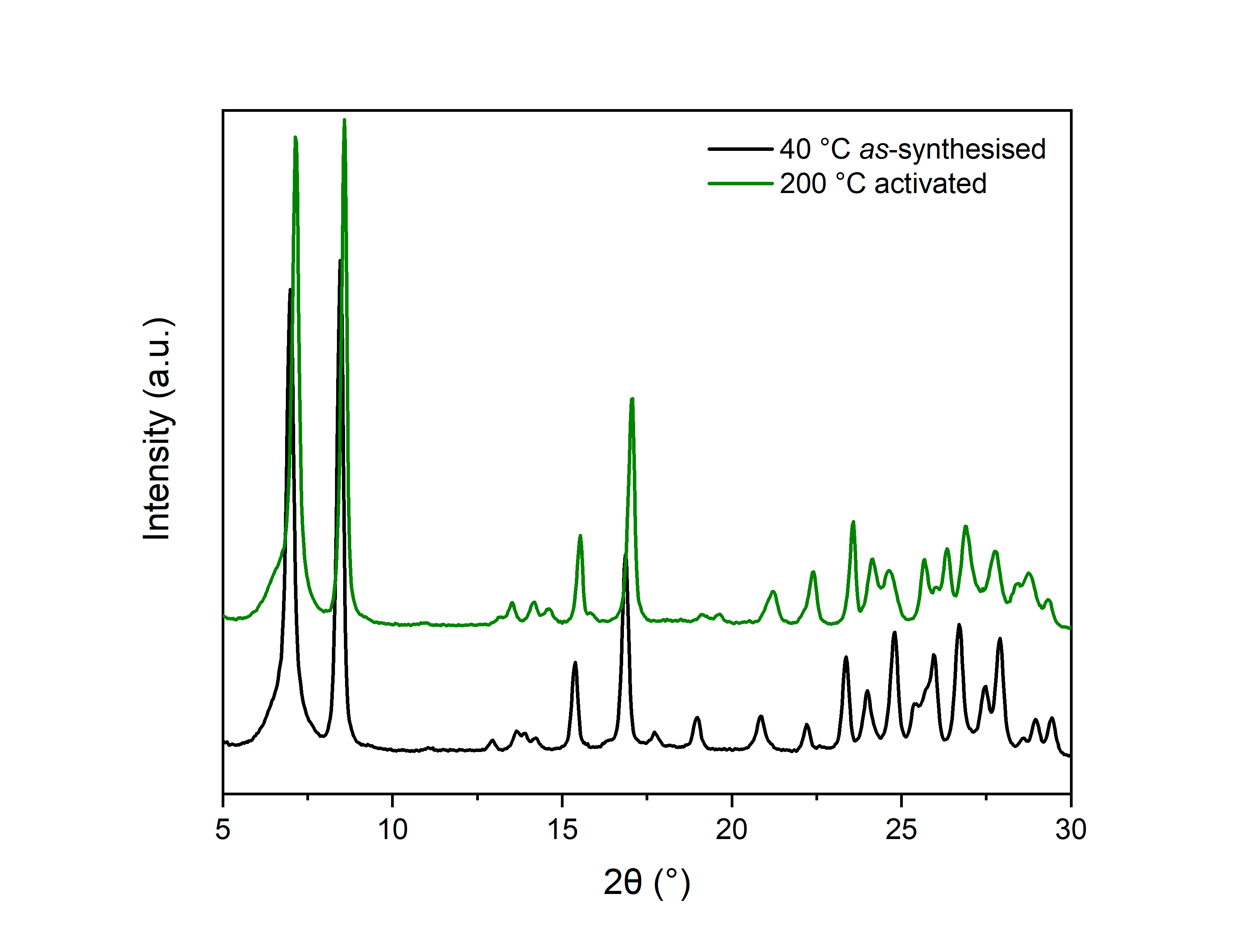


**Figure S33.** Comparison of the PXRD patterns of evacuated (green) and as*-*synthesised (black) F4_MIL-140A(Ce) reference.


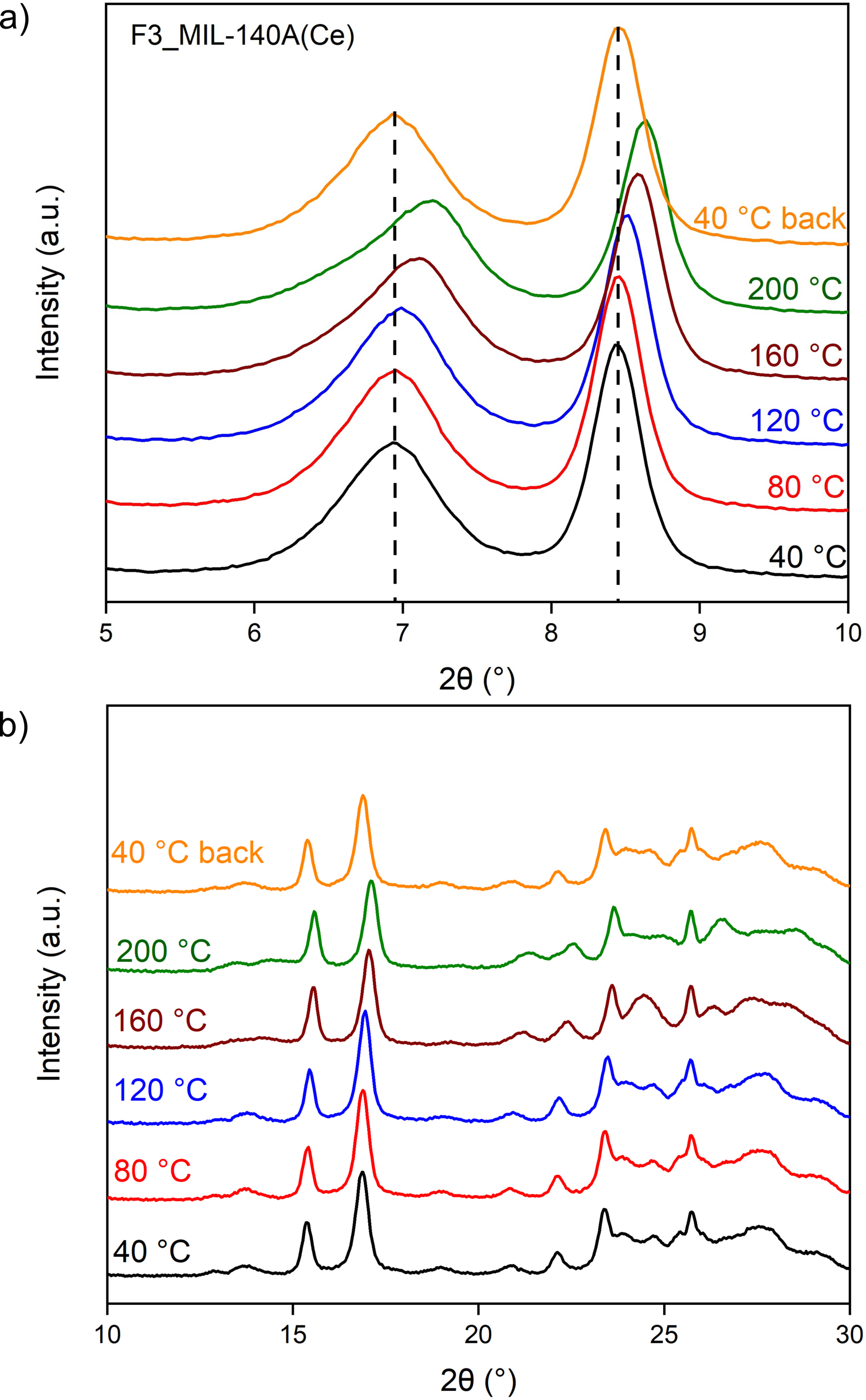


**Figure S34.** High angle region (a) and low angle region (b) of VT-PXRD patterns of F3_MIL-140A(Ce).


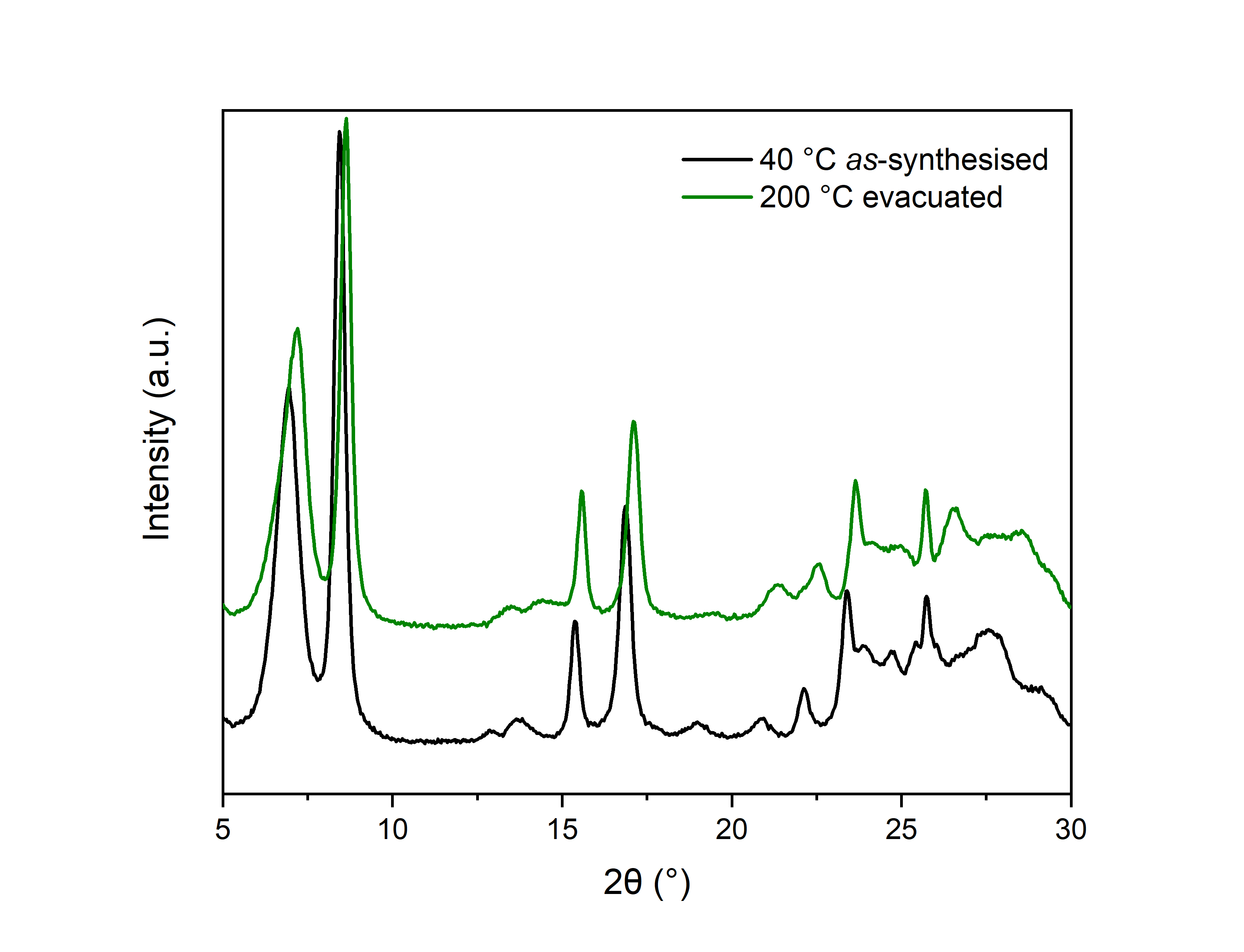


**Figure S35.** Comparison of the PXRD patterns of evacuated (green) and as*-*synthesised (black) F3_MIL-140A(Ce).


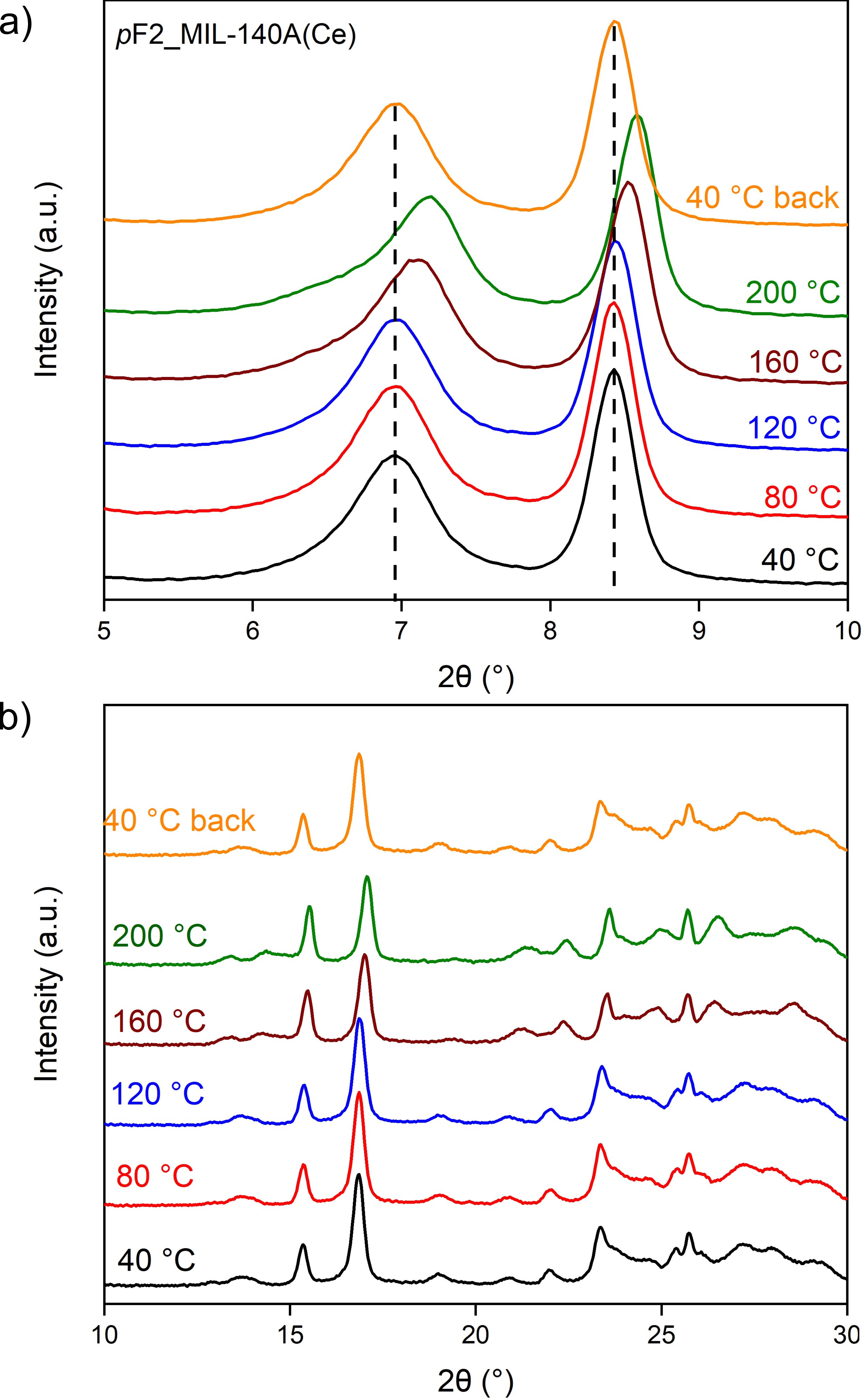


**Figure S36.** High angle region (a) and low angle region (b) of VT-PXRD patterns of *p*F2_MIL-140A(Ce).


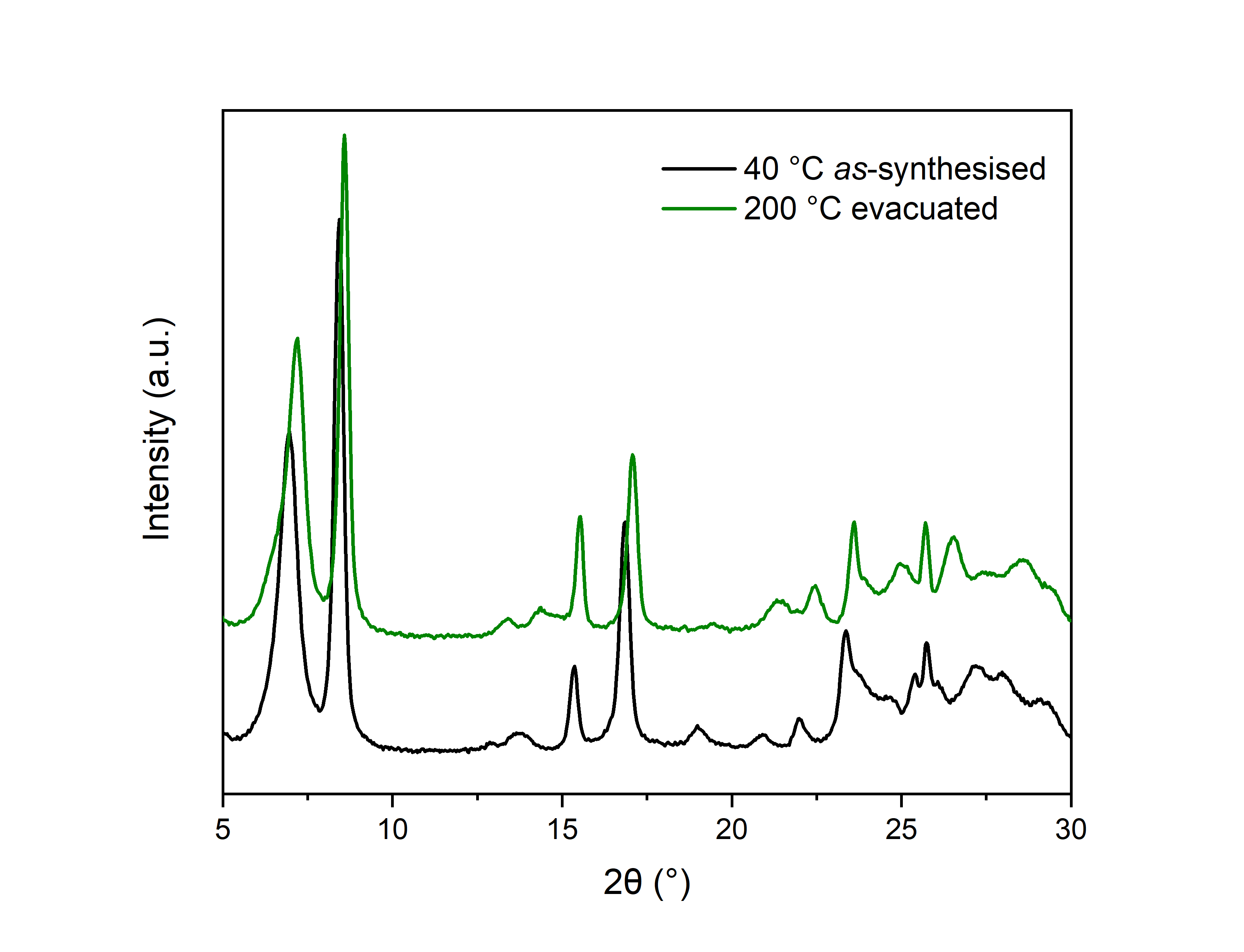


**Figure S37.** Comparison of the PXRD patterns of evacuated (green) and as*-*synthesised (black) *p*F2_MIL-140A(Ce).

**S7. *In situ* IR spectroscopy**

A qualitative band fitting of the IR spectra of CO-loaded F4_MIL-140A(Ce) reference, F3_MIL-140A(Ce), and *p*F2_MIL-140A(Ce) was carried out with the sole purpose of demonstrating the existence of multiple CO-Ce^IV^ adducts in the spectra of less fluorinated MOFs (**Figures S38-S40**). The fitted spectrum of CO-loaded F4_MIL-140A(Ce) reference (**Figure S38**) reveals three distinct components at 2153, 2137, and 2131 cm⁻¹. The weak bands at 2137 and 2131 cm⁻¹, detected also for F3_MIL-140A(Ce) and *p*F2_MIL-140A(Ce), are attributed to a liquid-like CO phase, condensing in the interparticle regions of the MOF, and to the interaction between CO and Ce^III^ sites, respectively.^8^ The assignment of these spectral components is straightforward, as also proved by their spectral behaviour upon CO outgassing (**Figure 6**, outgassing sequence). Indeed, by decreasing the CO pressure, the band ascribed to physisorbed CO immediately disappears, while the component generated by Ce^III^-CO adducts is still visible at low CO coverages, even if with a very weak intensity, and it has the same persistence of the stronger band ascribed to Ce^IV^-CO species. In F4_MIL-140A(Ce) reference, the sharp and symmetric component at 2153 cm⁻¹ is ascribed to the interaction of CO with a single population of Ce^IV^ sites. In contrast, at least two different spectral components at 2153 and 2148 cm⁻¹ and at 2149 and 2143 cm⁻¹, respectively, are needed to fit the band arising from Ce^IV^-CO species in F3_MIL-140A(Ce) and *p*F2_MIL-140A(Ce) (**Figure S39** and **Figure S40**), respectively, indicating the presence of distinct accessible Ce^IV^ species, likely arising from the disordered arrangement of the linkers within the framework.


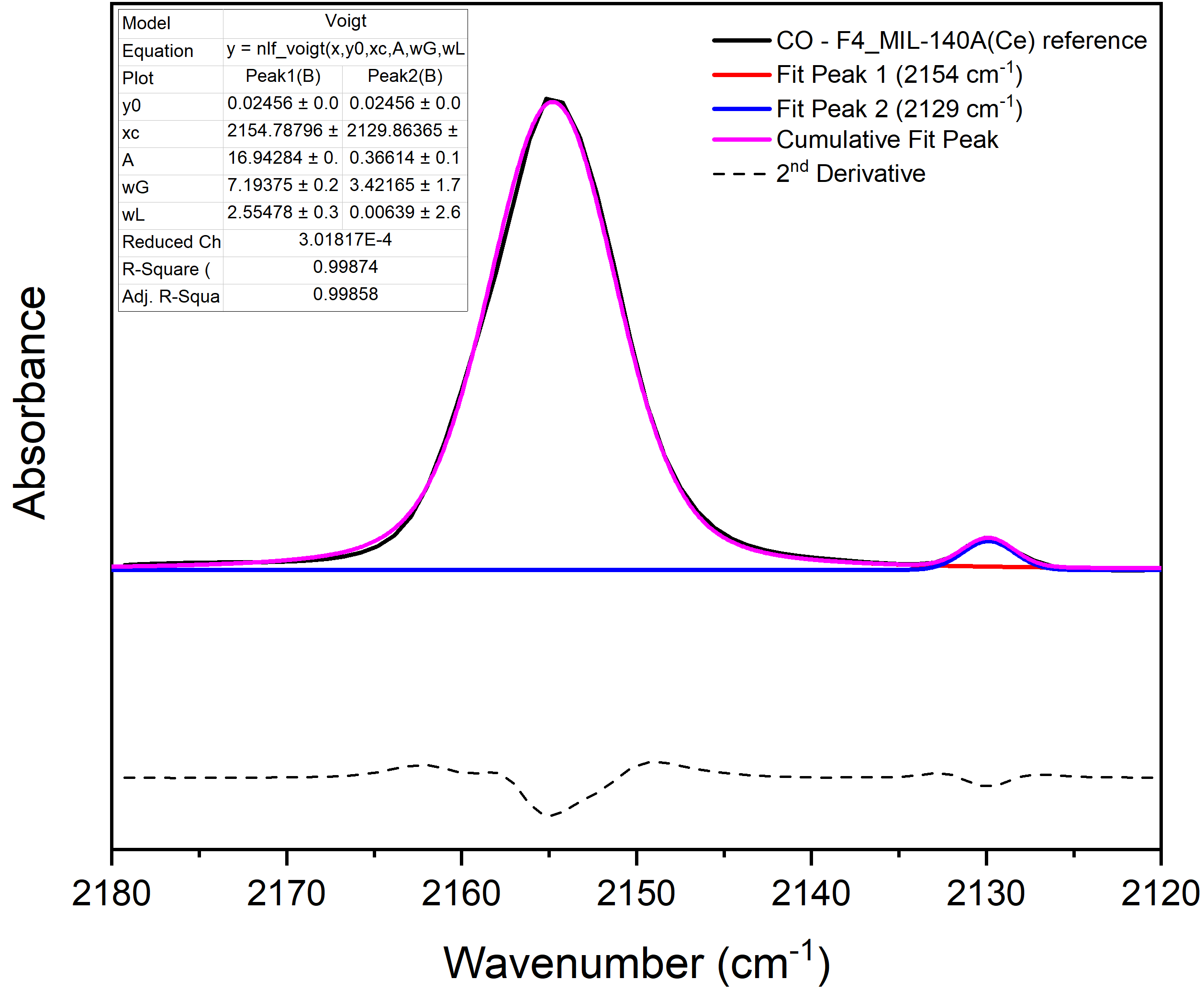


**Figure S38.** Band fitting of the IR spectrum of CO-loaded F4_MIL-140A(Ce) reference (R^2^ = 0.99874).

**
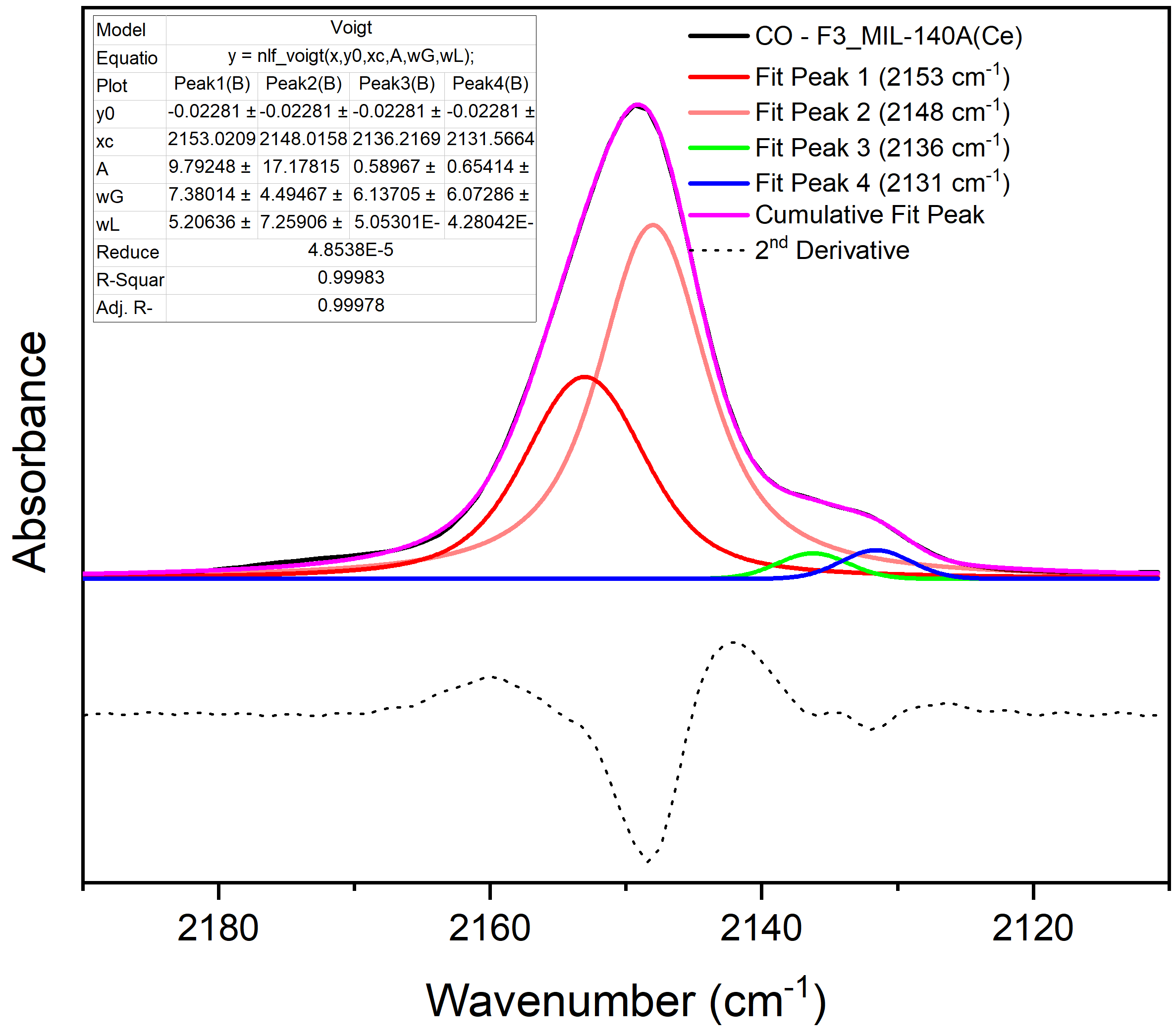
**

**Figure S39.** Band fitting of the IR spectrum of CO-loaded F3_MIL-140A(Ce) (R^2^ = 0.99983).

**
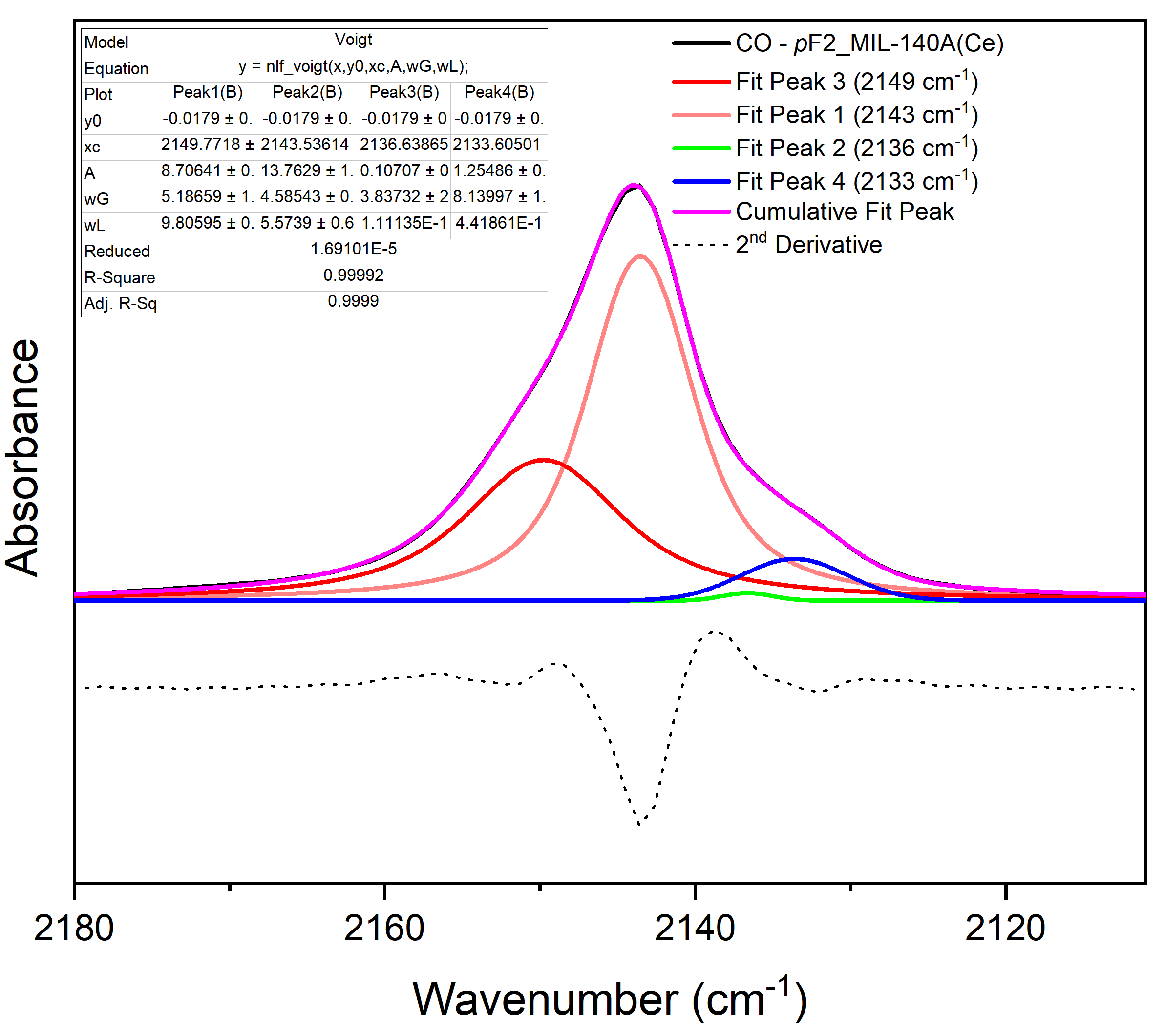
**

**Figure S40.** Band fitting of the IR spectrum of CO-loaded *p*F2_MIL-140A(Ce) (R^2^ = 0.99992).

**S8. CO_2_ adsorption microcalorimetry**

Microcalorimetric techniques allow the direct determination of the differential molar heat of adsorption q_diff_ at different coverages of the adsorptive probe, as reported in detail in section S1. For rigid frameworks, the differential heat is directly related to the exothermic adsorbate/adsorbent interactions. In contrast, for flexible frameworks, it represents a combination of the endothermic events generated by possible structural changes at different adsorbate coverages, and the exothermic adsorption.^7,13^ Due to instrumental constraints, the microcalorimetric measurements can only be collected between 30 and 300 °C, preventing a direct comparison of the volumetric CO_2_ adsorption isotherms collected by the microcalorimetric setup (**Figure S41**) with those obtained at 0 °C with the standard automatic volumetric apparatus (**Figure 3**). Both F3_MIL-140A(Ce) and *p*F2_MIL-140A(Ce) show standard Type I adsorption isotherms also at 30 °C, but with a lower adsorption capacity than at 0 °C, a typical consequence of the exothermic nature of the adsorption phenomenon.


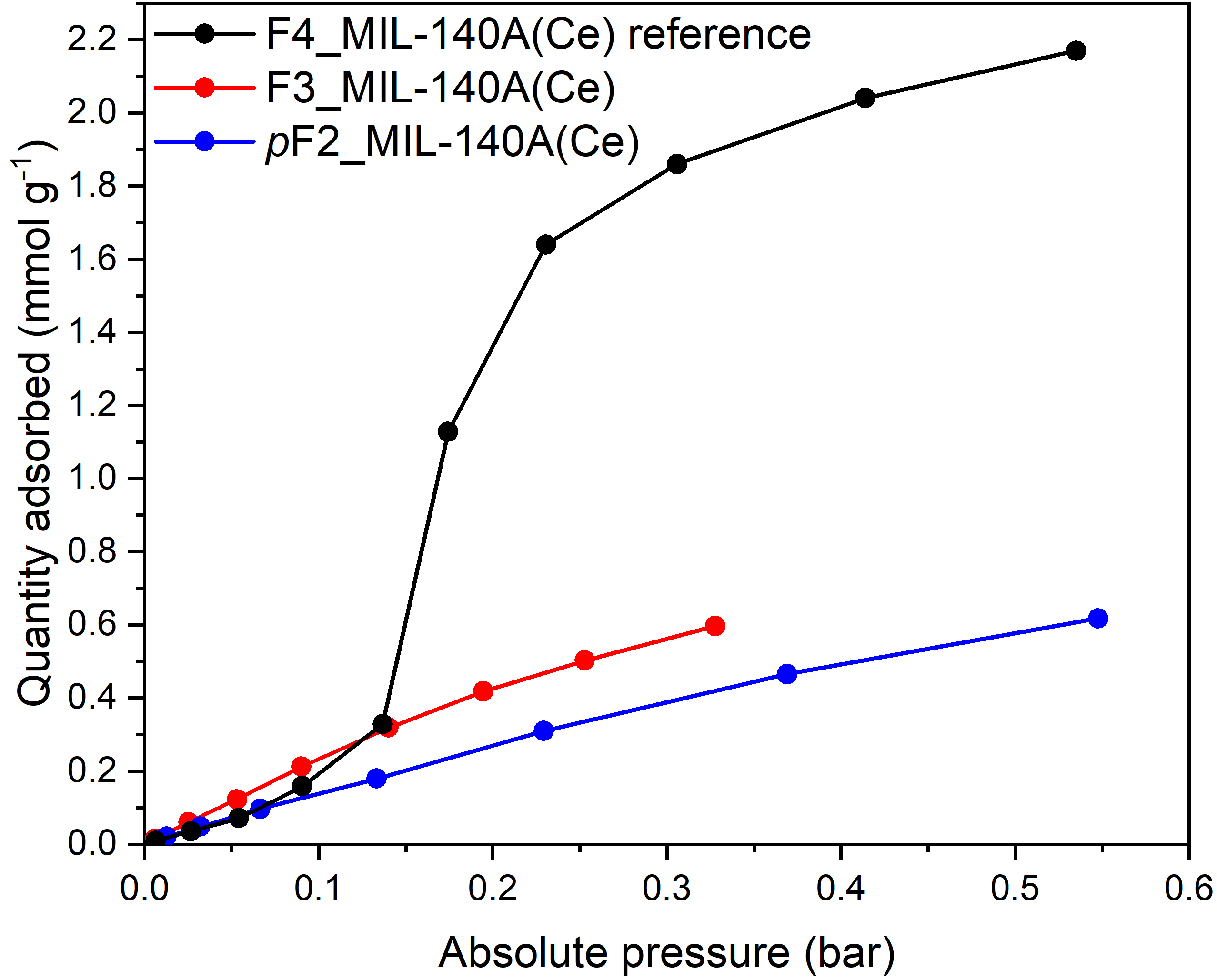


**Figure S41.** Volumetric CO_2_ adsorption isotherms collected at 30 °C on F4_MIL-140A(Ce) reference (black circles), F3_MIL-140A(Ce) (red circles) and *p*F2_MIL-140A(Ce) (blue circles) with a microcalorimetric apparatus.

**S9. CO_2_ dynamics in F3_MIL140A(Ce) by SSNMR**

The ^13^C static SSNMR spectra recorded on ^13^CO_2_-loaded F3_MIL140A(Ce) are shown in **Figure 8**. A line shape analysis of the static spectrum at -25 °C indicates that the powder pattern is characterized by an isotropic chemical shift δ_iso_ = 124.7 ppm, a span Ω = 66 ppm, and a skew κ = -0.8, with δ_iso_ = (δ_11_ + δ_22_ + δ_33_)/3, Ω = δ_11_ - δ_33_, and κ = (δ_22_ – δ_iso_)/ Ω and the chemical shift tensor components defined as δ_11_ ≥ δ_22_ ≥ δ_33_.^1,14^ These parameters are different from those of the CO_2_ ^13^C nucleus in the solid state, which shows an axially symmetric chemical shift tensor, dictated by the axial symmetry of the CO_2_ molecule, with δ_iso_ = 125 ppm, Ω = 315−335 ppm, and κ = 1.^3,4^ In particular, the much lower span value with respect to solid CO_2_ and the negative skew value indicate that in F3_MIL-140A(Ce) CO_2_ undergoes a wobbling or hopping motion with an angle between the C_∞_ symmetry axis of CO_2_ and the motion axis larger than the magic angle (54.74°), which partially average the chemical shift anisotropy (CSA) of the CO_2_ ^13^C nuclei. A similar situation was reported for CO_2_ in F4_MIL-140A(Ce),^15^ although with important differences. In fact, in F3_MIL-140A(Ce) the CO_2_ powder pattern has a smaller Ω and no evolution of the anisotropic line shape is observed with temperature, indicating that CO_2_ motions have a different geometry and always occur in the fast regime (*i.e.*, with a motional rate ≥ 10^6^ s^-1^) in the investigated temperature range. CO_2_ coordinated to the Ce open metal site experiences a confined environment, which imposes a single orientation to all molecules at low temperature (−25 °C). By assuming that CO_2_ in F3_MIL-140A(Ce) undergoes the same kinds of motions found in F4_MIL-140A(Ce), the spectral line shape can be reproduced by considering the translational hopping motion in the fast regime with *θ* = 81° and *φ* = 96°, as in F4_MIL-140A(Ce), superimposed to a local fast reorientational motion in a cone (wobbling) with an angle *ψ* of 40° (inset of **Figure 8**). In F4_MIL-140A(Ce), *θ* is the angle between the crystallographic *c* axis and the CO_2_ C_∞_ axis and φ is the reorientation angle of the CO_2_ axis from one site to another along the *c* axis. Within this hypothesis, the different environment of CO_2_ in F3_MIL-140A(Ce) is reflected in a less confined local motion than in F4_MIL-140A(Ce), most probably because of a lower steric hindrance of the less fluorinated linkers. On the other hand, the higher rate of the hopping motion at low temperature could be related to a lower activation energy for this motion in F3_MIL-140A(Ce), possibly associated with a less tight environment of CO_2_ on the Ce^IV^ sites. At temperatures ≥ -15 °C, the contribution of the anisotropic component to the spectrum decreases progressively in favor of an isotropic component. The evolution of the ^13^C spectral line shape with temperature could arise from the presence of two different local microenvironments for CO_2_ within the framework: one more restricted and completely populated at the lowest temperature here investigated, one less restricted and progressively populated when temperature is raised. On the basis of the Langmuir shape of the adsorption isotherm, we can envisage a migration of CO_2_ from the Ce^IV^ sites to the mesopores. By plotting the ratio between the populations of CO_2_ in the two environments as a function of inverse temperature (**Figure S42**), an exponential trend is found, which can be satisfactorily reproduced by a vant’Hoff equation with an enthalpy of about 28 kJ/mol. However, it is not excluded that the observed line shape evolution is associated with a so-called “apparent phase transition” between the end situations (fully isotropic vs fully anisotropic) protracted over a broad temperature range, generally observed in the presence of broad distributions of correlation times for the reorientational motion of the guest molecule in many differently restricted local microenvironments.^16–19,19^


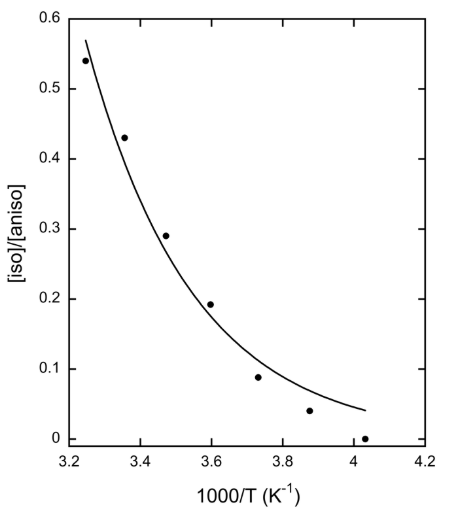


**Figure S42.** Ratio of CO_2_ molecules undergoing isotropic and anisotropic dynamics *vs* inverse temperature. The ratio was determined from the integrals of the isotropic and anisotropic signals in the ^13^C static SSNMR spectra reported in **Figure 8**.

**S10. References**

(1) Harris, R. K.; Becker, E. D.; Cabral De Menezes, S. M.; Goodfellow, R.; Granger, P. NMR Nomenclature: Nuclear Spin Properties and Conventions for Chemical Shifts. *Solid State Nucl. Magn. Reson.* **2002**, *22* (4), 458–483. https://doi.org/10.1006/snmr.2002.0063.

(2) Vold, R. L.; Hoatson, G. L. Effects of Jump Dynamics on Solid State Nuclear Magnetic Resonance Line Shapes and Spin Relaxation Times. *J. Magn. Reson.* **2009**, *198* (1), 57–72. https://doi.org/10.1016/j.jmr.2009.01.008.

(3) Beeler, A. J.; Orendt, A. M.; Grant, D. M.; Cutts, P. W.; Michl, J.; Zilm, K. W.; Downing, J. W.; Facelli, J. C.; Schindler, M. S.; Kutzelnigg, W. Low-Temperature Carbon-13 Magnetic Resonance in Solids. 3. Linear and Pseudolinear Molecules. *J. Am. Chem. Soc.* **1984**, *106* (25), 7672–7676. https://doi.org/10.1021/ja00337a003.

(4) Bowers, C. R.; Long, H. W.; Pietrass, T.; Gaede, H. C.; Pines, A. Cross Polarization from Laser-Polarized Solid Xenon to ^13^CO_2_ by Low-Field Thermal Mixing. *Chem. Phys. Lett.* **1993**, *205* (2–3), 168–170. https://doi.org/10.1016/0009-2614(93)89223-5.

(5) Rouquerol, J.; Llewellyn, P.; Rouquerol, F. Is the Bet Equation Applicable to Microporous Adsorbents? In *Studies in Surface Science and Catalysis*; Elsevier, 2007; Vol. 160, pp 49–56. https://doi.org/10.1016/S0167-2991(07)80008-5.

(6) Crocella’, V.; Atzori, C.; Signorile, M. A Kit for Volumetric Measurements of Gas Adsorption. WO Pat. 2021181211A1.

(7) Atzori, C.; Porcaro, N. G.; Crocellà, V.; Bonino, F.; Signorile, M.; Antico, P.; Daniel, C.; Venditto, V.; Grande, C. A.; Bordiga, S. Tailoring Novel Polymer/UTSA-16 Hybrid Aerogels for Efficient CH_4_/CO_2_ Separation. *Microporous Mesoporous Mater.* **2022**, *341*, 112106. https://doi.org/10.1016/j.micromeso.2022.112106.

(8) Cavallo, M.; Atzori, C.; Signorile, M.; Costantino, F.; Venturi, D. M.; Koutsianos, A.; Lomachenko, K. A.; Calucci, L.; Martini, F.; Giovanelli, A.; Geppi, M.; Crocellà, V.; Taddei, M. Cooperative CO_2_ Adsorption Mechanism in a Perfluorinated Ce^IV^ -Based Metal Organic Framework. *J. Mater. Chem. A* **2023**, *11* (11), 5568–5583. https://doi.org/10.1039/D2TA09746J.

(9) Kim, S.; Yoon, T.-U.; Oh, K. H.; Kwak, J.; Bae, Y.-S.; Kim, M. Positional Installation of Unsymmetrical Fluorine Functionalities onto Metal–Organic Frameworks for Efficient Carbon Dioxide Separation under Humid Conditions. *Inorg. Chem.* **2020**, *59* (24), 18048–18054. https://doi.org/10.1021/acs.inorgchem.0c02496.

(10) Meek, S. T.; Perry, J. J.; Teich-McGoldrick, S. L.; Greathouse, J. A.; Allendorf, M. D. Complete Series of Monohalogenated Isoreticular Metal–Organic Frameworks: Synthesis and the Importance of Activation Method. *Cryst. Growth Des.* **2011**, *11* (10), 4309–4312. https://doi.org/10.1021/cg201136k.

(11) D’Amato, R.; Donnadio, A.; Carta, M.; Sangregorio, C.; Tiana, D.; Vivani, R.; Taddei, M.; Costantino, F. Water-Based Synthesis and Enhanced CO_2_ Capture Performance of Perfluorinated Cerium-Based Metal–Organic Frameworks with UiO-66 and MIL-140 Topology. *ACS Sustain. Chem. Eng.* **2019**, *7* (1), 394–402. https://doi.org/10.1021/acssuschemeng.8b03765.

(12) Jacobsen, J.; Wegner, L.; Reinsch, H.; Stock, N. Ce-MIL-140: Expanding the Synthesis Routes for Cerium( iv ) Metal–Organic Frameworks. *Dalton Trans.* **2020**, *49* (32), 11396–11402. https://doi.org/10.1039/D0DT02455D.

(13) Llewellyn, P. L.; Maurin, G. Gas Adsorption Microcalorimetry and Modelling to Characterise Zeolites and Related Materials. *Comptes Rendus Chim.* **2005**, *8* (3–4), 283–302. https://doi.org/10.1016/j.crci.2004.11.004.

(14) Harris, R. K.; Becker, E. D.; De Menezes, S. M. C.; Granger, P.; Hoffman, R. E.; Zilm, K. W. Further Conventions for NMR Shielding and Chemical Shifts (IUPAC Recommendations 2008): International Union of Pure and Applied Chemistry Physical and Biophysical Chemistry Division. *Magn. Reson. Chem.* **2008**, *46* (6), 582–598. https://doi.org/10.1002/mrc.2225.

(15) Nardelli, F.; Nerli, F.; Della Latta, E.; Martini, F.; Geppi, M.; Taddei, M.; Calucci, L. Unveiling CO_2_ Dynamics in Perfluorinated Cerium-Based Metal–Organic Frameworks with UiO-66 and MIL-140 Topologies by Solid State NMR. *J. Phys. Chem. C* **2024**, *128* (16), 6887–6896. https://doi.org/10.1021/acs.jpcc.4c00483.

(16) Resing, H. A. Apparent Phase—Transition Effect in the NMR Spin—Spin Relaxation Time Caused by a Distribution of Correlation Times. *J. Chem. Phys.* **1965**, *43* (2), 669–678. https://doi.org/10.1063/1.1696791.

(17) Böhmer, R.; Diezemann, G.; Hinze, G.; Rössler, E. Dynamics of Supercooled Liquids and Glassy Solids. *Prog. Nucl. Magn. Reson. Spectrosc.* **2001**, *39* (3), 191–267. https://doi.org/10.1016/S0079-6565(01)00036-X.

(18) Buntkowsky, G.; Breitzke, H.; Adamczyk, A.; Roelofs, F.; Emmler, T.; Gedat, E.; Grünberg, B.; Xu, Y.; Limbach, H.-H.; Shenderovich, I.; Vyalikh, A.; Findenegg, G. Structural and Dynamical Properties of Guest Molecules Confined in Mesoporous Silica Materials Revealed by NMR. *Phys. Chem. Chem. Phys.* **2007**, *9* (35), 4843. https://doi.org/10.1039/b707322d.

(19) Della Latta, E.; Della Croce, F.; Bizai, G.; Murelli, A.; Lippi, M.; Rossi, P.; Cametti, M.; Paoli, P.; Martini, F.; Calucci, L.; Geppi, M. Dynamics of Ligand and Guest in 1D Hg(II)‐Bispidine Coordination Polymers With Different Topologies Investigated by Solid‐State NMR. *Chem. – Eur. J.* **2025**, *31* (43), e202501458. https://doi.org/10.1002/chem.202501458.
